# An empirical assessment of differential privacy in real-world observational data: a case-control study of asthma exacerbation in UK Biobank linked with electronic health records

**DOI:** 10.1093/jamia/ocaf090

**Published:** 2025-06-18

**Authors:** Mehrdad A Mizani, Aziz Sheikh, Amitava Banerjee

**Affiliations:** Institute of Health Informatics, University College London, London NW1 2DA, United Kingdom; Nuffield Department of Primary Care Health Sciences, University of Oxford, Oxford OX2 6GG, United Kingdom; Institute of Health Informatics, University College London, London NW1 2DA, United Kingdom

**Keywords:** differential privacy, patient data privacy, observational studies, case-control studies, matched case-control studies

## Abstract

**Objectives:**

Electronic health records (EHRs) provide substantial resources for observational studies, yet present significant challenges in safeguarding patient privacy while maintaining research quality. Differential privacy (DP) offers a quantifiable privacy guarantee; however, its impact on observational studies remains underexplored. We empirically evaluated the effects of DP across varying values of its privacy parameter, epsilon, on case-control analysis outcomes using EHR data. This study aims to inform DP parameter selection and examines the influence of study characteristics on differentially private observational studies.

**Materials and Methods:**

We assessed the effects of DP on a case-control study of 1-year asthma exacerbations, including 22 165 participants with a history of asthma from UK Biobank linked to EHR data. Odds ratios (ORs) for sociodemographic factors and comorbidities were analyzed using adjusted and propensity score-matched models across epsilon values.

**Results:**

DP influenced the magnitude, direction, and statistical significance of ORs, occasionally resembling patterns of misclassification, residual confounding, and false-positive bias. Rare and imbalanced covariates showed greater OR variability, especially in matched studies. Epsilons smaller than ln(2) led to noticeable OR fluctuations.

**Discussion:**

The impact of DP on ORs and selection of an optimal epsilon depends on sample size, covariate prevalence, confounders, case-to-control ratios in propensity score matching, mitigation of random seed *p*-hacking, and trust models.

**Conclusion:**

The effects of DP on ORs are highly context-dependent. In this study, epsilon values below ln(2) led to unstable ORs across random seeds. Averaging results or using predetermined seeds may help reduce variability and mitigate *p*-hacking.

## Introduction

### Background and significance

The expansion of real-world healthcare data offers substantial resources for observational studies, clinical research, and public health interventions.^1^^,^^2^ However, preserving patient privacy while ensuring data quality remains a persistent challenge.^3^^,^^4^ Privacy protection in healthcare datasets is multifaceted, encompassing technical measures, trust models, data access protocols, and output disclosure control.^5^^,^^6^ In the United Kingdom, the “Five Safes” framework is widely adopted by data holders and Secure Data Environments (SDEs), emphasizing access by trusted researchers (safe people), ethical research (safe projects), secure environments (safe settings), protecting against individual identification (safe data), and privacy-preserving outputs (safe outputs).^7–10^

Privacy protection requires disclosure control mechanisms to mitigate re-identification attacks (linking records to specific individuals), reconstruction attacks (inferring sensitive attributes), and tracing attacks (detecting an individual’s presence in a dataset).^11^ Common approaches to mitigate these risks include statistical disclosure control (SDC),^12^  *k*-anonymity,^13^ synthetic data,^14^ and differential privacy (DP).^15^ Synthetic data and *k*-anonymity alter microdata, whereas SDC and DP apply to both microdata and analysis outputs.^12^^,^^16^

SDC, the most common disclosure control method for safe outputs, includes techniques such as query restriction and perturbation.^4^^,^^12^^,^^17^ However, SDC lacks formal privacy guarantees and remains vulnerable to background knowledge or tracing attacks.^11^ To address these limitations, DP, proposed by Dwork et al. in 2006, has emerged as a mathematically rigorous approach for privacy protection across multiple domains, including healthcare.^15^^,^^18–20^ DP provides quantifiable privacy and is more resilient against tracing or reconstruction attacks.^11^^,^^21^^,^^22^ DP guarantees that the inclusion or exclusion of an individual’s data produces statistically indistinguishable outputs within a predefined privacy bound. This is achieved by adding random noise, typically from a Gaussian or a Laplace distribution, to introduce uncertainty about the contribution of any individual record.^23^ The noise is governed by epsilon,^24^ a parameter measuring privacy loss. Lower epsilon values increase noise, enhancing privacy while reducing data utility; higher values weaken privacy protection but improve accuracy.

Epsilon is a critical parameter for effective privacy protection and accurate results; however, determining its optimal value is not trivial.^23–26^ Optimizing epsilon becomes further complicated in observational research involving multiple iterative steps. A core property of DP, known as composition, states that when multiple differentially private outputs are generated from the same dataset, the cumulative privacy loss equals the sum of the epsilons associated with each output, thereby increasing total privacy loss and degrading overall privacy.^11^ The composition property allows for defining a total acceptable privacy loss or privacy budget.^24^ Dwork et al. in 2019 propose a publicly available Epsilon Registry to guide epsilon selection, total privacy budgeting, and other implementation decisions.^24^ The authors emphasize the importance of example implementations, shared learning, and simulations as effective tools for demonstrating the practical value of DP to non-technical audiences.

### DP in observational studies

DP has been successfully applied for disclosure control in census data, notably by the US Census Bureau.^27–29^ While its implications for census data are well studied, research has largely focused on generating differentially private microdata or descriptive epidemiological outputs.^30–33^

To examine the impact of DP on real-world observational studies, we conducted a scoping review (Tables SA1 and SA2), screening 1493 articles and identifying 6 primary studies and 1 systematic review that met the inclusion criteria. The systematic review revealed that DP was predominantly used in aggregated statistics, synthetic data, or predictive modeling, highlighting a gap in observational case studies.^19^ Additional articles explored the technical aspects of DP,^5^ its practical implications in healthcare,^34^ and applications in regression and survival analysis.^35^^,^^36^ One study examining logistic regression with both public and private data highlighted the significance of the privacy budget.^37^ Another empirical study on epidemiological research investigated the effects of DP on randomized clinical trials, emphasizing the need to assess its practical impacts on multidimensional, real-world data.^38^

### Objective

We now evaluate the application of DP in generating privacy-preserving outputs in case-control studies using multidimensional observational data. This study aims to inform the determination of the privacy budget by assessing the impact of study design and data characteristics on outcomes in DP-enabled case-control studies. We focus on global DP, which refers to the application of DP to analysis outputs rather than microdata, producing more reliable statistical results.^16^

Specifically, we investigated the impact of DP on risk factor effect sizes for 1-year asthma exacerbation using electronic health records (EHRs) of 22 165 adults with asthma in UK Biobank data. We analyzed how varying epsilon values influence odds ratios (OR) in adjusted and propensity score-matched models.^39^ The adjusted model serves as a baseline to illustrate the effects of DP across the entire cohort. The matched analysis extends this by examining the implications of DP, particularly for confounders and a smaller cohort.^40^ We employed multiple evaluation methods, including forest plots for ORs and 95% confidence intervals (CIs), SHapley Additive exPlanations (SHAP) values for variable importance,^41^ balance plots for propensity score matching, and model performance metrics. To our knowledge, this is one of the few empirical studies of the implications of DP for safeguarding outputs in observational research using EHRs, particularly extending to confounding adjustment, propensity score matching, and multiple evaluation approaches.

## Methods

### Data source and study population

We used UK Biobank data, a biomedical database comprising half a million participants aged 40-69 in 2006-2010, including lifestyle, healthcare, self-reported diagnosis, and genetic data.^42^ The dataset was linked to general practitioner (GP) records for 45% of the participants, based on non-randomized participation of GP practices, and to hospital inpatient data.^43–45^ The study cohort included 22 165 participants with GP records and at least 1 year of asthma history at UK Biobank enrollment, when baseline questionnaires and measurements were collected (Table 1, Figure SB1).

**Table 1.** Descriptive statistics of patients with at least 1 year of asthma history at UK Biobank enrollment; cases defined by asthma exacerbation within 1 year after enrollment.

		Entire cohort	Cases	Controls	Matched controls	
**Size, N (%)**		22 165 (100)	2714 (12.24)	19 451 (87.76)	2713 (12.24)	
**% Female**		58.90	64.07	58.18	63.47	
**Age (baseline), Mean (SD)**		56.73 (8.23)	58.65 (7.84)	56.47 (8.25)	58.71 (7.76)	
**Age bands** **N (%)**	40-49	5290 (23.87)	446 (16.43%)	4844 (24.90)	427 (15.74)	
	50-59	7175 (32.37)	789 (29.0)	6386 (32.83)	829 (30.56)	
	60-69	9234 (41.66)	1411 (51.99)	7823 (40.22)	1379 (50.83)	
	70-79	466 (2.10)	68 (2.51)	398 (2.05)	78 (2.88)	
**Asthma duration (years), Mean (SD)**		15.38 (12.77)	15.37 (13.00)	15.38 (12.74)	15.17 (13.06)	
**Late onset of asthma (at age ≥ 40)**		2287 (10.32)	1826 (67.28)	7699 (39.58)	1836 (67.67)	
**Ethnicity** **N (%)**	Asian	574 (2.59)	86 (3.17)	488 (2.51)	82 (3.02)	
	Black	84 (0.38)	9 (0.33)	75 (0.39)	5 (0.18)	
	White	20 948 (94.51)	2556 (94.18)	18 392 (94.55)	2549 (93.96)	
	Mixed	135 (0.61)	12 (0.44)	123 (0.63)	20 (0.74)	
	Other	300 (1.35)	38 (1.40)	262 (1.35)	38 (1.40)	
	Unknown	124 (0.56)	13 (0.48)	111 (0.57)	16 (0.59)	
**Non-White ethnicity, N (%)**		1217 (5.49)	158 (5.82)	1059 (5.44)	164 (6.05)	
**Comorbidity** **N (%)**	Anxiety	2561 (11.55)	367 (13.52)	2194 (11.28)	316 (11.65)	
	Body mass index ≥30	6770 (30.54)	1035 (38.13)	5735 (39.48)	829 (30.56)	
	Chronic kidney disease	168 (0.76)	34 (1.25)	137 (0.69)	24 (0.88)	
	Chronic obstructive pulmonary disease	1265 (5.71)	403 (14.85)	862 (4.43)	411 (15.15)	
	Cardiovascular disease	1265 (5.71)	209 (7.70)	1056 (5.43)	190 (7.00)	
	Atrial fibrillation	443 (2.00)	78 (2.87)	365 (1.88)	62 (2.29)	
	Acute myocardial infarction	254 (1.15)	40 (1.47)	214 (1.10)	46 (1.70)	
	Cardiomyopathy	47 (0.21)	7 (0.25)	40 (0.21)	5 (0.18)	
	Deep vein thrombosis	14 (0.06)	5 (0.18)	9 (0.05)	–	
	Heart failure	91 (0.4)	15 (0.55)	76 (0.39)	14 (0.52)	
	Peripheral artery disease	170 (0.77)	25 (0.92)	145 (0.75)	30 (1.11)	
	Pulmonary embolism	167 (0.74)	32 (1.18)	132 (0.68)	18 (0.66)	
	Stroke	328 (1.48)	48 (1.77)	280 (1.44)	53 (1.95)	
	Depression	2399 (10.82)	334 (12.31)	2065 (10.62)	321 (11.83)	
	Diabetes	437 (1.97)	55 (2.03)	382 (1.96)	52 (1.92)	
	Hypertension	4169 (18.81)	684 (25.20)	3485 (17.92)	568 (20.94)	
**Cardinal symptoms (1-year)**	Any	15 760 (71.10)	2393 (88.17)	13 367 (68.72)	1940 (71.51)	
	Chest pain or discomfort	6910 (31.17)	1172 (43.18)	5738 (29.50)	875 (32.25)	
	Shortness of breath	2205 (9.95)	520 (19.16)	1658 (8.66)	272 (10.03)	
	Wheezing	14 468 (65.27)	2302 (84.82)	12 166 (62.55)	1792 (66.05)	
**Smoking**	Non-smoker	12 608 (55.18)	1404 (49.97)	11 204 (55.91)	1417 (52.23)	
	Current smoker	2210 (9.67)	339 (12.06)	1871 (9.34)	332 (12.34)	
	Previous smoker	8030 (35.15)	1067 (37.97)	6963 (34.75)	964 (35.53)	
**1-year history of clinical asthma exacerbation**		393 (1.77)	175 (6.44)	218 (1.12)	162 (5.97)	
**1-year history of Prednisolone oral corticosteroid (OCS) prescription**		584 (2.63)	160 (5.89)	424 (2.18)	167 (6.16)	
**Deaths (during study period)**		42 (0.19)	10 (0.36)	32 (0.16)	–	
**Deaths (total)**		2287 (10.32)	439 (16.17)	1848 (9.50)	320 (11.80)	

### Variables and phenotypes

Sociodemographic variables, cardinal symptoms (chest pain or discomfort, shortness of breath, wheezing), and smoking status were extracted from UK Biobank fields recorded at enrollment. Comorbidities were identified using validated clinical codes (Tables SA3 and SA4). Self-reported disease histories were excluded to minimize recall bias.

### Study design

We conducted a case-control analysis comparing cases (ie, participants who experienced asthma exacerbation within 1 year after the index date, defined as UK Biobank enrollment) to controls without exacerbation during the same period, assessing associations with prior risk factors (Table SB1). Asthma exacerbation criteria, adapted from Mukherjee et al. for UK Biobank data, were (a) GP-recorded exacerbation codes, (b) asthma-related hospitalization, (c) asthma-related emergency admission, and (d) non-repeat prescription (Rx) of Prednisolone oral-corticosteroid (OCS).^46^ Deaths during the study period were included to mitigate survivorship bias.

We identified risk factors based on log-likelihood tests and SHAP values (Tables SA5-SA7), covering age ≥ 60, female sex, non-White ethnicity, and comorbidities including body mass index (BMI)≥30, anxiety, chronic kidney disease (CKD), chronic obstructive pulmonary disease (COPD), cardiovascular disease (CVD), diabetes, and hypertension. Additional factors considered were a history of cardinal symptoms, asthma exacerbation, and OCS Rx in the previous year.

### Statistical analysis

Logistic regression was utilized to estimate adjusted reference ORs and 95% CIs of asthma exacerbation risk factors. Covariates were dichotomized for parsimony. Propensity scores were calculated using baseline covariates of sex, ethnicity, COPD, asthma exacerbation, and OCS Rx in the previous year, smoking status, and normalized age and asthma duration. Matching was performed using a K-Nearest Neighbor (KNN) algorithm without replacement, applying a 0.1 caliper to ensure matches were within a predefined propensity score distance. The sample size (ie, number of cases and controls combined) included the full cohort in the adjusted analysis and varied in the matched analyses based on the case-to-control ratio (1:1 in the primary analysis and 1:2 and 1:4 in sensitivity analyses, where the impact of sample size was further examined).

### Differential privacy

Formally, as shown in the following formula, a random algorithm M is ϵ-differentially private if for all adjacent datasets $D_{1}$ and $D_{2}$, that differ in one record, and for all outputs S of M, the probability of generating a certain output from $D_{1}$ and $D_{2}$ differ by a factor exponentially relative to ϵ.^47^


P[M(D1)∈S]≤eϵ.P[M(D2)∈S]

We used IBM’s diffprivlib library to compute differentially private ORs.^48^ Noise in logistic regression was calibrated based on the maximum L2-norm, or largest Euclidean distance of any row in the dataset. We defined L2-norm as p, where p is the number of binary predictors and min-max normalized numerical predictors.

### Evaluation of the effects of DP

To evaluate the impact of epsilon on ORs, we used a set of approximately logarithmically spaced ϵ values: [10, 5, 2, ln(3), 1, ln(2), 0.25, 0.1, 0.05, 0.025, 0.01]. This range includes ln(2)=0.693 and ln(3)=1.098, which are commonly used in DP studies.^15^^,^^38^ The selected epsilons covered high [2-10], medium [0.25-ln(3)], and low [0.01-0.1] ranges. DP was applied to adjusted and matched ORs using a fixed random seed for reproducibility and consistency.

Forest plots and SHAP values were used to assess effect sizes and covariate importance in logistic regression. Overall model calibration and discrimination were evaluated using the Brier score and receiver operating characteristic (ROC) area under the curve (AUC).^49^ Balance plots were used to show the standardized mean difference (SMD) per ϵ value.

For sensitivity analysis, we examined the impact of excluding deaths, varying covariates and random seeds, and multiple case-to-control ratios.

### Reporting guidelines

The study follows the Strengthening of Reporting of Observational Studies in Epidemiology (STROBE) guidelines for case-control studies (Table SB2).^50^

## Results

### Evaluating effect sizes and SHAP values of reference models

**Figure 1.**
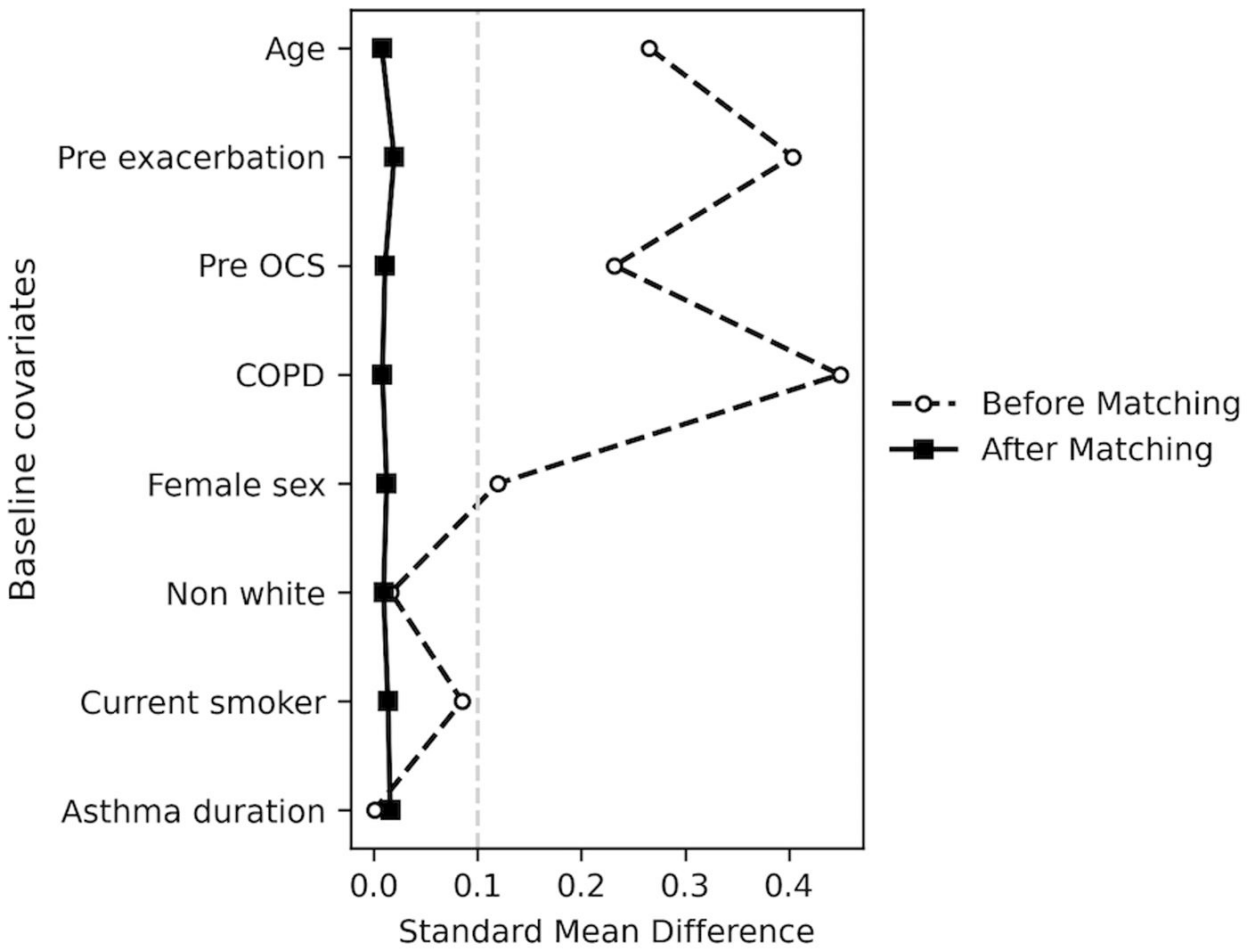
Balance plot of standardized mean differences (SMDs) for covariates before and after 1:1 matching.

In the entire cohort (2714 cases, 19 451 controls) and matched subset (2714 cases, 2713 controls matched with a 1:1 case-to-control ratio using KNN with a 0.1 caliper, without replacement), the most common risk factors (prevalence > 50%) included cardinal symptoms, female sex, and age ≥ 60, while rare factors (prevalence < 3%) were CKD and diabetes. In the adjusted model, the most imbalanced covariates were asthma exacerbation in the previous year, COPD, OCS Rx, and CKD, with a case-to-control prevalence ratio (ie, the ratio of covariate prevalence in cases to that in controls) of 5.76 (95% CI: 5.53-6.00), 3.35 (3.22-3.49), 2.71 (2.60-2.82), and 1.81 (1.74-1.88), respectively. After matching, baseline covariates were effectively balanced, with SMDs below 0.1, as shown in Figure 1. CKD showed the highest post-matching imbalance, with a prevalence ratio of 1.42 (95% CI: 1.35-1.50).

**Figure 2.**
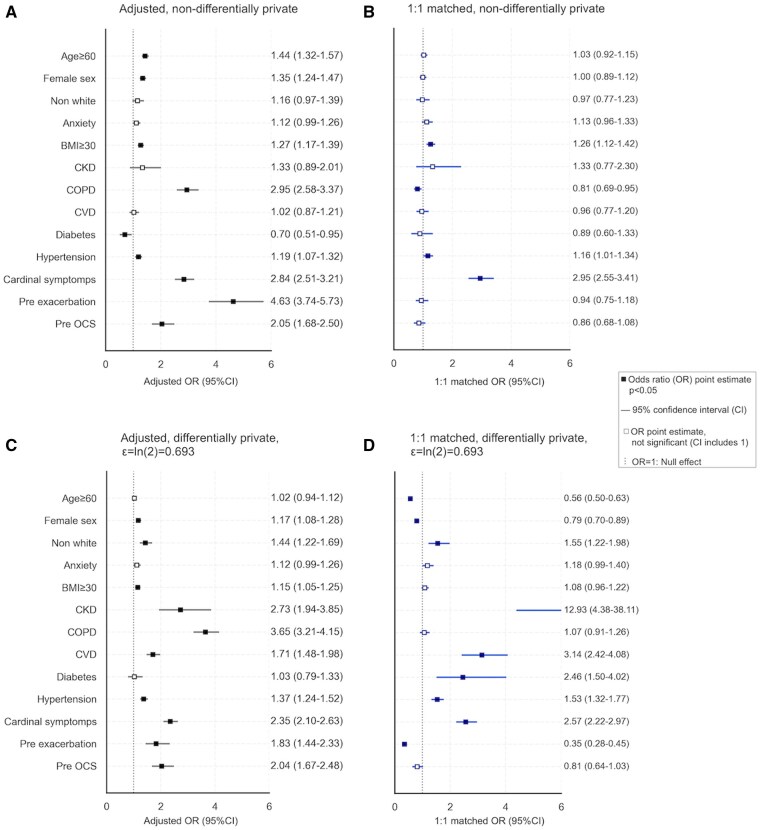
Odds ratios and 95% confidence intervals across four models: (A) Adjusted reference model, non-differentially private; (B) One-to-one matched model, non-differentially private; (C) Adjusted model with differential privacy, ϵ = *ln*(2); (D) One-to-one matched model with differential privacy, ϵ = *ln*(2).

Adjusted ORs (95% CI) for age ≥ 60 and female sex were 1.44 (1.32-1.57) and 1.35 (1.24-1.47), respectively (Figure 2A). COPD and cardinal symptoms had higher ORs of 2.95 (2.58-3.37) and 2.84 (2.51-3.21), while BMI ≥ 30 and hypertension had lower ORs of 1.27 (1.17-1.39) and 1.19 (1.07-1.32). One-year history of OCS Rx and asthma exacerbation had elevated ORs of 2.05 (1.68-2.50) and 4.63 (3.74-5.73). For rarer variables, diabetes showed a negative association with an OR of 0.70 (0.51-0.95), while CKD and CVD had non-significant ORs of 1.33 (0.89-2.01) and 1.02 (0.87-1.21). Anxiety and non-White ethnicity were not statistically significant, with ORs of 1.12 (0.99-1.26) and 1.16 (0.97-1.39), respectively.

In the matched analysis (Figure 2B), most baseline covariates used in propensity score calculation had non-significant ORs, except for COPD 0.81 (0.69-0.95), suggesting potential residual confounding. Significant post-matching ORs included cardinal symptoms (2.95, 2.55-3.41), BMI ≥ 30 (1.26, 1.12-1.42), and hypertension (1.16, 1.01-1.34).

Figure 2C and D show the ORs of the adjusted and matched models, respectively, for *ϵ=*ln(2) to illustrate the overall effect of DP at a mid-range and commonly used *ϵ*.^15^^,^^38^

### SHAP values

**Figure 3.**
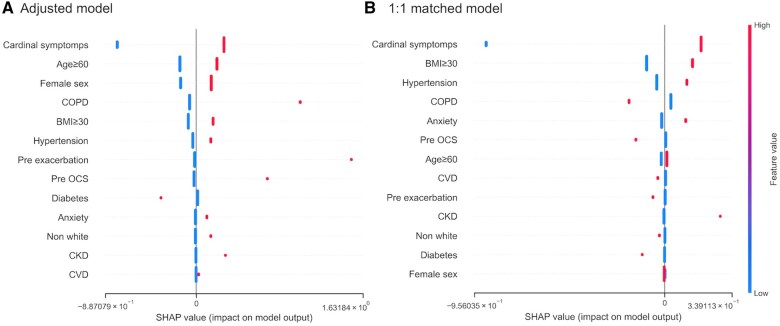
SHAP values of covariates in (A) adjusted and (B) 1:1 matched models.

SHAP values (Figure 3) showed that in the adjusted model, cardinal symptoms, age ≥ 60, and female sex had the strongest influence on prediction, whereas in the matched model, primary contributors were cardinal symptoms, BMI ≥ 30, and hypertension. In the adjusted model, diabetes had a negative SHAP value, suggesting an association with reduced outcome risk. In the matched model, the influence and absolute SHAP values of baseline covariates were markedly reduced with COPD and 1-year OCS Rx remaining influential with reversed effect directions.

### Observed variations in ORs under DP

**Figure 4.**
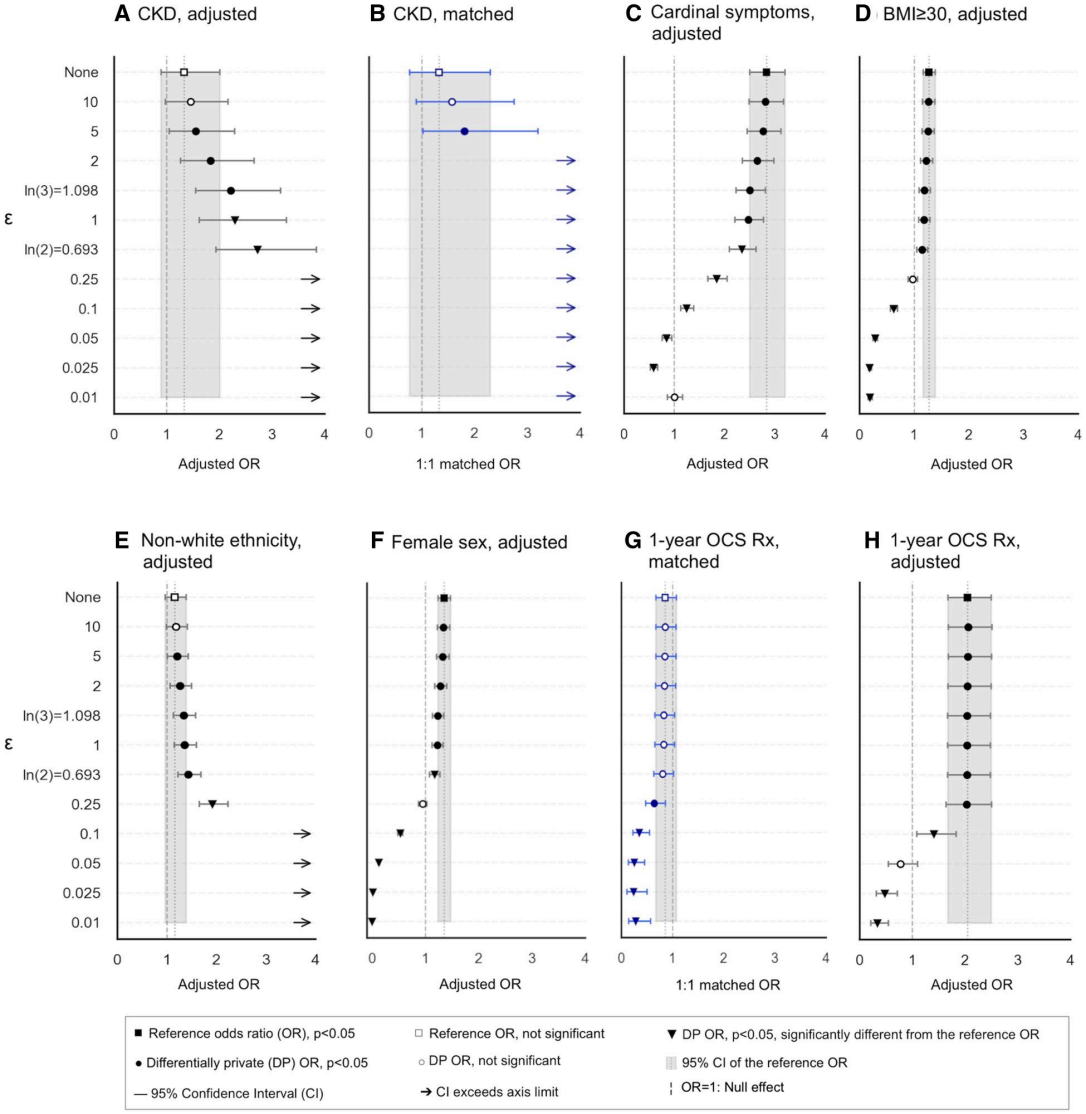
Selected forest plots of differentially private odds ratios and 95% confidence intervals across epsilon values compared with non-differentially private odds ratios: (A) Adjusted model for CKD; (B) One-to-one matched model for CKD; (C) Adjusted model for cardinal symptoms; (D) Adjusted model for BMI≥30; (E) Adjusted model for non-White ethnicity; (F) Adjusted model for female sex; (G) One-to-one matched model for 1-year OCS Rx; (H) Adjusted model for 1-year OCS Rx.

The impact of DP on ORs varied significantly based on epsilon, covariate frequency and balance, random seed, and propensity score calculations. Some effects of DP resemble those of exposure misclassification in case-control studies, where errors occur equally across groups (non-differential), typically biasing the OR toward the null, or differ between cases and controls (differential), potentially distorting the OR’s magnitude or direction, including shifts toward zero or infinity.^51^ Forest plots in Figure 4 illustrate these effects, with Tables SB3 and SB4 containing complete results.

#### Magnitude and directional changes in ORs

In adjusted models with a fixed random seed, high-prevalence covariates diverged at *ϵ <*ln(2) while rare covariates started fluctuating at higher epsilon values. For instance, the OR for CKD deviated from the reference CI at *ϵ=*ln(3). In matched models, rare covariates (eg, CKD) displayed heightened sensitivity to epsilon changes due to reduced sample sizes. For example, OR for CKD increased from 1.81 (1.02-3.21) at *ϵ=*5 to 5.32 (2.46-11.51) at *ϵ=*ln(3) and continued rising at smaller epsilon values.

#### Impact of random seeds on OR variability

Since DP relies on random noise, using different random seeds can impact the variability of outputs, especially in rare covariates. For instance, the 1-year history of OCS Rx deviated from the reference CI at *ϵ =* 0.1 and *ϵ=*5 with 2 different random seeds. Sensitivity analysis using 3 additional random seeds (Tables SB5-SB7) revealed substantial variations, especially for CKD, the rarest variable. Table 2 summarizes the ratio of covariate prevalence in cases and controls (prevalence ratios) for assessing covariate balance, and the epsilon ranges where OR began diverging from reference CIs across 4 random seeds.

**Table 2.** Ranges of epsilon where differentially private OR diverges from the reference OR in adjusted and matched studies.

	Adjusted study			1:1 Matched study		
Case-control size and ratio	**Cases: 2714, Controls: 19 451** **Case-to-control ratio: 1:7.2**			**Cases: 2714, Controls: 2713** **Case-to-control ratio: 1:1**		
**Covariate**	**Case-control** **prevalence ratio, 95% CI** ^a^	Epsilon of divergence	Range of epsilons of divergence	**Case-control** **prevalence ratio, 95% CI**	Epsilon of divergence	Range of epsilons of divergence
**Age ≥ 60 (matching covariate)**	1.29 (1.24-1.34)	[2-1.098]	0.90	1.01 (0.96-1.07)	[2-1.098]	0.90
**Female sex (matching covariate)**	1.10 (1.06-1.15)	[2-0.25]	1.75	1.01 (0.96-1.07)	[2-0.25]	1.75
**Non-White ethnicity (matching covariate)**	1.07 (1.03-1.11)	[2-0.25]	1.75	0.96 (0.91-1.01)	[2-0.25]	1.75
**Anxiety**	1.20 (1.15-1.25)	[2-0.25]	1.75	1.16 (1.10-1.22)	[2-0.25]	1.75
**BMI ≥ 30**	1.29 (1.24-1.34)	[1.098-0.25]	0.85	1.25 (1.19-1.32)	[2-0.25]	1.75
**CKD**	1.81 (1.74-1.88)	[5-1.098]	3.90	1.42 (1.35-1.50)	[10-2]	8.00
**COPD (matching covariate)**	3.35 (3.22-3.49)	[2-1.098]	0.90	0.98 (0.93-1.03)	[-1.098]	0.90
**CVD**	1.42 (1.36-1.48)	[2-1]	1.00	1.1 (1.04-1.16)	[2-1.098]	0.90
**Diabetes**	1.04 (1.00-1.08)	[5-0.693]	4.31	1.06 (1.01-1.12)	[5-1.098]	3.90
**Hypertension**	1.41 (1.35-1.47)	[1.098-0.693]	0.41	1.2 (1.14-1.27)	[1.098-0.25]	0.85
**Cardinal symptoms**	1.28 (1.23-1.33)	[1.098-1]	0.09	1.23 (1.17-1.30)	[1.098-0.05]	0.85
**1 year history of exacerbation events (matching covariate)**	5.76 (5.53-6.00)	[5-2]	3.00	1.08 (1.02-1.14)	[5-1.098]	3.90
**1 year history of OCS Rx (matching covariate)**	2.71 (2.60-2.82)	[5-0.1]	3.90	0.96 (0.91-1.01)	[5-2]	3.00

a The prevalence ratio is defined as the ratio of covariate prevalence in cases to that in controls, and 95% CIs were calculated using log-transformed standard errors.

#### Shifts of statistical significance

Most moderate changes in effect sizes resulted in statistically significant ORs that overlapped with the reference CI. However, in some cases, while the new OR remained statistically significant, its difference from the reference effect size was also statistically significant. For example, the adjusted OR for cardinal symptoms (Figure 4) dropped to 2.35 (2.10-2.63) at *ϵ=*ln(2), which is statistically significant yet falls outside the reference 95% CI of (2.51-3.21).

#### Directional and extreme changes in ORs

Another pattern emerged when DP led to larger or directional shifts in ORs. For instance, as epsilon decreased from ln(2) to 0.1, the adjusted OR for BMI ≥ 30 shifted from 1.15 (1.05-1.25) to 0.63 (0.57-0.70). Such changes can be extreme, with ORs inflating to implausible values or approaching zero. For example, at *ϵ<*0.05, the OR for non-White ethnicity exceeded 20, while the OR for female sex fell to zero.

#### Non-differential and false-positive bias effects

In some cases, DP resembled the effects of non-differential misclassification by shifting ORs toward the null hypothesis. For example, the reference adjusted OR for the female sex decreased from 1.35 (1.24-1.47, *P=*$1.81\times 10^{-11}$) to 0.95 (0.87-1.03, *P = .19*) at *ϵ=*0.25. DP occasionally introduced false-positive bias when reference ORs were not statistically significant, as minor noise levels rendered ORs significant. For instance, the adjusted OR for non-White ethnicity increased from 1.16 (0.97-1.39, *P = .09*) in the reference model to 1.20 (95% CI: 1.01-1.43, *P=*$3.42\times 10^{-4}$) at *ϵ=*5.

False-positive bias was particularly evident when a risk factor was used as a confounder in propensity score matching. For example, the matched reference OR for 1-year OCS Rx was 0.86 (0.68-1.08, *P = .19*), indicating a balance between cases and controls. However, at *ϵ=*0.25, the OR shifted slightly negative to 0.65 (0.48-0.87, *P=*$3.53\times 10^{-3}$), potentially resembling the effects of residual confounding.

### Overall model performance under DP

**Figure 5.**
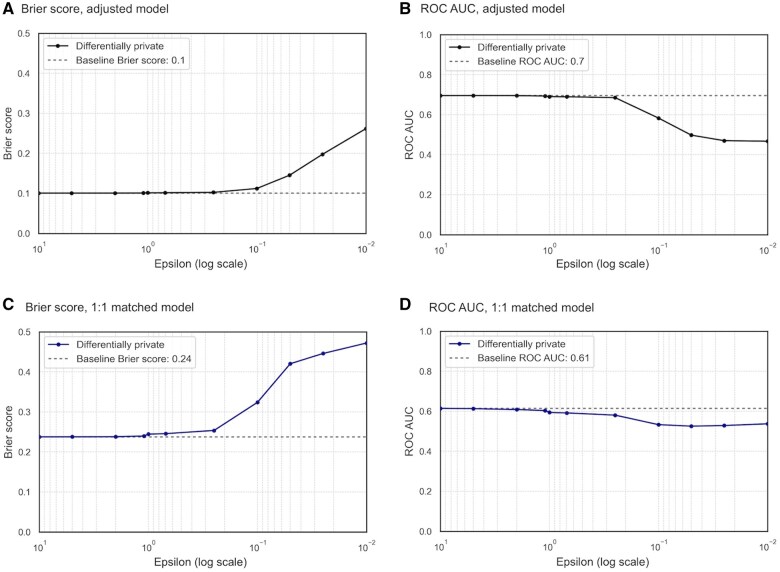
Brier Score and ROC AUC of adjusted and one-to-one matched models across epsilon values on a logarithmic scale: (A) Brier Score for adjusted model; (B) ROC AUC for adjusted model; (C) Brier Score for one-to-one matched model; (D) ROC AUC for one-to-one matched model.

Figure 5 presents the Brier score and ROC AUC plots for reference versus differentially private adjusted models across different epsilon values. The Brier score consistently increased after *ϵ=*0.25, indicating reduced probabilistic prediction accuracy as higher levels of noise were introduced. The ROC AUC declined at *ϵ=*0.25, indicating reduced model discrimination between cases and controls. At lower epsilon values, the model performance approached random guessing, with the ROC AUC approaching 0.5. In the matched study, the Brier score and ROC AUC deviated from the baseline values at higher epsilon values (*ϵ=*1), potentially due to a reduced sample size from matching.

### The effects of DP on variable importance

SHAP values (Table SB8) displayed variations in their direction, order, and magnitude as epsilon changed, with rare variables fluctuating at higher values (*ϵ=*1) and more prevalent variables remaining stable until lower values (*ϵ=*0.1). Below *ϵ=*0.1, variable importance fluctuated significantly, with some variables nearly reversing influence.

### Sensitivity analysis

The matched analyses with 1:2 and 1:4 ratios displayed slightly reduced covariate balance but showed similar DP-induced changes in OR magnitude and direction as in the primary analysis. We also applied DP to the propensity score calculation to assess its impact on matching performance. Covariate balance began to decline at *ϵ=*ln(2). Significant differences in continuous covariates, such as age and asthma duration, were observed even at *ϵ=*10. Binary covariates exhibited increasing variability below *ϵ=*ln(2).

Additional sensitivity analyses included unadjusted analyses based on contingency tables and exclusion of death events. Summary results for each sensitivity analysis are provided in Tables SB9-SB18.

## Discussion

Real-world observational studies on EHR data involve complexities arising from the interplay between data characteristics and study design, which influence the implementation and outcomes of DP. In this study, we empirically investigated the effects of DP on a case-control analysis of 1-year asthma exacerbation using EHR data from UK Biobank, highlighting its impact on effect sizes and model performance. Rather than prescribing an optimal epsilon, we illustrated how characteristics of multidimensional observational studies influence outcomes and the choice of epsilon in DP-enabled logistic regression. The observed impacts of DP were primarily influenced by matching, sample size, covariate prevalence, and variations across random seeds. Tools such as SHAP values, model calibration and discrimination metrics, and forest and balance plots proved valuable for interpreting the various aspects of the effects of DP.

### Various effects of DP in case-control studies

The matched study showed greater sensitivity to DP, with larger deviations from reference ORs at higher epsilon values, likely due to the reduced sample size. Case-control studies, particularly suitable for rare outcomes, often yield a limited number of cases.^52^ Smaller control-to-case ratios for matching further reduce the cohort size, requiring larger noise with lower epsilon values.^26^

Within adjusted and matched models, effect size variability was influenced by covariate prevalence and balance. Rare covariates (prevalence < 3%) exhibited greater fluctuations, deviating from reference ORs at higher epsilons (eg, *ϵ=*5), while high-prevalence covariates remained more stable. Some distortions in effect sizes resembled differential or non-differential misclassification.^51^

At mid-range epsilon values [0.25-ln(3)], ORs typically remained plausible (eg, OR < 5). However, with decreasing epsilon, ORs diverged significantly from reference values, occasionally reversing effect directions. At low epsilon values (*ϵ<*0.25), ORs occasionally inflated to unrealistic values or collapsed near zero. Covariates with weak associations were particularly vulnerable to these shifts, as minor changes drove ORs toward the null. Similarly, small noise levels (ie, large epsilon values) occasionally rendered non-significant ORs statistically significant, leading to false-positive bias. This pattern was similar to residual confounding and particularly affected covariates included in propensity scores.

As measured by the Brier score and ROC AUC, overall model performance declined as epsilon decreased, with sharp accuracy losses observed below *ϵ=*0.1. In the matched study, the effect of DP on model performance at higher epsilon values was likely influenced by the reduced sample size. Given the sensitivity of the Brier score to outcome prevalence,^53^ it is best suited for comparing epsilons within the same study rather than across different analyses (eg, adjusted vs matched).

### Random seed and the risk of *p*-hacking

DP effects varied across random seeds, with some producing unrealistically stable ORs even at lower epsilon values. Using predetermined random seeds or averaging results across multiple seeds may reduce the risk of *p*-hacking. In this study, epsilon values smaller than ln(2) consistently led to noticeable fluctuations across random seeds. While we utilized binary covariates for parsimony, incorporating mixed data types could further complicate the calculation of data sensitivity, influencing the magnitude of noise.

### Implications of determining the privacy budget in observational research

As the impact of DP on effect sizes is study-specific, experimenting with varying covariates, sensitivities, and matching ratios is essential for determining an optimal epsilon. Establishing a repository of observational case studies aligned with the Epsilon Registry proposed by Dwork^24^ could offer valuable guidance in determining the optimal context-specific privacy budgets.

The choice of epsilon also depends on the analysis pipeline and the total privacy budget. In real-world settings, datasets are often reused, raising critical questions about how many outputs can be safely released and how this influences the choice of epsilon. Releasing numerous high-accuracy private outputs increases the risk of inference, tracing, and reconstruction attacks.^24^^,^^54^ To mitigate this risk, it is essential to define and enforce a maximum privacy budget for the dataset.^24^

Observational studies often incorporate iterative queries, making privacy budget management challenging—an issue that remains largely underexplored. These queries involve handling missing values, detecting outliers, summary statistics, matching, and sensitivity analyses.^55–58^ Even with well-defined study protocols, real-world data often necessitate within-study covariate analyses to refine variable selection and reduce bias.^59^ For example, while boundaries for categorizing numerical variables may be predetermined, adjustments are often required to ensure adequate group sizes.^60^ Machine learning workflows further complicate the analysis pipeline with tasks such as handling class imbalance, cross-validation, and performance tuning, all of which generate additional outputs.

Optimizing epsilon and managing the privacy budget also depends on defining trust boundaries, which determine the extent of researchers’ access to microdata with no identifiable attributes (eg, names and addresses). Trust assumptions vary across governance models, requiring adaptive strategies for privacy budget management. We present 2 hypothetical data governance scenarios and their implications for allocating the privacy budget in observational studies.^5^ Both scenarios are based on the Five Safes principles and rely on a trust model that ensures transparency in research and balances data privacy with the best interests of patients and the public.^61^^,^^62^ In the first scenario, researchers do not have direct access to microdata, and the data holder performs analysis queries. DP is applied to intermediate outputs, including summary statistics, missing value exploration, outlier detection, iterative adjustments, and sensitivity analysis. Therefore, epsilon must be set lower to conserve the privacy budget. Comparing different epsilon values may also be constrained, as multiple outputs contribute to cumulative privacy loss.

In the second scenario, researchers operate within the trust boundary of an SDE, with direct access to microdata with no identifiable attributes. This setup allows for high-precision data utilization throughout the pipeline, reserving DP for outputs intended for public reporting. Limiting DP to key outputs reduces total privacy expenditure, enabling moderate epsilon values that balance research accuracy with privacy preservation.

### Key findings, considerations, and future research

By examining adjusted and matched models through a wide range of evaluation methods, our study illustrates specific, quantifiable patterns in the effects of DP on a case-control analysis. While the findings highlight the potential utility of DP in case-control studies, they also demonstrate covariate-dependent variability in the stability of differentially private ORs. Prevalent and balanced variables exhibited relatively greater stability at mid-range epsilon values and higher compared to rare covariates. At lower epsilon values, the patterns of DP effects depend on multiple factors, requiring further exploration in other cohorts and observational study designs. Beyond identifying these patterns, our analysis highlights key considerations for study design, epsilon selection, and result interpretation. In particular, it underscores the importance of factors such as sample size (influenced by matching ratio), overall privacy budget, seed randomization, and trust models, all of which are pivotal for practical DP implementations. These insights provide a foundation for key methodological considerations in the application of DP to observational studies.

The first key consideration is how sample size influences DP-induced noise. Case-control studies often involve rare outcomes, limiting the number of cases. Matching with lower control-to-case ratios further reduces sample size, exacerbating the impact of DP. Evaluating robustness across epsilon values, matching ratios, and covariates can help mitigate these effects. Second, DP may introduce variability in risk estimates for rare variables, requiring careful assessment of whether observed effects reflect actual associations or DP-induced distortions. This is particularly relevant when evaluating confounders used in propensity score matching and potential changes in effect direction or statistical significance. Sensitivity analyses incorporating alternative adjustment and matching strategies can help assess the extent of DP-related distortions. Third, the inherent randomness of DP mechanisms may result in artificially optimistic results. To mitigate the risk of *p*-hacking, random seeds should be predefined in the study protocol or results averaged across multiple seeds. Finally, limiting the number of intermediate outputs by carefully structuring queries or providing trusted researchers with access to non-DP microdata in SDEs can help preserve the overall privacy budget.

Our analysis is limited to a subset of the UK Biobank participants with linked GP data, which may not fully represent the general population, limiting the generalizability of the findings. Consequently, the clinical relevance of the case-control analysis is specific to this cohort. We restricted our analysis to binary and dichotomized covariates for parsimony; therefore, the effects of data sensitivities with mixed data types were not examined. Furthermore, conclusions about optimal epsilon values, their impact on effect sizes, and the broader applicability of DP in case-control studies should be interpreted within the context of this study and the implementation of DP logistic regression using diffprivlib.

Further research is needed to better understand the implications of DP in multidimensional observational studies, particularly its effects on rare exposures and outcomes across different datasets and study designs. Synthetic data can support the evaluation of reproducible case studies and the development of DP-based software packages, such as Poisson regression for cohort studies and Cox regression for survival analysis.^14^^,^^36^^,^^63^ Additionally, applying DP-enabled federated learning to large-scale epidemiological research remains an open area of study that warrants further investigation.^64^

## Conclusion

Rather than prescribing an optimal epsilon value for case-control studies, we demonstrated how variations in epsilon influence adjusted and matched analyses. Additionally, we highlighted other factors influencing epsilon selection, including random seed variability and trust models, which play a critical role in preserving the privacy budget. The selection of an optimal epsilon value is context-dependent and varies across studies.

## Supplementary Material

ocaf090_Supplementary_Data

## Data Availability

The data used in this study are available to registered researchers through an application to UK Biobank. The code is available in the following GitHub repository below: github.com/mehrdadmzn/Observational-DP-Effects
