# Supplementary material for: An empirical assessment of differential privacy in real-world observational data: a case-control study of asthma exacerbation in UK Biobank linked with electronic health records
Source: J Am Med Inform Assoc. 2025 Jun 18;32(8):1328–39. doi: 10.1093/jamia/ocaf090 (PMC12277706; doi:10.1093/jamia/ocaf090)
Supplement: ocaf090_Supplementary_Data [file ocaf090_supplementary_data.zip › ocaf090_Supplementary_Data/Supplementary_A_SA.docx]

# Supplementary materials A (SA)

# **List of supplementary tables**

[Table SA 1. Specifications of the literature review. 2](#_Toc184749753)

[Table SA 2. Results of the literature review. 2](#_Toc184749754)

[Table SA 3. Phenotyping methodology. 4](#_Toc184749755)

[Table SA 4. List of all variables used in the primary and sensitivity analyses. 5](#_Toc184749756)

[Table SA 5. Candidate models for covariate selection.^1^ 9](#_Toc184749757)

[Table SA 6. Feature importance in candidate logistic regression models. 10](#_Toc184749758)

[Table SA 7. Log-likelihood test results for candidate logistic regression models. 11](#_Toc184749759)

Table SA 1. Specifications of the literature review.

| **Sources** | PubMed, ACM Digital Library, JAMIA, IEEE Xplore, Arxiv |
| --- | --- |
| **Search terms** | “differential privacy” AND “review”  “differential privacy” AND “epidemiology” |
| **Date** | Any |
| **Study type** | Any |
| **Inclusion criteria** | - Studies related to observational studies (including case-control and cohort studies) or regression analysis (as related to adjusted analytical studies), either in central or federated analysis/averaging settings. - Data must be related to tabular electronic health records or collected in clinical trials with large number of patients (>1000) and with heterogeneous covariates such as comorbidities. - Empirical studies on the effect of differential privacy and epsilon levels on epidemiological studies. |
| **Exclusion criteria** | - Non-tabular data (e.g., image, free text). - Non-empirical and theoretical-only studies. - Focused exclusively on local differential privacy, data publishing, synthetic data generation, or predictive analysis (e.g. machine learning only). - Non-observational studies. - Not related to the effects of differential privacy and epsilon levels on epidemiological studies. |

Table SA 2. Results of the literature review.

| **Source** | **Search details** | **Number of articles** | **Search results and Selected articles** |
| --- | --- | --- | --- |
| PubMed | “Differential privacy” AND “Review”  No time limit  No study type filtering | 121 | Only one review included the empirical aspects of differential privacy in observational studies.  - (Ficek, et al. 2021)^1^: The most relevant scoping review, which includes articles on the development and usage of differential privacy in health research, and highlights the gap in this area and the need for more case studies. |
| Article  (Ficek, 2021) | - | 54 | The main scoping review relates to the topic of our research. The authors have reviewed 54 selected articles up to and including 2021. Most of the reviewed studies focus on predictive modelling and data release. Only one paper is related to epidemiology; however, its main topic is survival analysis rather than analytical observational studies. |
| PubMed | “differential privacy” And “epidemiology”  No time limit  No study type filtering | 67 | Most articles focus on data sharing, statistical/census aggregate results sharing, Non-electronic health records (e.g., mobile data and imaging), or predictive modelling. We found no studies on the effects of differential privacy and epsilon levels on analytical observational studies, including adjusted and matched studies.  - (Sailek, et al., 2021)^2^: This study compares several metrics, including area under the curve and odds ratios in central vs federated learning models based on TensorFlow. Reviewed case-control and cohort studies are limited in terms of either number of patients or covariates. The only study with co-morbidities included as covariates is limited to 159 patients.  - (Ji, et al., 2014)^3^: Compares the logistic regression models in public data vs distributed logistic regression on private data. The main aim is to compare different models under different privacy parameters in terms of the area under the curve rather than analytical observational studies. |
| JAMIA | “differential privacy” And “epidemiology”  No time limit  No study type filtering | 83 | - (Chen, et al., 2024)^4^: This study assesses the effects of differential privacy on data sharing in clinical trials. The authors present the results of differential privacy with various levels of epsilon on two clinical trial studies with 2780 and 451 patients respectively. The focus is on data sharing and only the main odds ratios of the main exposure is presented.  - (Bonomi, et al., 2020)^5^: Presenting a framework for differentially private survival analysis. |
| ACM Digital Library | “differential privacy” And “epidemiology”  No time limit  No study type filtering | 1169 | - (Zhao, et al., 2024)^6^: This survey presents a technical summary of local and global differential privacy adaptations. It also presents the correlation between data characteristics and the specification of differential privacy. It does not include any empirical examples of analytical observational studies.  - (Ficek, et al., 2021)^1^: A reference in (Zhao, 2024), presents a technical survey of the literature on differentially private logistic regression.  **-** (Danker, et al., 2012)^7^: This paper presents a general review of differential privacy and its limitations, proposed solutions, and challenges of its application in health data. It does not include any empirical results on analytical observational studies. |
| IEEE Xplore | “differential privacy” And “epidemiology”  No time limit  No study type filtering | 14 | No studies related to empirical or theoretical aspects of differential privacy in analytical observational studies. |
| Arxiv |  | 4 | No studies related to empirical or theoretical aspects of differential privacy in analytical observational studies. |

^1^Ficek J, Wang W, Chen H, et al. A Survey of differentially private regression for clinical and epidemiological research. *Int Stat Rev* 2021;89(1):132–47.

^2^Sadilek A, Liu L, Nguyen D, Kamruzzaman M, et al. Privacy-first health research with federated learning. *NPJ Digit Med* 2021;4(1):132.

^3^Ji Z, Jiang X, Wang S, et al. Differentially private distributed logistic regression using private and public data. *BMC Med Genomics* 2014;7:1-10.

^4^Chen H, Pang J, Zhao Y, et al. A data-driven approach to choosing privacy parameters for clinical trial data sharing under differential privacy. *J Am Med Inform Assoc* 2024;31(5):1135-43.

^5^Bonomi L, Jiang X, Ohno-Machado L. Protecting patient privacy in survival analyses. *J Am Med Inform Assoc* 2020;27(3):366–75.

^6^Zhao Y, Du JT, Chen J. Scenario-based adaptations of differential privacy: a technical survey. *ACM Comput Surv* 2024;56(8):1–39.

^7^Dankar FK, El Emam K. The application of differential privacy to health data. In: *Proceedings of the 2012 Joint EDBT/ICDT Workshops* 2012;2012:158–66.

Table SA 3. Phenotyping methodology.

| **Step** | **Process** | **Details** |
| --- | --- | --- |
| 1 | Map Read v2 to CTV3 in code-lists | GP records contain both Read v2 and CTV3 codes. Map Read v2 in code-lists to any different CTV3 code based on the mapping in UK Biobank resource 592: <https://biobank.ndph.ox.ac.uk/showcase/refer.cgi?id=592>. |
| 2 | Map SNOMED CT to Read v2 and CTV3 in code-lists | Map asthma exacerbation codes in SNOMED CT to Read v2 and CTV3 using HDR UK phenotype library based on the description of the code. The source for the SNOMED CT codes is Mukherjee 2024: <https://doi.org/10.1016/j.lanepe.2024.100938> |
| 3 | MAP dm+d codes to BNF codes | Prescribed medications are coded in Read v2, dm+d, and BNF codes. Map the dm+d codes in Mukherjee 2024 to BNF codes using <https://www.nhsbsa.nhs.uk/prescription-data/understanding-our-data/bnf-snomed-mapping> |
| 4 | MAP BNF codes to Read v2 codes | Prescribed medications are coded both in Read v2, dm+d, and BNF codes. Map the BNF codes from the previous step into Read v2 codes using <https://biobank.ndph.ox.ac.uk/showcase/refer.cgi?id=592>. |
| 5 | Diagnostic phenotyping | Extract all the unique event dates per person in GP records (based on Read v2 and CTV3) and HES APC (based on ICD-10) |
| 6 | Prescribed medication dm+d and Read v2 | Simple matching |
| 7 | Prescribed medication BNF | The UK Biobank prescription data utilizes BNF codes of varying lengths. Shorter codes represent broader categories of medications, while longer codes identify specific drugs (<https://www.bennett.ox.ac.uk/blog/2017/04/prescribing-data-bnf-codes/>) . We followed a two-step mapping process for shorter codes:   1. **Word-based Matching**: We mapped medications based on the initial words of the drug description. 2. **Semantic Similarity**: We utilized the all-MiniLM-L6-v2 model, a pre-trained Sentence Transformer, to compute semantic similarity between drug descriptions, especially when dealing with broader BNF codes that specify chemical substances rather than individual drugs. The all-MiniLM-L6-v2 model, available at <https://sbert.net/>, is designed for assessing text similarities. |
| 8 | Non-repeat medication | Repeat medications in the UK are typically prescribed in 28- or up to 56-day intervals for stable conditions and non-controlled drugs^1^. There is no specific flag in the UK Biobank prescription data to indicate repeat prescription. To identify repeat prescription, we employed a stringent criterion, considering 3 consecutive medication prescriptions within 56-days intervals as an indication of a repeat prescription. |
| 9 | Asthma exacerbation | The definition of asthma exacerbation is adapted from (Mukherjee et al., 2024)^2^ with the following modification for the UK Biobank data:   1. Asthma exacerbation: SNOMED CT codes were mapped to Read v2 and CTV3 code used in UK Biobank GP records. 2. Prescription of prednisolone, excluding repeat prescription: The dmd+d codes were mapped into Read v2, and BNF codes to cover different coding systems in UK Biobank. 3. Asthma-related hospitalization: To avoid missing any exacerbation cases, we included all asthma-related hospitalization, as asthma exacerbation. 4. Accident and emergency attendance: We used any Read v2 codes in GP records indicating recent A&E attendance related to asthma. |

^1^Khan T, Jackson A. Prescription duration guidance. Prescribing, Policies and Pathways. Hertfordshire and West Essex ICB 2023.

^2^Mukherjee M, Okusi C, Jamie G, et al. Modifiable risk factors for asthma exacerbations during the COVID-19 pandemic: a population-based repeated cross-sectional study using the Research and Surveillance Centre primary care database. *Lancet Reg Health Eur* 2024;42:100938.

Table SA 4. List of all variables used in the primary and sensitivity analyses.

| **Variable type** | **Variable** | **Details/links** | **Clinical coding in the code-list** | **Clinical coding used in UK Biobank** | **Source** |
| --- | --- | --- | --- | --- | --- |
| Asthma exacerbation | Diagnosis at GP | Diagnostic codes in Read v2 and CTV3  <https://github.com/mehrdadmzn/Observational-DP-Effects/blob/main/phenotyping/asthma/Exacerbation_GP.csv> | SNOME CT | Read v2  CTV3 | Mukherjee, et al., 2024^1^ |
| Asthma exacerbation | Hospitalization | Inpatient admission with asthma  <https://github.com/mehrdadmzn/Observational-DP-Effects/blob/main/phenotyping/asthma/Exacerbation_HESAPC.csv> | SNOME CT | ICD-10 | Mukherjee, et al., 2024 |
| Asthma exacerbation | Indication of accident emergency in GP records | Accident and emergency (A&E) hospital data was not available for this research. We used any codes in GP records which indicated any (A&E) admission with asthma.  <https://github.com/mehrdadmzn/Observational-DP-Effects/blob/main/phenotyping/asthma/Exacerbation_accident_emergency_recorded_in_GP.csv> | SNOME CT | Read v2 | Mukherjee, et al., 2024 |
| Asthma exacerbation | Non-repeat OCS | Non-repeat prescription of oral corticosteroid-Prednisolone (OCS)  A prescription is marked as 'non-repeat' if there are no instances of three consecutive Oral Corticosteroid (OCS) prescriptions issued within 56 days of each other.^2^ The absence of closely spaced prescriptions is used as an indication that the OCS prescription is ‘non-repeat’.  <https://github.com/mehrdadmzn/Observational-DP-Effects/blob/main/phenotyping/medication/medication_ocs.csv> | dm+d | BNF  dm+d | Mukherjee, et al., 2024 |
| Prescribed medication | ICS | Inhaled corticosteroid  <https://github.com/mehrdadmzn/Observational-DP-Effects/blob/main/phenotyping/medication/medicaiton_ics.csv> | dm+d | BNF  dm+d | Mukherjee, et al., 2024 |
| Prescribed medication | OCS | Oral corticosteroid-Prednisolone (OCS)  <https://github.com/mehrdadmzn/Observational-DP-Effects/blob/main/phenotyping/medication/medication_ocs.csv> | dm+d | BNF  dm+d | Mukherjee, et al., 2024 |
| Prescribed medication | SABA_LABA | Short-Acting Beta Agonist, Long-Acting Beta Agonist or Beta2AdrenoceptorAgonistsSelective  <https://github.com/mehrdadmzn/Observational-DP-Effects/blob/main/phenotyping/medication/medication_saba_laba.csv> | dm+d | BNF  dm+d | Mukherjee, et al., 2024 |
| Prescribed medication | SAMA_LAMA | Short-Acting Muscarinic Antagonist, Long-Acting Muscarinic Antagonist or Antimuscarinics  <https://github.com/mehrdadmzn/Observational-DP-Effects/blob/main/phenotyping/medication/medicaiton_sama_lama.csv> | dm+d | BNF  dm+d | Mukherjee, et al., 2024 |
| Comorbidity | AAA | Abdominal aortic aneurysm  <https://github.com/mehrdadmzn/Observational-DP-Effects/blob/main/phenotyping/comorbidities/aaa.csv> | ICD-10  Read v2 | ICD-10  Read v2  CTV3 | <https://phenotypes.healthdatagateway.org/phenotypes/PH34/version/68/detail/> |
| Comorbidity | AMI | Acute myocardial infarction  <https://github.com/mehrdadmzn/Observational-DP-Effects/blob/main/phenotyping/comorbidities/ami.csv> | ICD-10  Read v2  SNOMED CT | ICD-10  Read v2  CTV3 | <https://phenotypes.healthdatagateway.org/phenotypes/PH949/version/2127/detail/> |
| Comorbidity | Anxiety | <https://github.com/mehrdadmzn/Observational-DP-Effects/blob/main/phenotyping/comorbidities/anxiety.csv> | ICD-10  Read v2 | ICD-10  Read v2  CTV3 | <https://phenotypes.healthdatagateway.org/phenotypes/PH1113/version/2453/detail/> |
| Comorbidity | Asthma | Used to determine asthma prevalence at baseline  <https://github.com/mehrdadmzn/Observational-DP-Effects/blob/main/phenotyping/asthma/asthma.csv> | ICD-10  ICD-11  Read v2  SNOMED CT | ICD-10  Read v2  CTV3 | <https://phenotypes.healthdatagateway.org/phenotypes/PH12/version/24/detail/> |
| Comorbidity | AF | Atrial fibrillation  <https://github.com/mehrdadmzn/Observational-DP-Effects/blob/main/phenotyping/comorbidities/af.csv> | ICD-10  Med codes  Read v2 | ICD-10  Read v2  CTV3 | <https://phenotypes.healthdatagateway.org/phenotypes/PH36/version/72/detail/> |
| Comorbidity | Cardiomyopathy | <https://github.com/mehrdadmzn/Observational-DP-Effects/blob/main/phenotyping/comorbidities/cardiomyopathy.csv> | ICD-10  Read v2  SNOMED CT | ICD-10  Read v2  CTV3 | <https://phenotypes.healthdatagateway.org/phenotypes/PH961/version/2139/detail/> |
| Comorbidity | CKD | Chronic Kidney Disease  <https://github.com/mehrdadmzn/Observational-DP-Effects/blob/main/phenotyping/comorbidities/ckd.csv> | ICD-10  Read v2  SNOMED CT | ICD-10  Read v2  CTV3 | <https://www.thelancet.com/journals/ebiom/article/PIIS2352-3964(23)00054-3/fulltext> |
| Comorbidity | COPD | Chronic obstructive pulmonary disease  <https://github.com/mehrdadmzn/Observational-DP-Effects/blob/main/phenotyping/comorbidities/copd.csv> | ICD-10  Read v2  SNOMED CT | ICD-10  Read v2  CTV3 | <https://doi.org/10.1016/j.ebiom.2023.104489> |
| Comorbidity | Depression | <https://github.com/mehrdadmzn/Observational-DP-Effects/blob/main/phenotyping/comorbidities/depression.csv> | ICD-10  Read v2  SNOMED CT | ICD-10  Read v2  CTV3 | <https://phenotypes.healthdatagateway.org/phenotypes/PH964/version/2142/detail/> |
| Comorbidity | Diabetes | Type 1, Type2, Other or uncertain types  <https://github.com/mehrdadmzn/Observational-DP-Effects/blob/main/phenotyping/comorbidities/diabetes.csv> | ICD-10  Med codes  Read v2 | ICD-10  Read v2  CTV3 | <https://phenotypes.healthdatagateway.org/phenotypes/PH152/version/304/detail/> |
| Comorbidity | DVT | Deep vein thrombosis, intracranial venous thrombosis  <https://github.com/mehrdadmzn/Observational-DP-Effects/blob/main/phenotyping/comorbidities/dvt.csv> | ICD-10  Read v2  SNOMED CT | ICD-10  Read v2  CTV3 | <https://phenotypes.healthdatagateway.org/phenotypes/PH966/version/2144/detail/> |
| Comorbidity | HF | Hear Failure  <https://github.com/mehrdadmzn/Observational-DP-Effects/blob/main/phenotyping/comorbidities/hf.csv> | ICD-10  Read v2  SNOMED CT | ICD-10  Read v2  CTV3 | <https://phenotypes.healthdatagateway.org/phenotypes/PH968/version/2146/detail/> |
| Comorbidity | HT | Hypertension  <https://github.com/mehrdadmzn/Observational-DP-Effects/blob/main/phenotyping/comorbidities/ht.csv> | BNF codes  ICD-10  Read v2  SNOMED CT | ICD-10  Read v2  CTV3 | <https://phenotypes.healthdatagateway.org/phenotypes/PH970/version/2148/detail/> |
| Comorbidity | PAD | Peripheral arterial disease  <https://github.com/mehrdadmzn/Observational-DP-Effects/blob/main/phenotyping/comorbidities/pad.csv> | ICD-10  Med codes  OPCS4  Read v2 | ICD-10  Read v2  CTV3 | <https://phenotypes.healthdatagateway.org/phenotypes/PH236/version/472/detail/> |
| Comorbidity | PE | Pulmonary embolism  <https://github.com/mehrdadmzn/Observational-DP-Effects/blob/main/phenotyping/comorbidities/pe.csv> | ICD-10  Read v2  SNOMED CT | ICD-10  Read v2  CTV3 | <https://phenotypes.healthdatagateway.org/phenotypes/PH978/version/2156/detail/> |
| Comorbidity | Stroke | <https://github.com/mehrdadmzn/Observational-DP-Effects/blob/main/phenotyping/comorbidities/stroke.csv> | ICD-10  Read v2  SNOMED CT | ICD-10  Read v2  CTV3 | <https://phenotypes.healthdatagateway.org/phenotypes/PH983/version/2161/detail/> |
| Comorbidity | Stroke NOS | Non-specified | ICD-10  Med codes  Read v2 | ICD-10  Read v2  CTV3 | <https://phenotypes.healthdatagateway.org/phenotypes/PH85/version/170/detail/> |
| UK Biobank, smoking | Smoking | Field: 20116 | - | - | <https://biobank.ndph.ox.ac.uk/showcase/field.cgi?id=20116> |
| UK Biobank | Date of attendance (baseline) | Field: 53 | - | - | <https://biobank.ndph.ox.ac.uk/showcase/field.cgi?id=53> |
| UK Biobank, sociodemographic | Date of birth | Year of birth from the field 22200.  Month of birth from field 52.  The day of birth is set to 01. | - | - | <https://biobank.ndph.ox.ac.uk/showcase/field.cgi?id=22200>  <https://biobank.ndph.ox.ac.uk/showcase/field.cgi?id=52> |
| UK Biobank, sociodemographic | Death | Field: 40000  National Death Registries, resource 115559 from data portal | - | - | <https://biobank.ndph.ox.ac.uk/showcase/field.cgi?id=40000>  <https://biobank.ndph.ox.ac.uk/showcase/refer.cgi?id=115559> |
| UK Biobank, sociodemographic | Sex | Field: 31 | - | - | <https://biobank.ndph.ox.ac.uk/showcase/field.cgi?id=31> |
| UK Biobank, sociodemographic | Ethnicity | Field: 21000 | - | - | <https://biobank.ndph.ox.ac.uk/showcase/field.cgi?id=21000> |
| UK Biobank, sociodemographic | Index of multiple deprivation (IMD) | England field: 26410  Scotland field: 26427  Wales field: 26426 | - | - | <https://biobank.ndph.ox.ac.uk/showcase/field.cgi?id=26410>  <https://biobank.ndph.ox.ac.uk/showcase/field.cgi?id=26427>  <https://biobank.ndph.ox.ac.uk/showcase/field.cgi?id=26426> |
| UK Biobank, linked health records | GP records from data portal | Category: 3000 | - | - | <https://biobank.ndph.ox.ac.uk/showcase/label.cgi?id=3000> |
| UK Biobank-linked health records | Hospital inpatient from data portal | Category: 2000 | - | - | <https://biobank.ndph.ox.ac.uk/showcase/label.cgi?id=2000> |
| UK Biobank, measurement | Body Mass Index (BMI) | Field: 21001 | - | - | <https://biobank.ndph.ox.ac.uk/showcase/field.cgi?id=21001> |
| UK Biobank, medical condition | Chest pain | Field: 2335 | - | - | <https://biobank.ndph.ox.ac.uk/showcase/field.cgi?id=2335> |
| UK Biobank, medical condition | Shortness of breath | Field: 4717 | - | - | <https://biobank.ndph.ox.ac.uk/showcase/field.cgi?id=4717> |
| UK Biobank, medical condition | Wheeze or whistling in the chest | Field: 2316 | - | - | <https://biobank.ndph.ox.ac.uk/showcase/field.cgi?id=2316> |
| UK Biobank, environmental factors | Living close to major road | Field: 24014 | - | - | <https://biobank.ndph.ox.ac.uk/showcase/field.cgi?id=24014> |

^1^Mukherjee M, Okusi C, Jamie G, et al. Modifiable risk factors for asthma exacerbations during the COVID-19 pandemic: a population-based repeated cross-sectional study using the Research and Surveillance Centre primary care database. *Lancet Reg Health Eur* 2024;42:100938.

^2^Khan T, Jackson A. Prescription duration guidance. Prescribing, Policies and Pathways. Hertfordshire and West Essex ICB 2023.

Table SA 5. Candidate models for covariate selection.^1^

|  | Binary covariates | | | | | | | | | | | | | | | | | |
| --- | --- | --- | --- | --- | --- | --- | --- | --- | --- | --- | --- | --- | --- | --- | --- | --- | --- | --- |
| Model name | Female | Age 60+ | Late onset >40 | Non-White | Cardinal Symptoms | Anxiety | BMI30+ | CKD | COPD | CVD | Depression | DM | HT | Pre-exacerbation | Pre-OCS | Pre-meds | Current smoker | Near major rad |
| Full | ● | ● | ● | ● | ● | ● | ● | ● | ● | ● | ● | ● | ● | ● | ● | ● | ● | ● |
| Near full | ● | ● | ● | ● | ● | ● | ● | ● | ● | ● | ● | ● | ● | ● | ● | ● | ● | - |
| Partial | ● | ● | ● | ● | ● | ● | ● | ● | ● | ● | ● | ● | ● | ● | ● | - | ● | - |
| Simple | ● | ● | - | ● | ● | ● | ● | - | ● | - | - | ● | ● | ● | ● | - | - | - |
| Basic | ● | ● | - | ● | ● | ● | ● | - | ● | - | - | ● | ● | ● | ● | - | - | - |
| Minimal | ● | ● | - | - | ● | - | ● |  | ● | - | - | - | - | - | - | - | - | - |

^1^A sensitivity analysis is conducted to identify the most important variables for clinical relevance, feature importance, log-likelihood tests, and parsimony.

Table SA 6. Feature importance in candidate logistic regression models.

| **Full model** | **Near full model** | **Partial model** |
| --- | --- | --- |
| 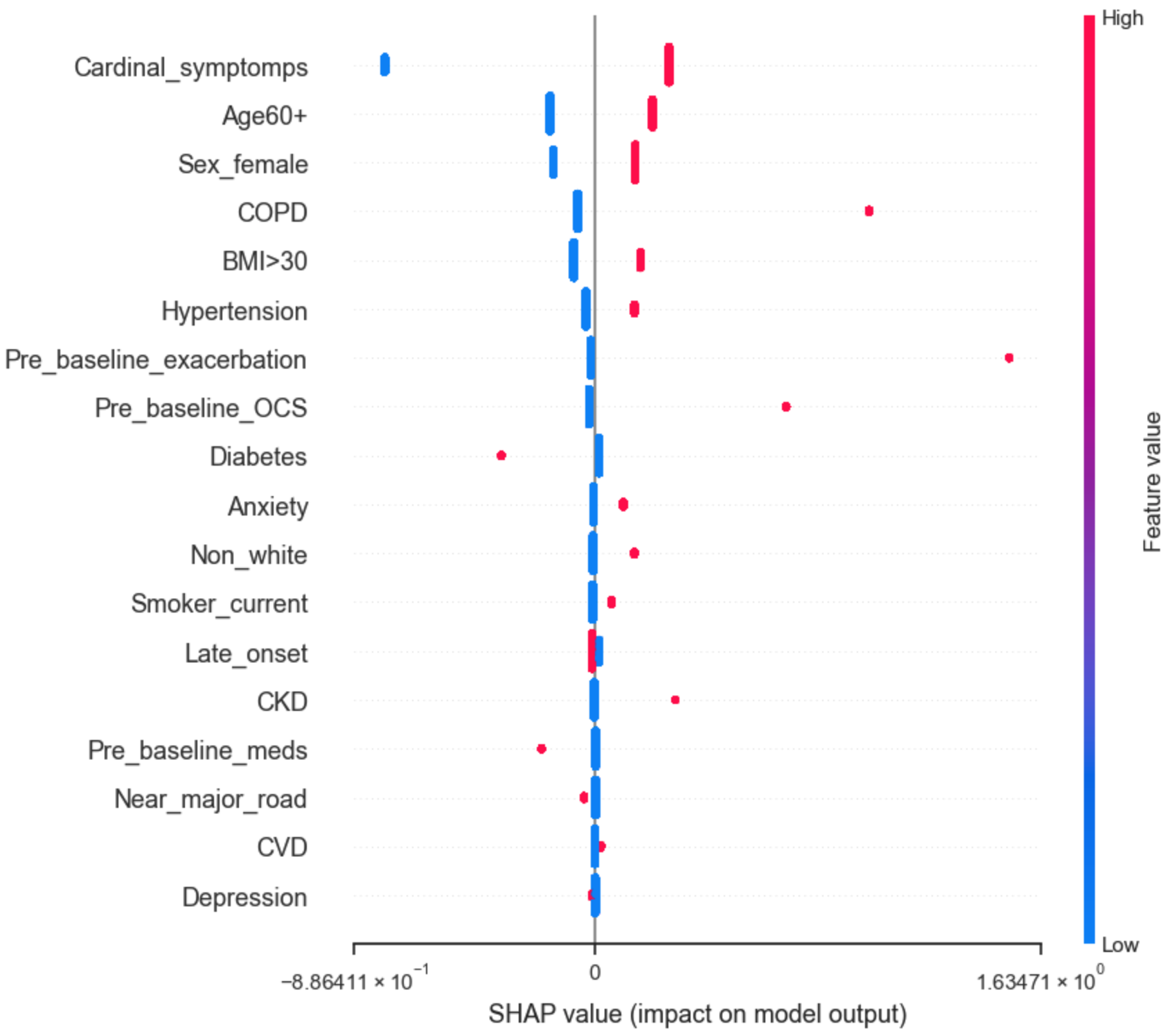 | 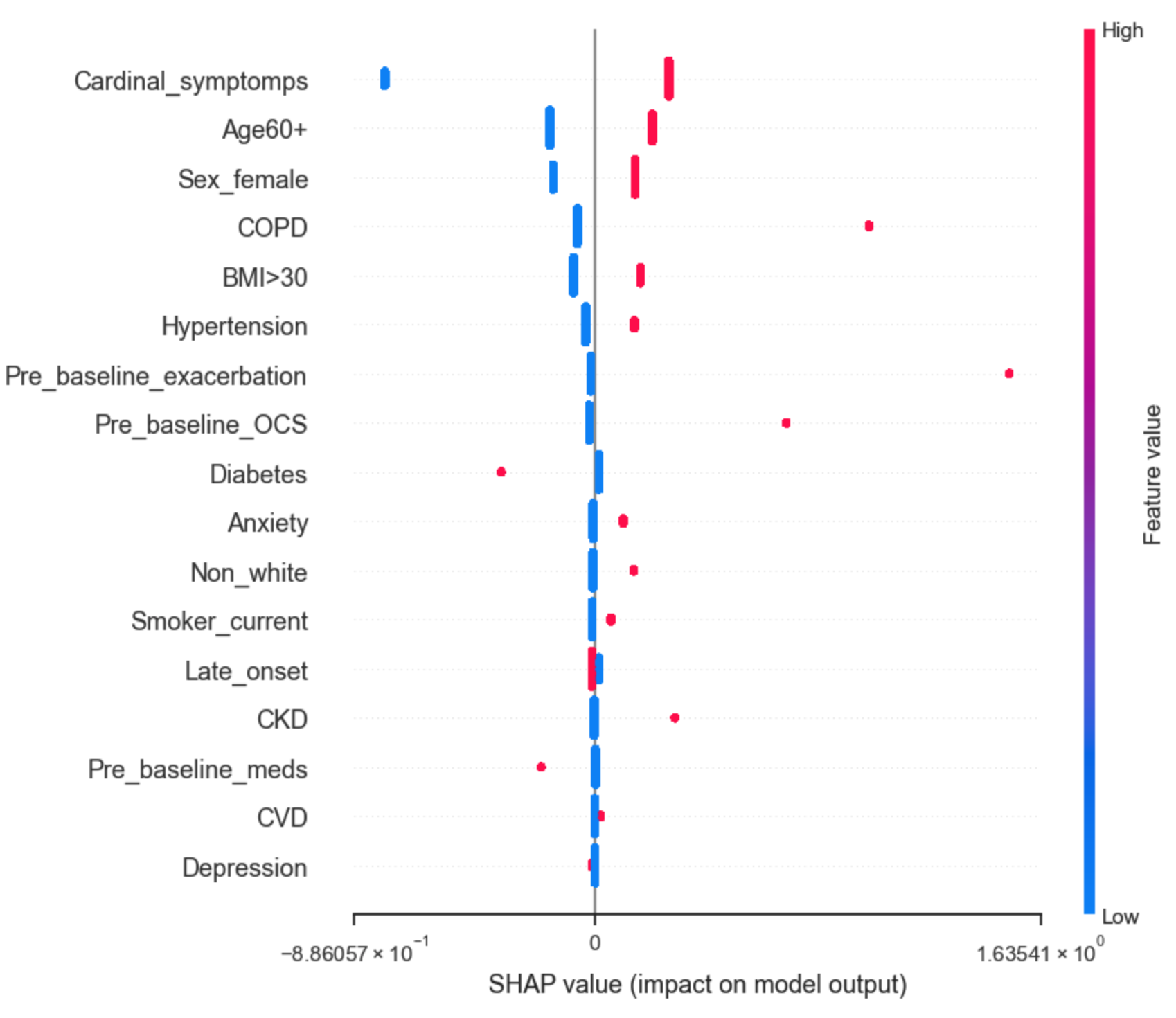 | 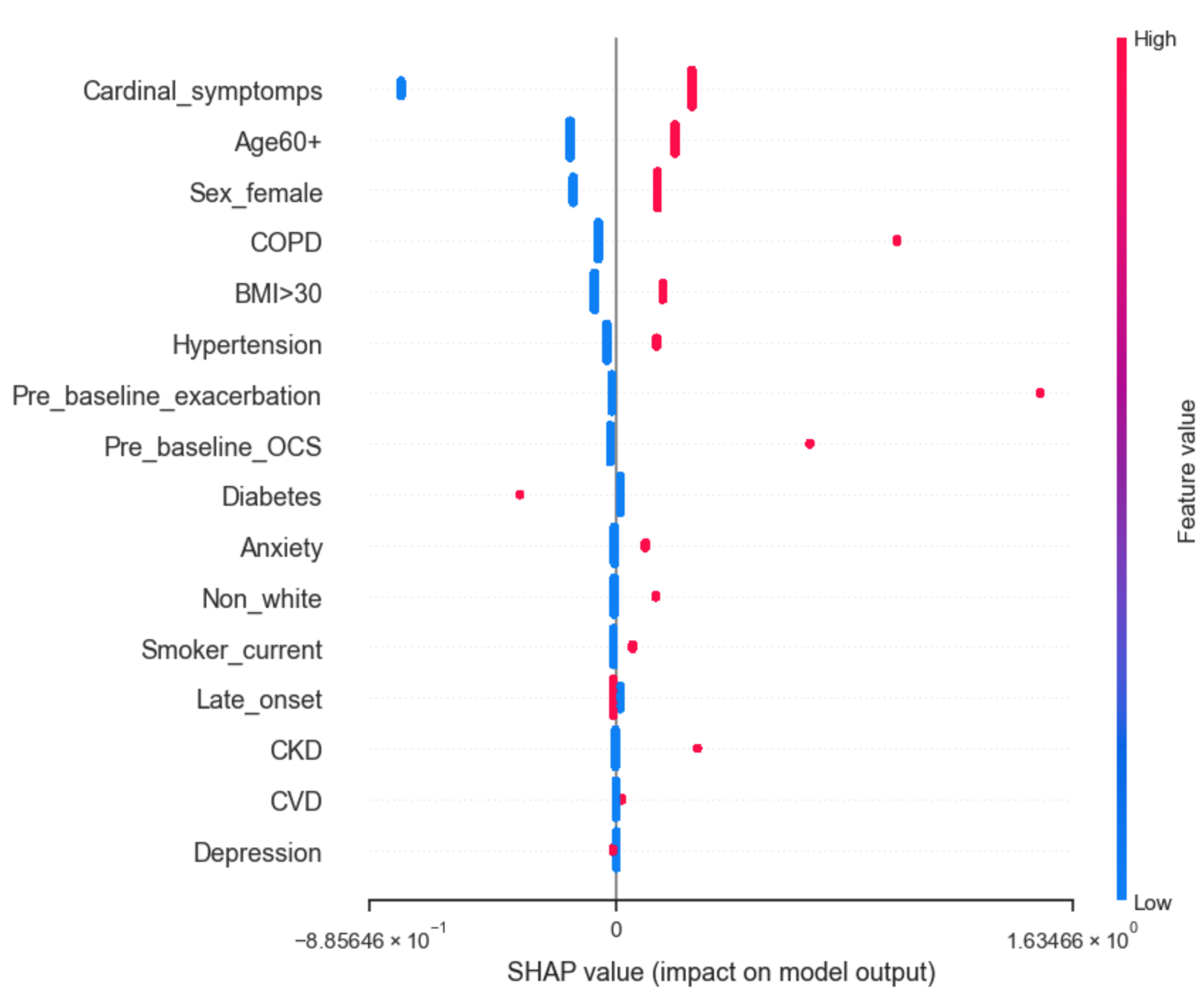 |
| Simple model | Basic model (top 5 SHAP values) | Minimal model (top 5 SHAP values) |
| 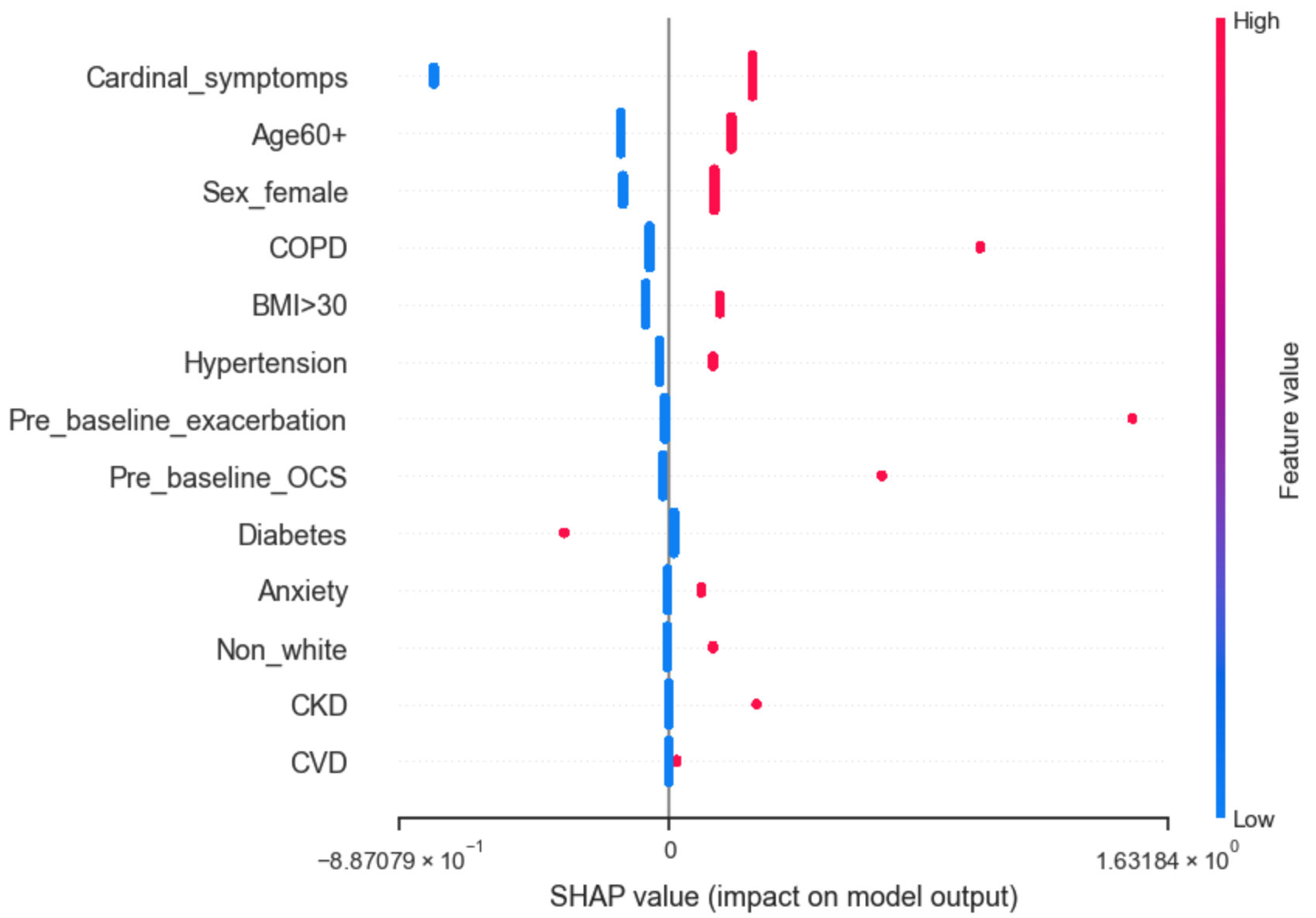 | 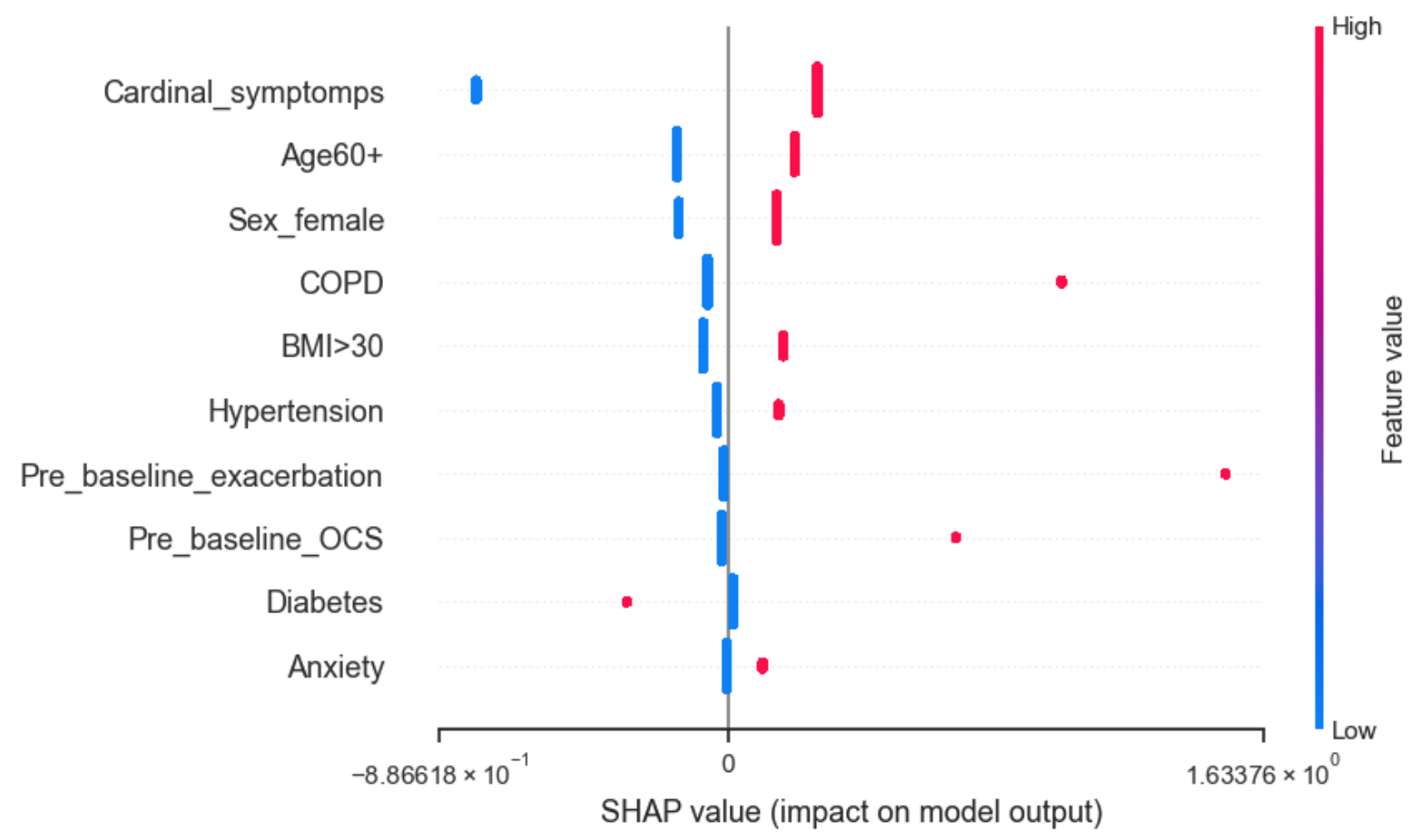 | 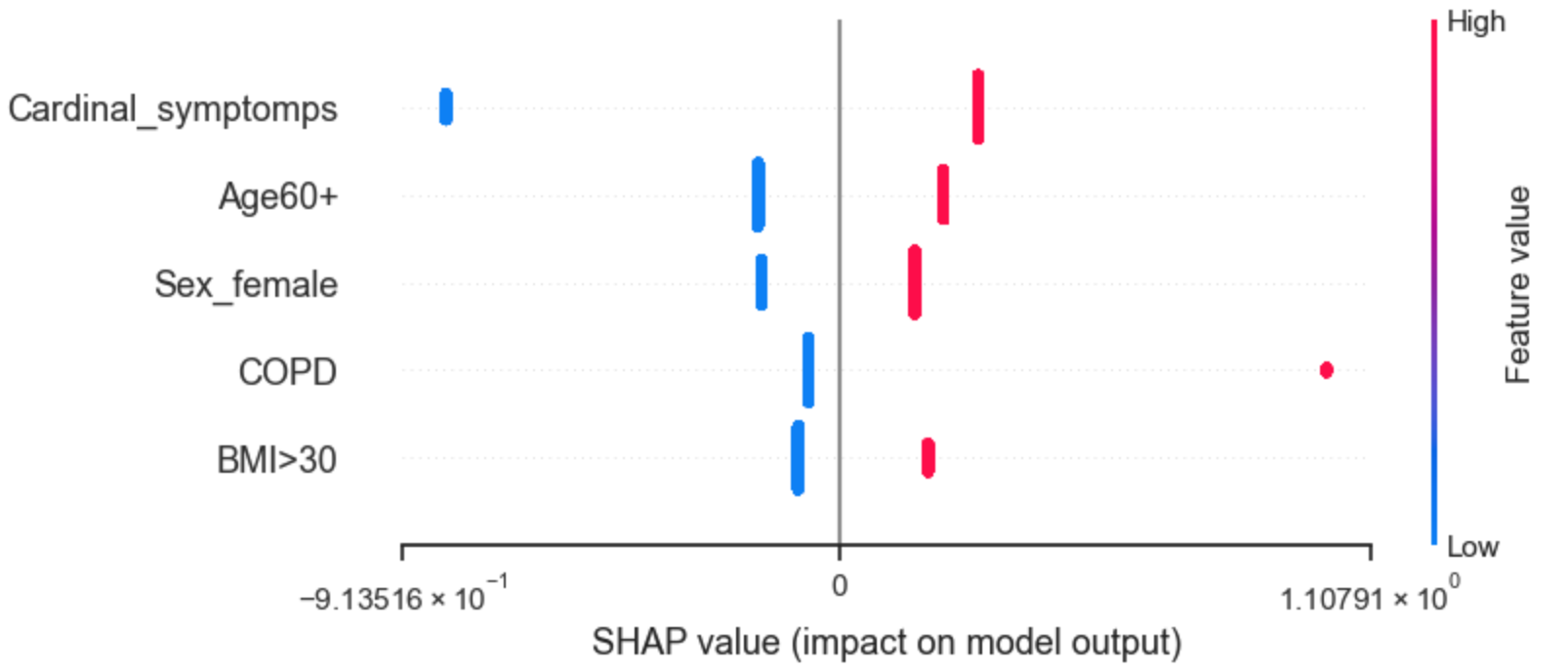 |

Table SA 7. Log-likelihood test results for candidate logistic regression models.

| **Outcome specification and prevalence** | **Covariate combinations** | **Log-likelihood test result compared with the following row**  **Likelihood ratio statistic, Degree of freedom, p-value** | **Interpretation** | **Final model selection** |
| --- | --- | --- | --- | --- |
| 1-year asthma exacerbation + non-repeat OCS prescription^1^  12.24% | Full | 0.2545, 1, p=0.61 | Full model does not improve the model fit compared to the near full model | Although the simple model does not improve the fit compared to the basic model, we have chosen it as the optimal model because it incorporates all sociodemographic variables, as well as CKD and CVD, which are included in this study to demonstrate the impact of differential privacy on rare variables. |
|  | Near full | 1.2764, 1, p=0.25 | Near full model does not improve the model fit compared to the partial model |  |
|  | Partial | 1.2184, 3, p=0.75 | Partial model does not improve the fit compared to the simple model |  |
|  | Simple | 4.8448, 3, p=0.18 | Simple model does not improve improves the fit compared to the basic model |  |
|  | Basic | 272.9568, 5, p<0.0001 | Simple model significantly improves the fit compared to the basic model |  |
|  | Minimal |  |  |  |

^1^Prednisolone oral corticosteroid
