# Supplementary material for: An empirical assessment of differential privacy in real-world observational data: a case-control study of asthma exacerbation in UK Biobank linked with electronic health records
Source: J Am Med Inform Assoc. 2025 Jun 18;32(8):1328–39. doi: 10.1093/jamia/ocaf090 (PMC12277706; doi:10.1093/jamia/ocaf090)
Supplement: ocaf090_Supplementary_Data [file ocaf090_supplementary_data.zip › ocaf090_Supplementary_Data/Supplementary_B_SB.docx]

# Supplementary materials B (SB)

**List of supplementary figures**

[Figure SB 1. Flowchart of patient inclusion and exclusion for primary and sensitivity analyses. 2](#_Toc196144821)

**List of supplementary tables**

[Table SB 1. Overview of study design. 3](#_Toc196144822)

[Table SB 2. Strengthening of Reporting of Observational Studies in Epidemiology (STROBE) checklist. 4](#_Toc196144823)

[Table SB 3. Reference and differentially private adjusted odds ratios (OR) of risk factors across epsilons (cases=2714, controls=19451). 6](#_Toc196144824)

[Table SB 4. Reference and differentially private 1:1 matched and adjusted ORs of risk factors across epsilons (cases=2714, controls=2713). 8](#_Toc196144825)

[Table SB 5. Reference vs differentially private adjusted odds ratios with different random states (cases=2714, controls=19451). 10](#_Toc196144826)

[Table SB 6. Reference vs differentially private 1:1 matched adjusted odds ratios with different random states (cases=2714, controls=2713). 14](#_Toc196144827)

[Table SB 7. Sensitivity analysis of model performance metrics under differential privacy with different random states (cases=2714, controls=19451). 17](#_Toc196144828)

[Table SB 8. SHAP values of differentially private models in adjusted model (logistic regression) across epsilons (cases=2714, controls=19451). 18](#_Toc196144829)

[Table SB 9. Summary of the sensitivity analyses. 20](#_Toc196144830)

[Table SB 10. Reference and differentially private unadjusted odds ratios (OR) of risk factors across epsilons (cases=2714, controls=19451). 22](#_Toc196144831)

[Table SB 11. Reference and differentially private 1:2 matched and adjusted ORs of risk factors across epsilons (cases=2714, controls=5334) . 24](#_Toc196144832)

[Table SB 12. Reference and differentially private 1:4 matched and adjusted ORs of risk factors across epsilons (cases=2714, controls=9736) . 26](#_Toc196144833)

[Table SB 13. Standardized mean difference (SMD) of non-private and differentially private propensity scores (age and Asthma duration are normalized). 28](#_Toc196144834)

[Table SB 14. Distribution of the age in controls matched using non-DP vs DP propensity scores. 29](#_Toc196144835)

[Table SB 15. Distribution of asthma duration in controls matched using non-DP vs DP propensity scores. 30](#_Toc196144836)

[Table SB 16. Differences in the mean of age between controls matched using non-DP vs DP propensity scores. 31](#_Toc196144837)

[Table SB 17. Differences in the mean of asthma duration between controls matched using non-DP vs DP propensity scores. 31](#_Toc196144838)

[Table SB 18. Differences in binary confounders in controls matched using non-DP vs DP propensity scores. 31](#_Toc196144839)


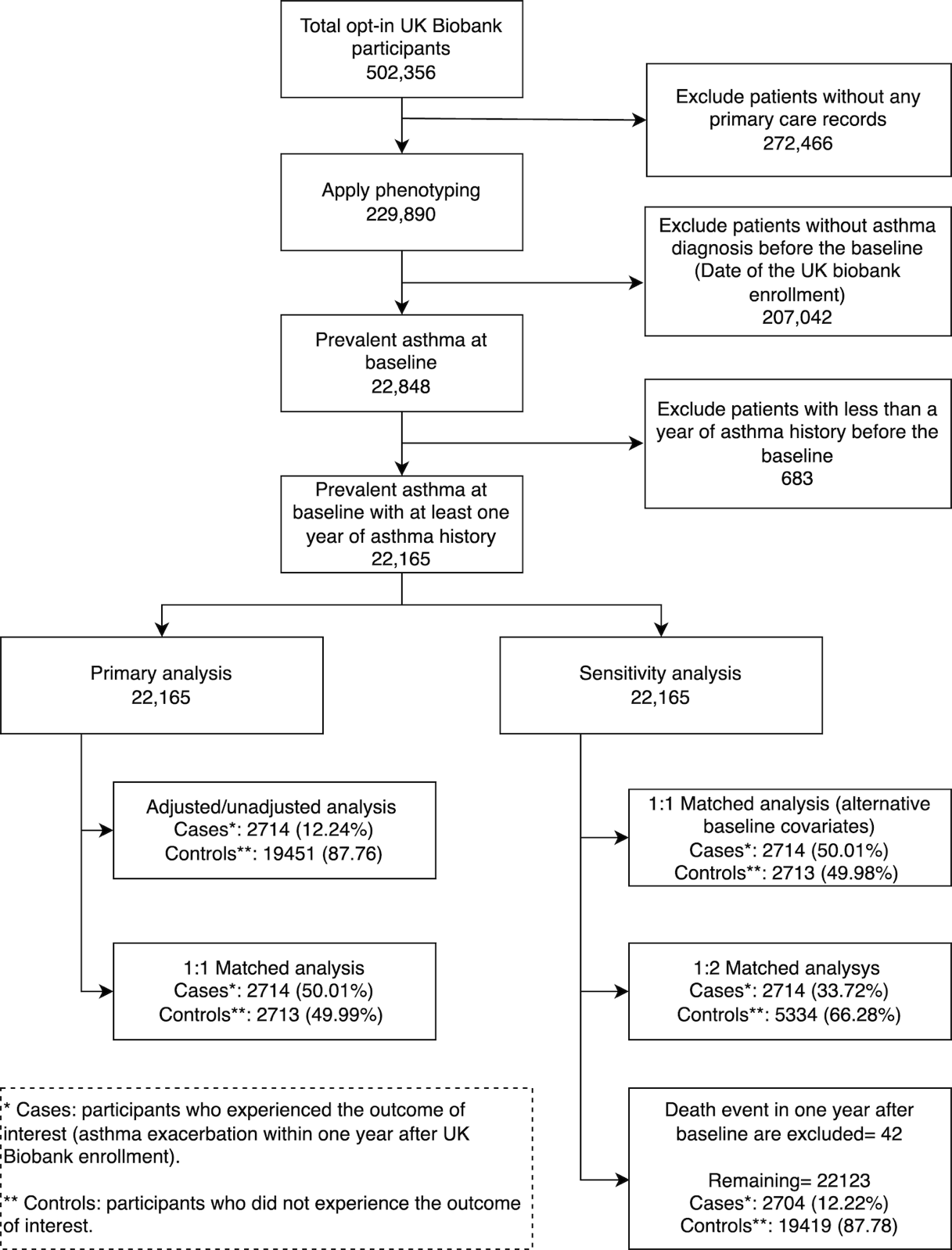


Figure SB 1. Flowchart of patient inclusion and exclusion for primary and sensitivity analyses.

Table SB 1. Overview of study design.

| **Study element** | **Description** |
| --- | --- |
| Study population | Participants with at least one year of asthma history at UK Biobank enrollment. |
| Exposures of interest | Risk factors as measured and collected at UK Biobank enrollment including:  - Sociodemographic characteristics  - Presence of selected comorbidities prior to enrollment  - Symptoms (e.g., cardinal symptoms) |
| Outcome of interest | Asthma exacerbation occurring within one year following the UK Biobank enrollment. |
| Cases and controls | Cases are participants who experienced the outcome of interest during the study period; controls are those who did not. |
| Study start date (index date or baseline) | UK Biobank enrollment was used as the index date, as many variables (e.g., smoking status, cardinal symptoms) were only recorded at that time. Because UK Biobank is a general-purpose cohort, these time-sensitive variables are not collected longitudinally. Age was calculated at enrollment to ensure comparability, since precise age at outcome is not available for controls. Given that the outcome window was limited to one year after enrollment, this provides a close and comparable estimate of age at the time of outcome for both cases and controls. |
| Study end date | One year after enrollment or the time of death, whichever occurred first. |
| Study type | This is an observational study using longitudinal electronic health records from UK Biobank. Unlike randomized controlled trials (RCTs), observational studies require careful adjustment for confounding through statistical techniques such as regression or matching. |
| Study design | A retrospective case-control study that begins with individuals with and without the outcome (i.e., cases and controls) and looks back to evaluate associations with prior risk factors (exposures) using odds ratios (ORs). |
| Handling of confounding bias | Confounding bias was addressed through covariate adjustment using logistic regression and through propensity score matching. |
| Sample size | The adjusted model used the full cohort of individuals with at least one year of asthma history prior to the index date. In matched models, sample size varied by matching ratio. For example, in 1:1 matching, each case was matched to one control, resulting in a matched subset approximately twice the number of cases. |
| Interpretation of odds ratios (OR) in a case control analysis | In case-control studies, the OR estimates the odds of prior exposure among cases relative to controls. For example, an OR of 1.35 (95% CI: 1.24-1.47) for female sex indicates that individuals who experienced the outcome (i.e., asthma exacerbation) had 35% higher odds of being female compared to those who did not. Because the confidence interval does not include 1, the association is statistically significant. |
| Null effect | If the 95% confidence interval (CI) of an OR includes 1, the association is not statistically significant, indicating no clear difference in the odds of risk factors between cases and controls. |

Table SB 2. Strengthening of Reporting of Observational Studies in Epidemiology (STROBE) checklist.

| **Item** | **No.** | **Checklist** |
| --- | --- | --- |
| Title and abstract | 1 | The title indicates the case-control study of the risk factors of asthma exacerbation as the main case study. |
| **Introduction** |  |  |
| Background/rationale | 2 | Presented in the Introduction section. |
| Objective | 3 | Presented in the Introduction section. |
| **Methods** |  |  |
| Study design | 4 | Described in the “Study Design” sub-section. |
| Setting | 5 | Described in the “Date source and study population” section. |
| Participants | 6 | Described in “Data source and study population” and Table 1. Matching criteria are described in the Methods section with the ratio of cases to control in the Results section. |
| Variables | 7 | Described in the “Variables and phenotypes” section. |
| Data sources/measurement | 8 | Details for each variable is provided in Supplementary A (SA), Tables SA3 and SA4. |
| Bias | 9 | Described in the “Variables and phenotypes” section. |
| Study size | 10 | Described in the “Date source and study population” section. |
| Quantitative variables | 11 | Dichotomization of variables is described in the “Statistical analysis” subsection |
| Statistical methods | 12 | a) All statistical methods and variables for propensity score matching are described in the “Statistical analysis” subsection.  b) N/A  c) The selected variables in UK Biobank were complete  d) NA. The entire cohort of asthma patients was used.  c) Described in the “Evaluation of the effects of DP” subsection |
| **Results** |  |  |
| Participants | 13 | The number of participants in adjusted and matched studies, and reasons for those numbers (e.g., exclusion of death events and k-nearest neighbor outputs) are provided in Table 1, the “Results”, and sensitivity analysis sections (Table SB12). The flowchart of participant inclusion and exclusion is shown in Figure SB1. |
| Descriptive data | 14 | Descriptive statistics for the entire data, cases, and controls are provided in Table 1. |
| Outcome data | 15 | Descriptive statistics in Table 1 are shown per outcome category. |
| Main results | 16 | Unadjusted, adjusted, and matched analysis process described in the “Results” section.  Boundaries of dichotomized variables are described in the “Study Design” section. |
| Other analyses | 17 | Described in the sensitivity analysis in Table SB 9. |
| **Discussion** |  |  |
| Key results | 18 | Described at the start of the “Discussion” section. |
| Limitations | 19 | Described at the end of the “Discussion” section. |
| Interpretation | 20 | Described at the start and middle of the “Discussion” section. |
| Generalizability | 21 | Described in the “Strengths and Limitations” section. |
| **Other information** |  |  |
| Funding | 22 | Described in the “Funding” section. |

Table SB 3. Reference and differentially private adjusted odds ratios (OR) of risk factors across epsilons (cases=2714, controls=19451).

| A) Age≥60  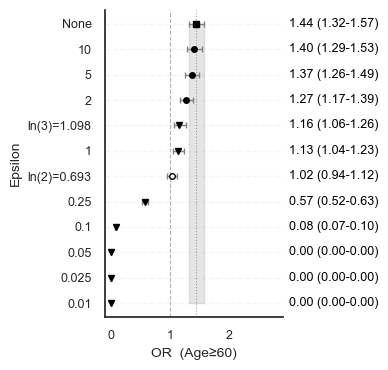 | B) Female sex  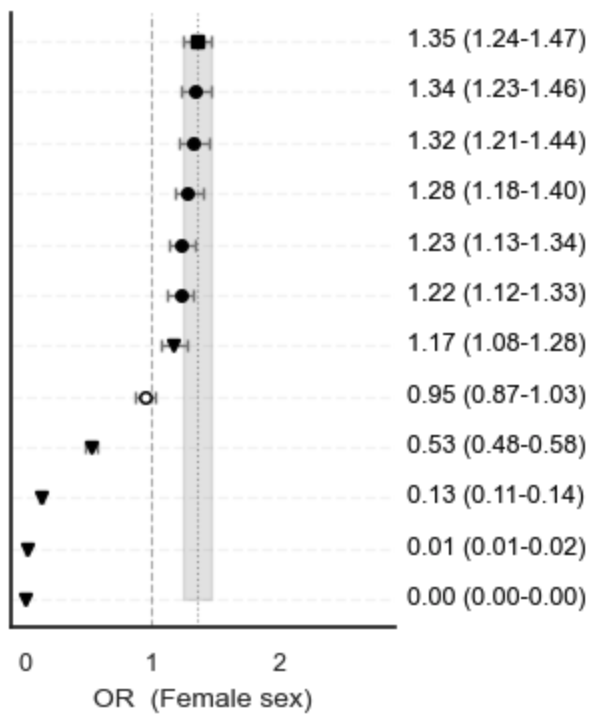 | C) Ethnicity (non-white)  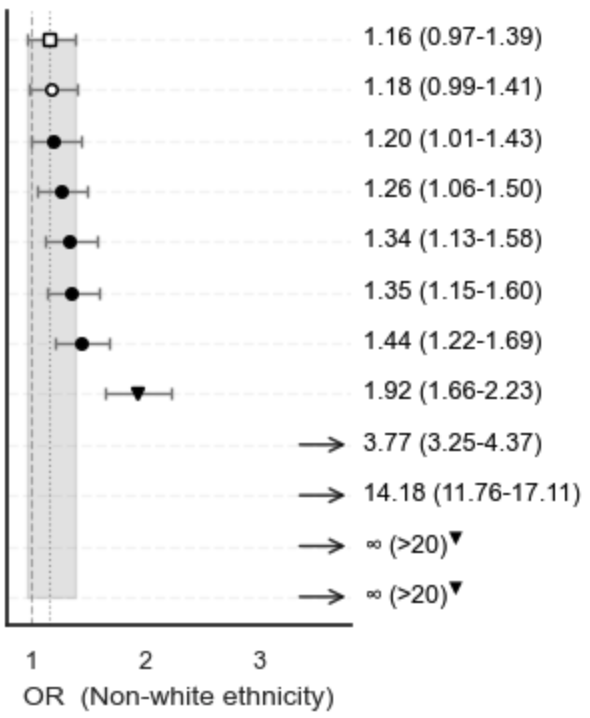 |
| --- | --- | --- |
| D) Anxiety  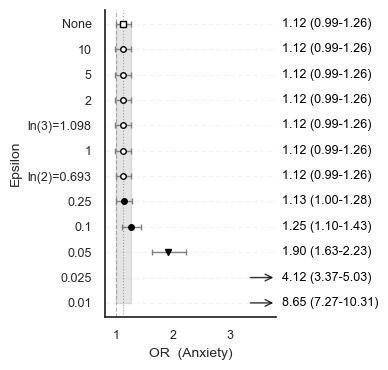 | E) BMI≥30  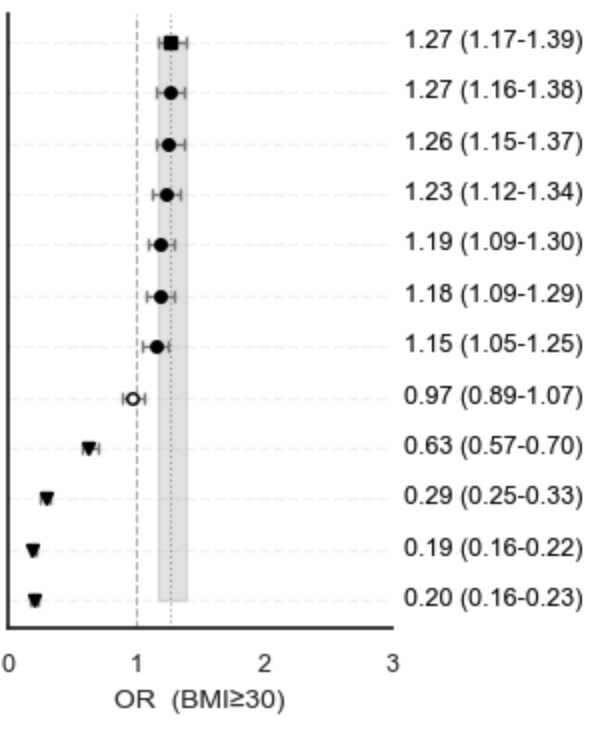 | F) CKD  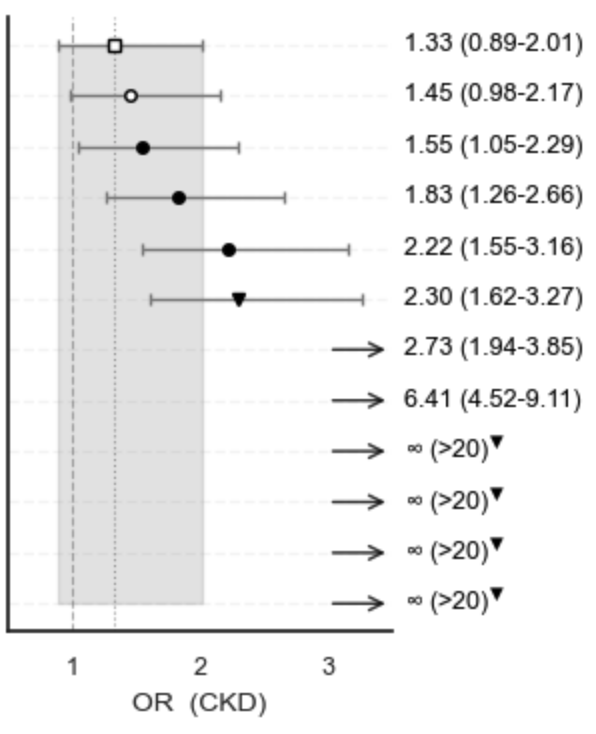 |
| G) COPD  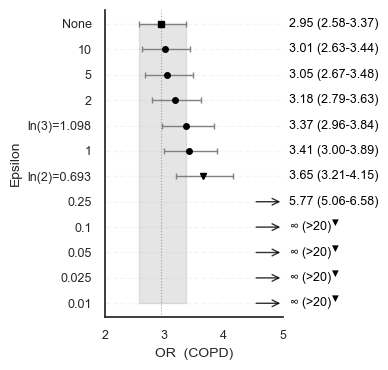 | H) CVD  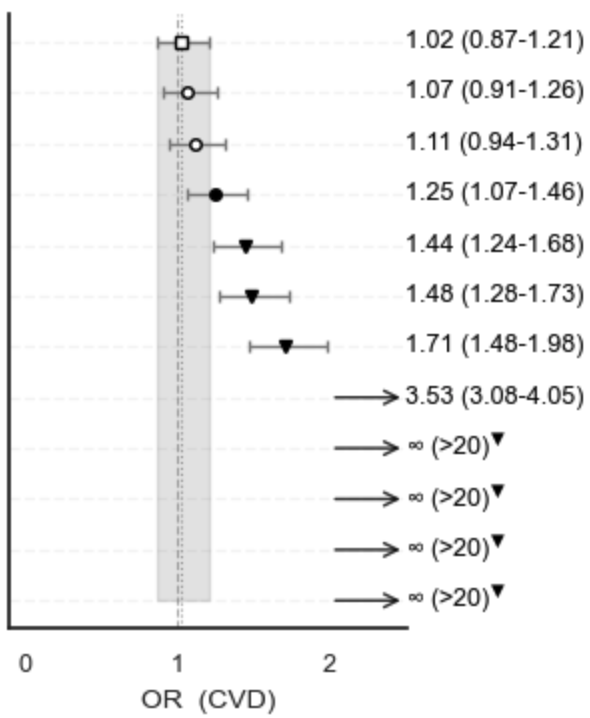 | I) Diabetes  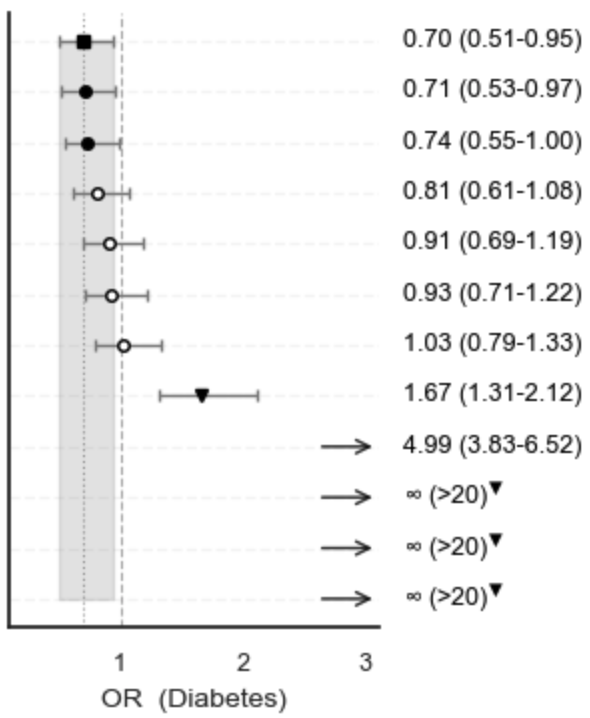 |
| 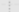■ Reference odds ratio (OR), p<0.05 □ Reference OR, not significant 95% Confidence interval of reference OR  ● Differentially private (DP) OR, p<0.05 ￮ DP OR, not significant ▼ DP OR, p<0.05, significantly different from reference OR  **Table continues on next page** | | |

*Table SB 3. (continued.) Reference and differentially private adjusted odds ratios (OR) of risk factors across epsilons (cases=2714, controls=19451).*

| J) Hypertension  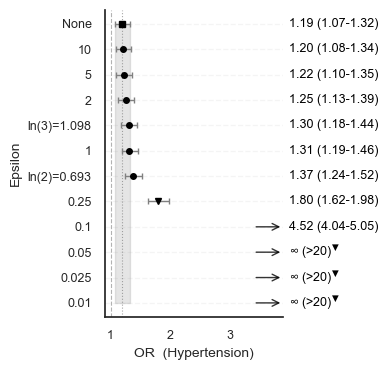 | K) Cardinal symptoms  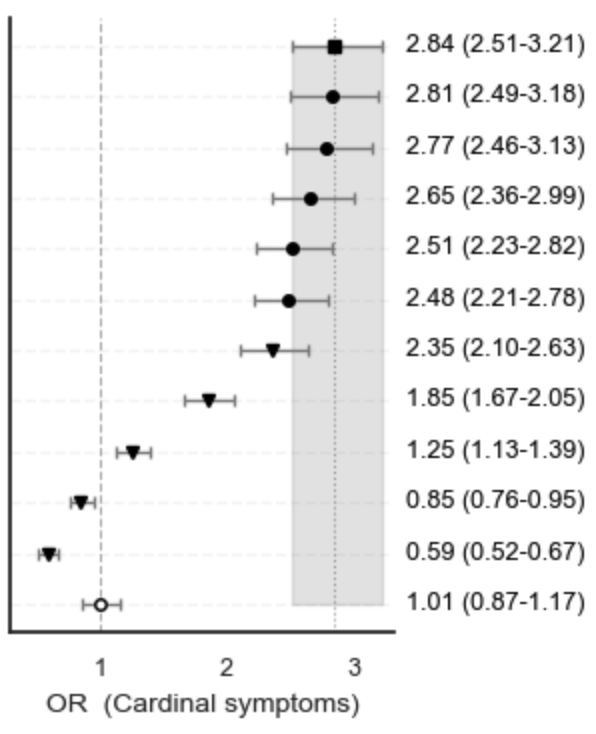 | L) Pre-baseline OCS  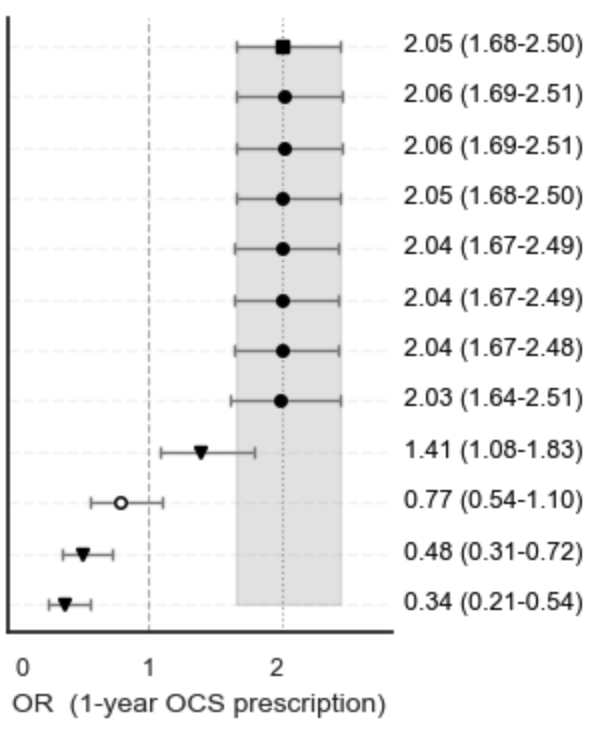 |
| --- | --- | --- |
| M) Pre-baseline exacerbation clinical  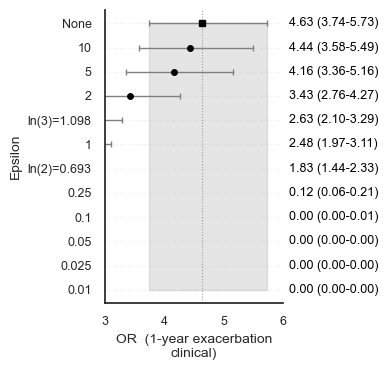 | N) (scaled)Pre-baseline exacerbation clinical  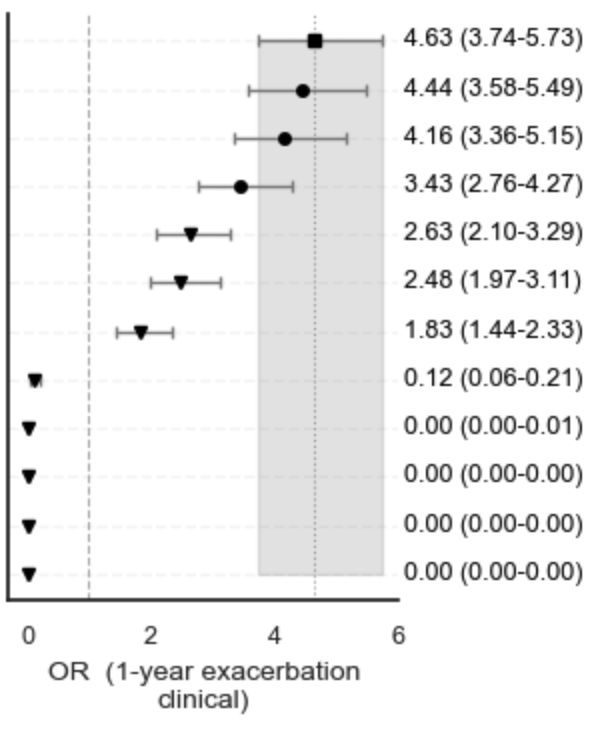 |  |
| 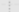■ Reference odds ratio (OR), p<0.05 □ Reference OR, not significant 95% Confidence interval of reference OR  ● Differentially private (DP) OR, p<0.05 ￮ DP OR, not significant ▼ DP OR, p<0.05, significantly different from reference OR | | |

Table SB 4. Reference and differentially private 1:1 matched and adjusted ORs of risk factors across epsilons (cases=2714, controls=2713).

| A) Age≥60  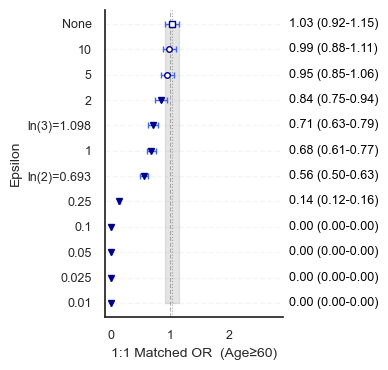 | B) Female sex  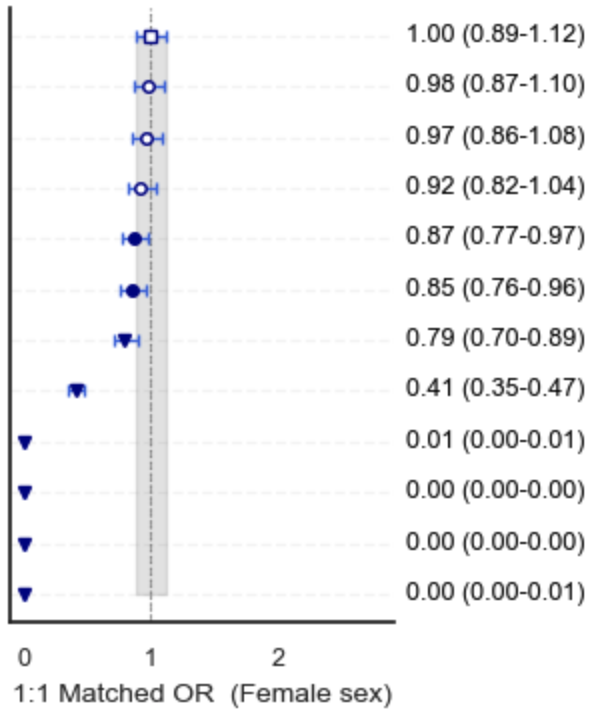 | C) Ethnicity (n on-white)  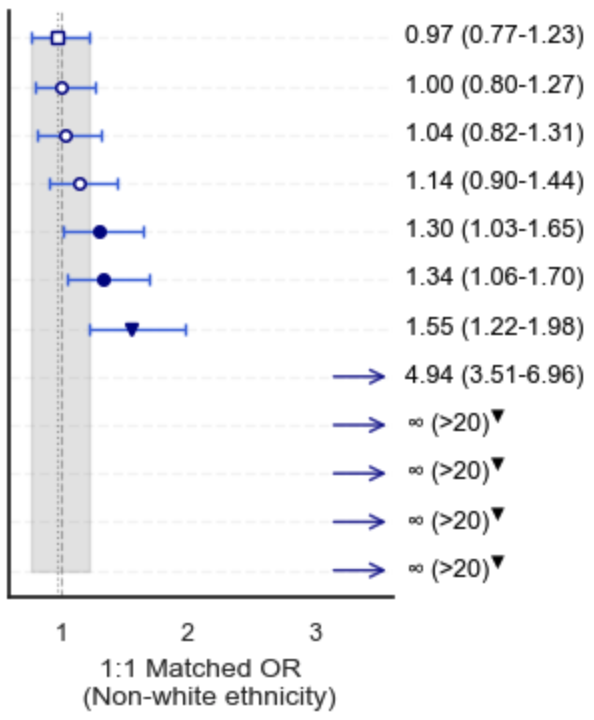 |
| --- | --- | --- |
| D) Anxiety  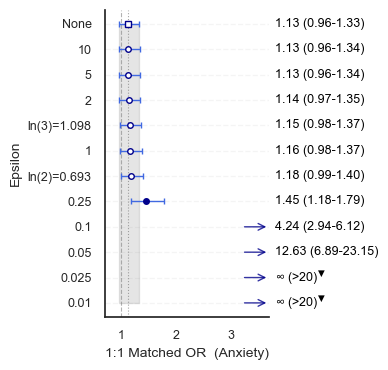 | E) BMI≥30  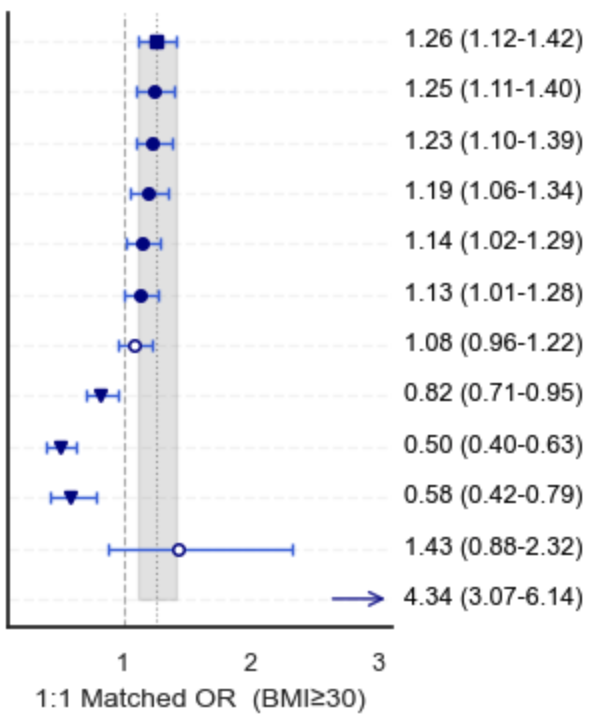 | F) CKD  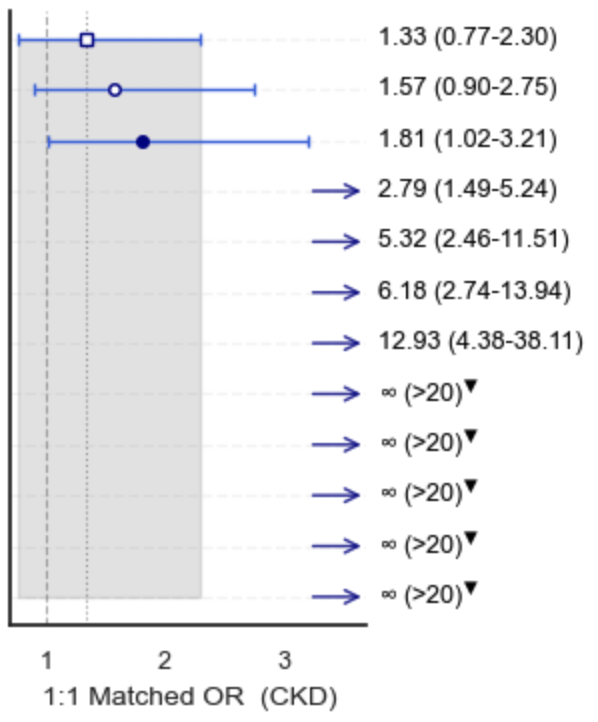 |
| G) COPD  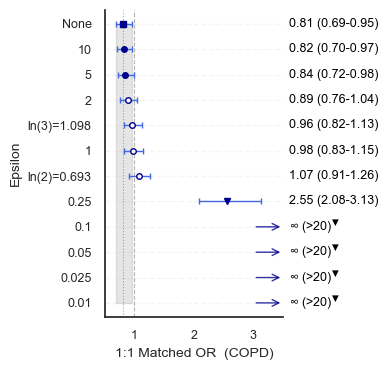 | H) CVD  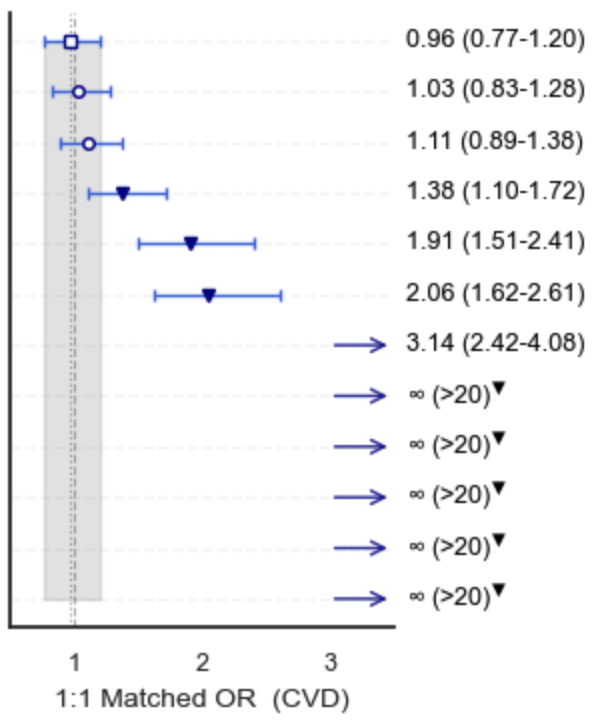 | I) Diabetes  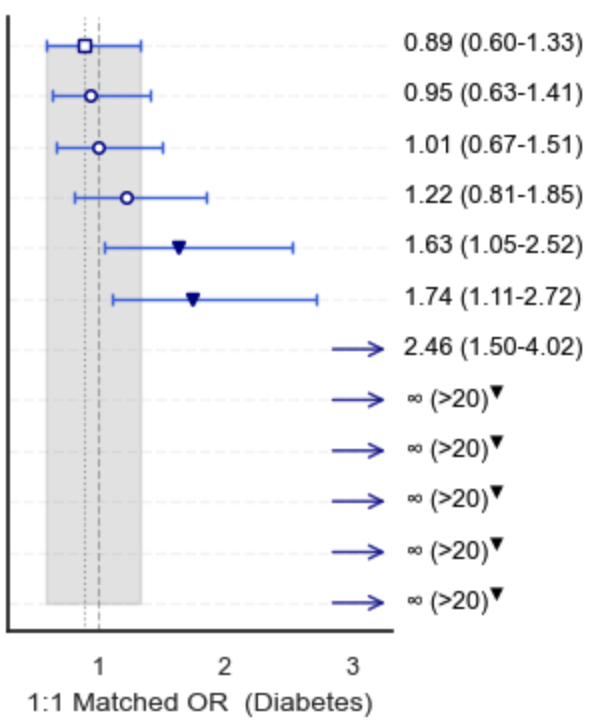 |
| 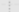■ Reference odds ratio (OR), p<0.05 □ Reference OR, not significant 95% Confidence interval of reference OR  ● Differentially private (DP) OR, p<0.05 ￮ DP OR, not significant ▼ DP OR, p<0.05, significantly different from reference OR  **Table continues on next page** | | |

*Table SB 4. (continued.) Reference and differentially private 1:1 matched and adjusted ORs of risk factors across epsilons (cases=2714, controls=2713).*

| J) Hypertension  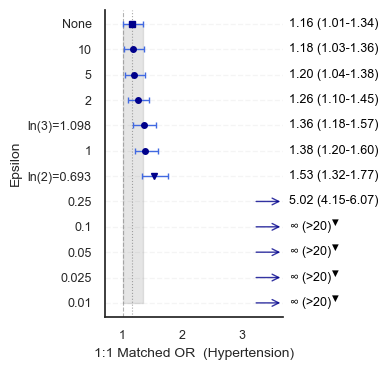 | K) Cardinal symptoms  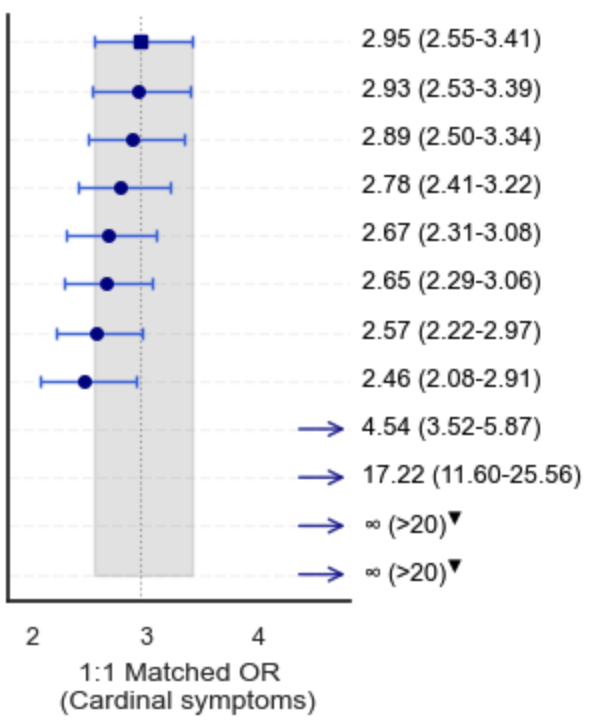 | L) Pre-baseline OCS  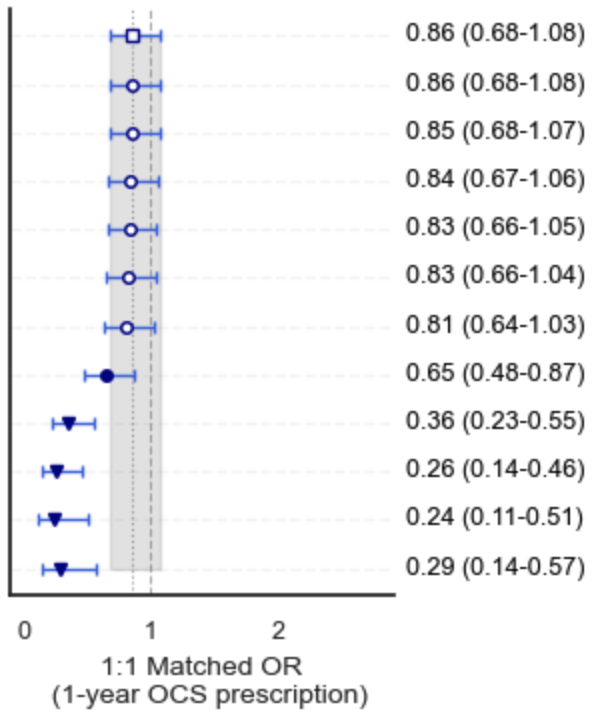 |
| --- | --- | --- |
| M) Pre-baseline exacerbation clinical  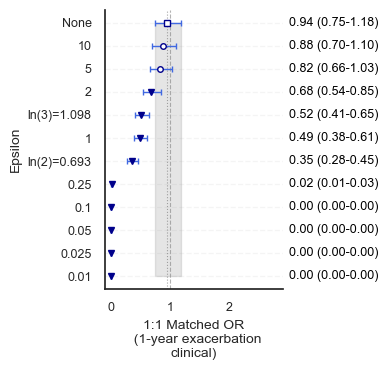 |  |  |
| 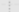■ Reference odds ratio (OR), p<0.05 □ Reference OR, not significant 95% Confidence interval of reference OR  ● Differentially private (DP) OR, p<0.05 ￮ DP OR, not significant ▼ DP OR, p<0.05, significantly different from reference OR | | |

Table SB 5. Reference vs differentially private adjusted odds ratios with different random states (cases=2714, controls=19451).

| Covariate | A) Random sate 27 | B) Random state 88 | C) Random state 77 |
| --- | --- | --- | --- |
| Age≥60 | 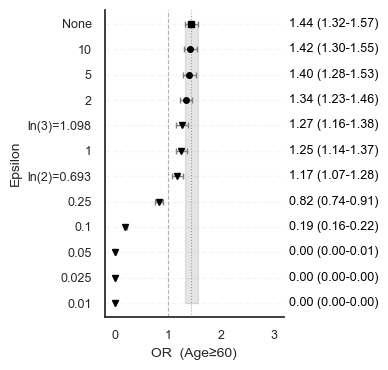 | 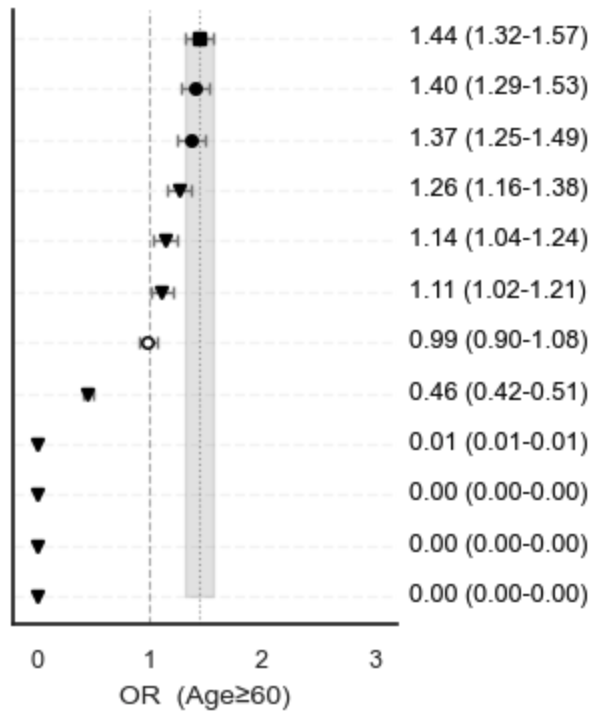 | 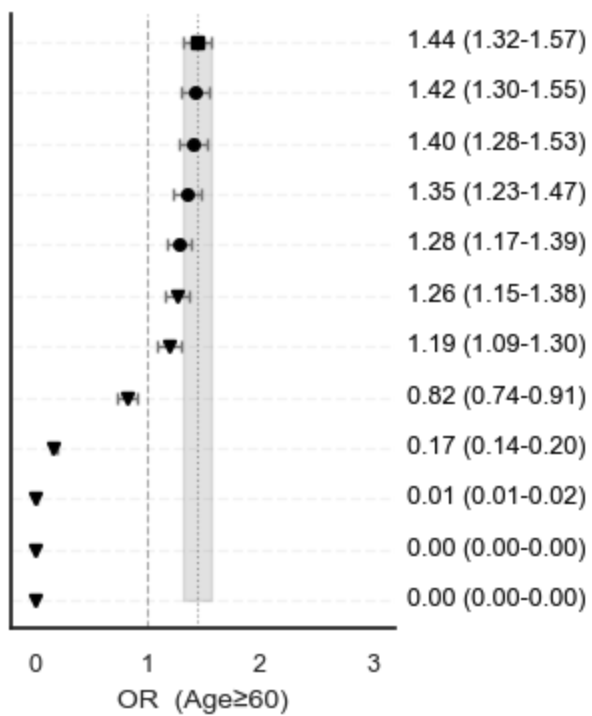 |
| Female Sex | 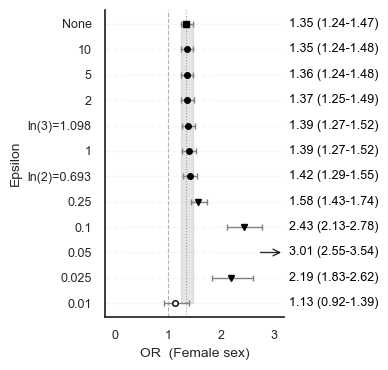 | 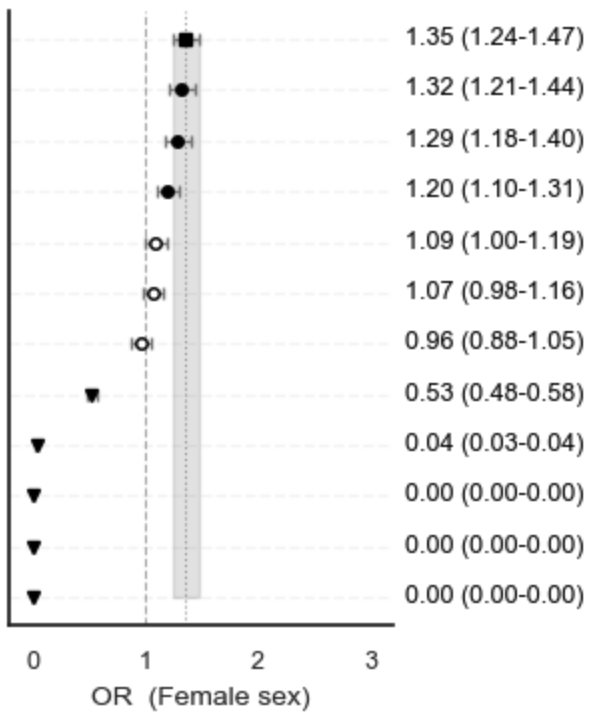 | 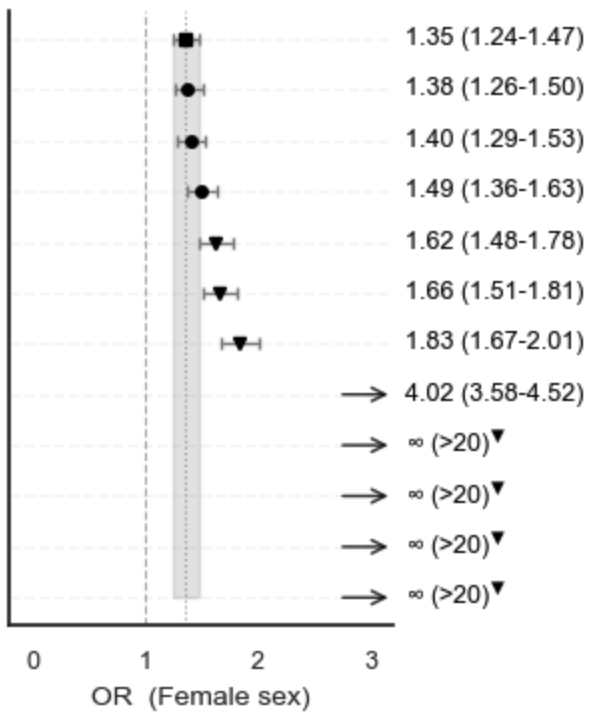 |
| Non-white ethnicity | 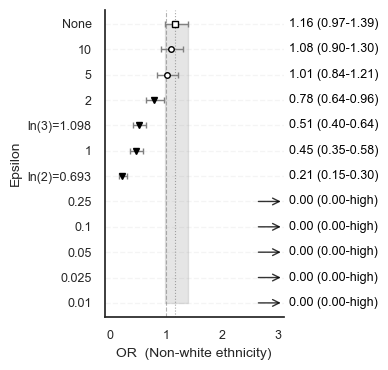 | 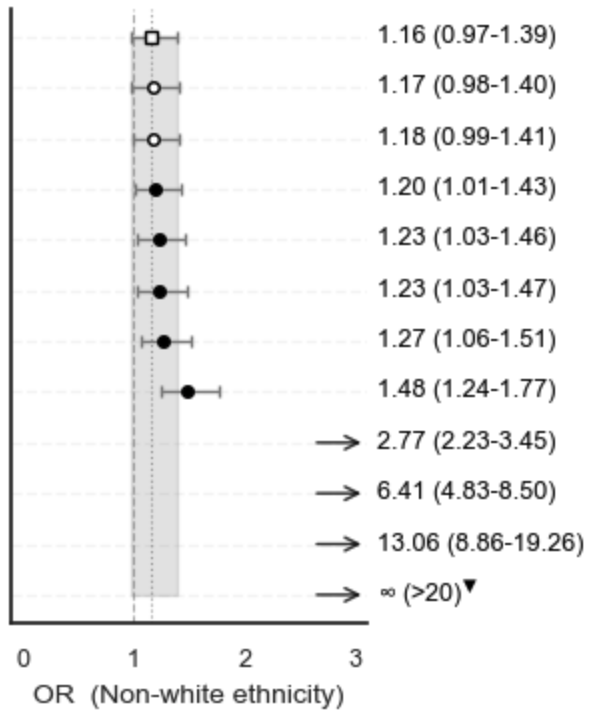 | 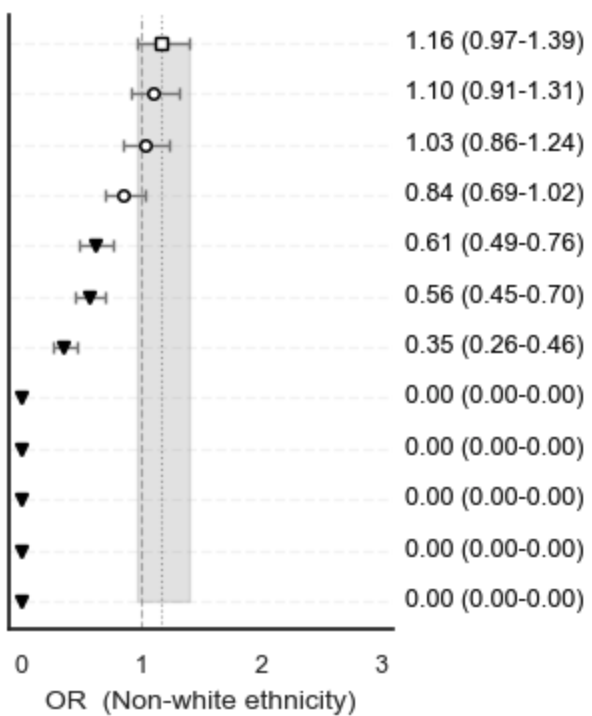 |
| Anxiety | 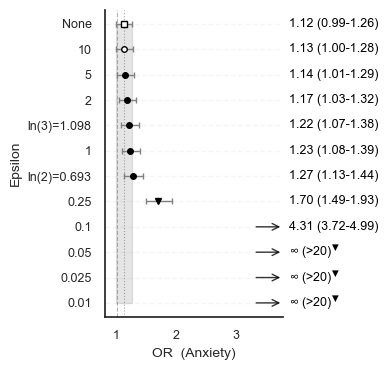 | 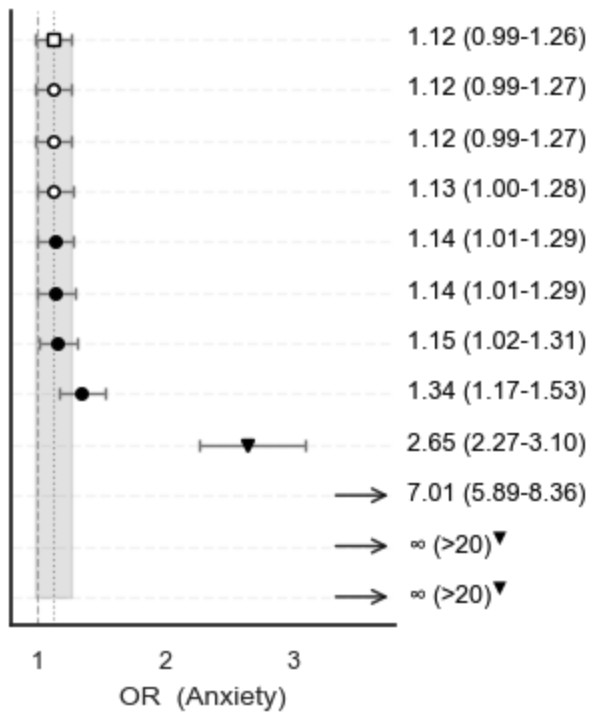 | 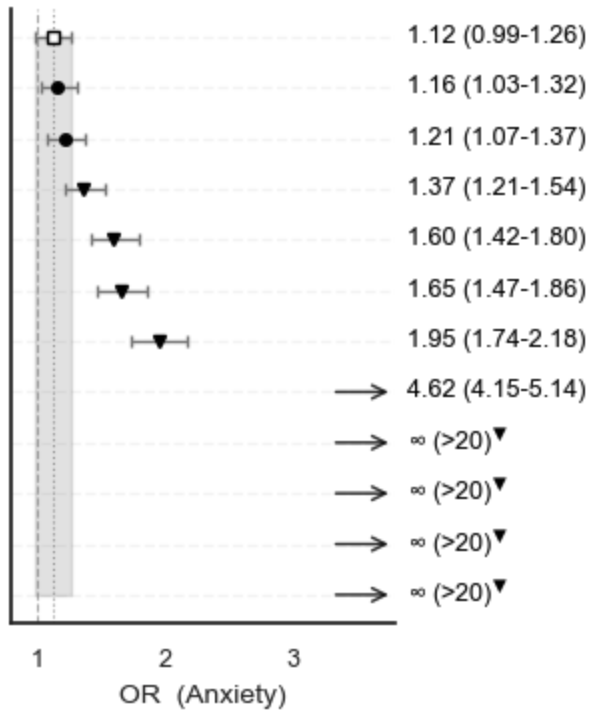 |
| 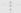■ Reference odds ratio (OR), p<0.05 □ Reference OR, not significant 95% Confidence interval of reference OR  ● Differentially private (DP) OR, p<0.05 ￮ DP OR, not significant ▼ DP OR, p<0.05, significantly different from reference OR  **Table continued on next page** | | | |

Table SB 5. (continued.) Reference vs differentially private adjusted odds ratios with different random states (cases=2714, controls=19451).

| Covariate | A) Random sate 27 | B) Random state 88 | C) Random state 77 |
| --- | --- | --- | --- |
| BMI≥30 | 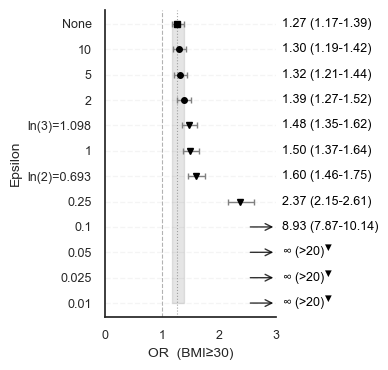 | 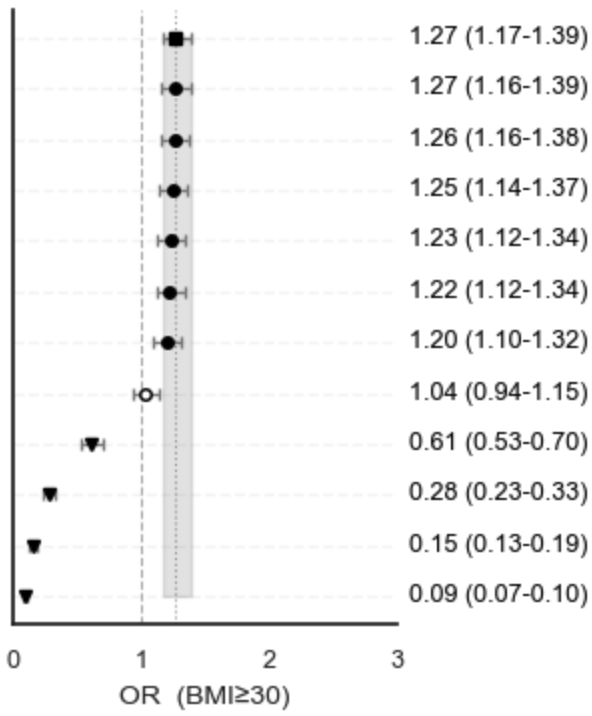 | 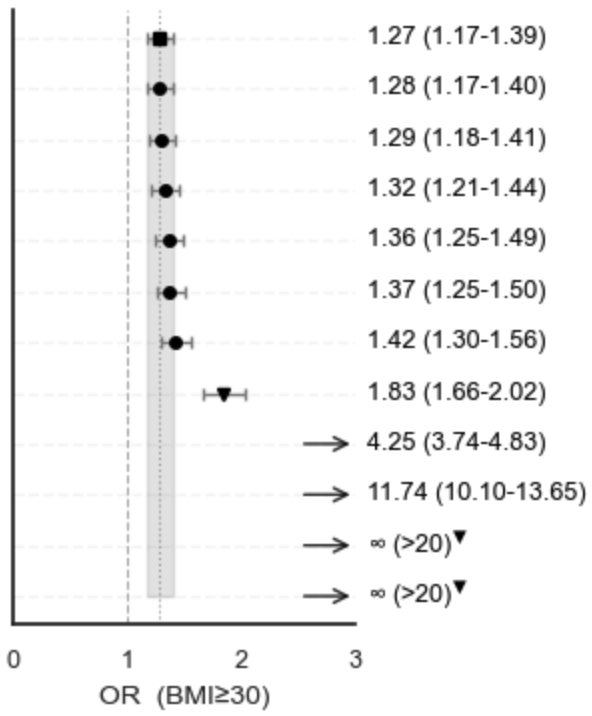 |
| CKD | 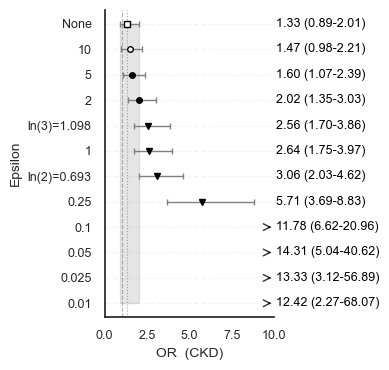 | 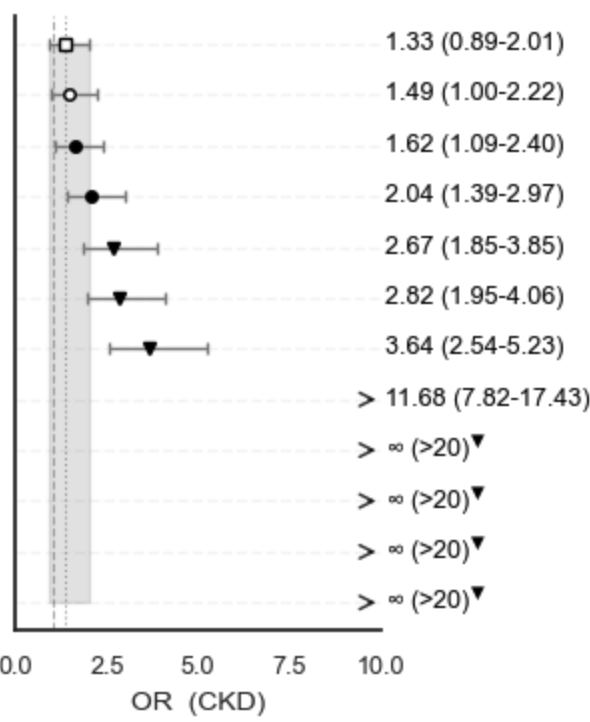 | 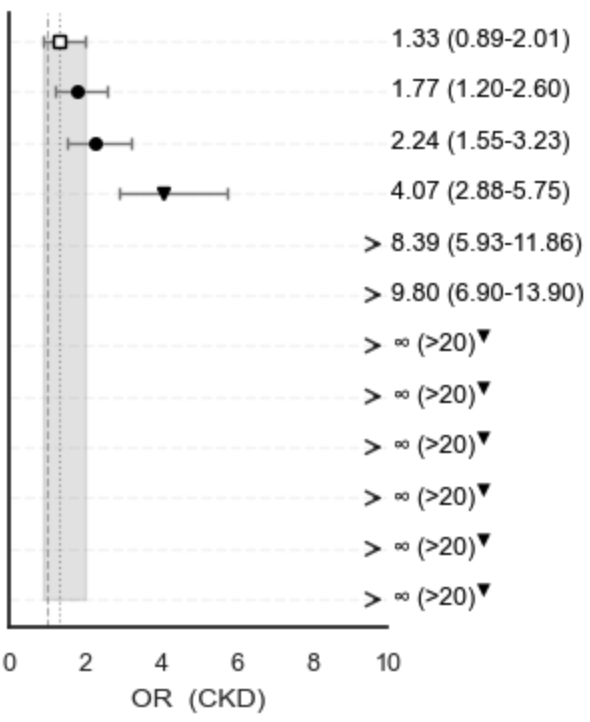 |
| COPD | 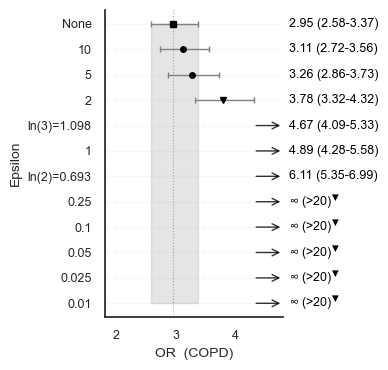 | 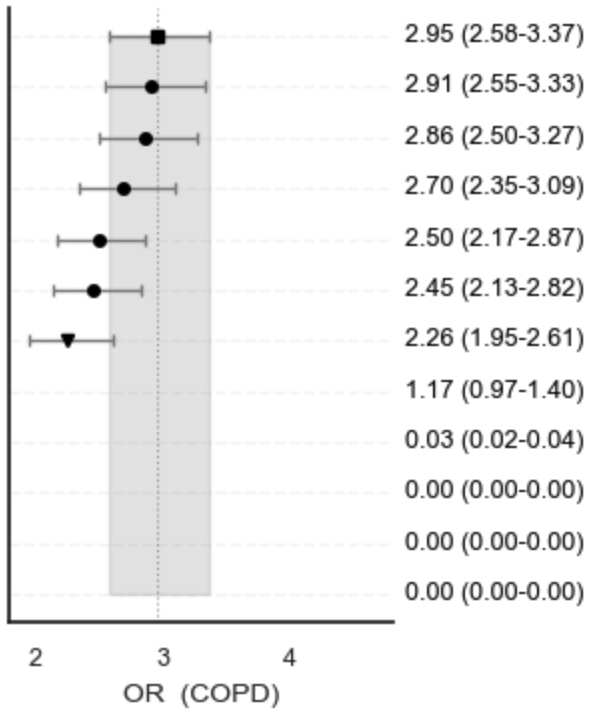 | 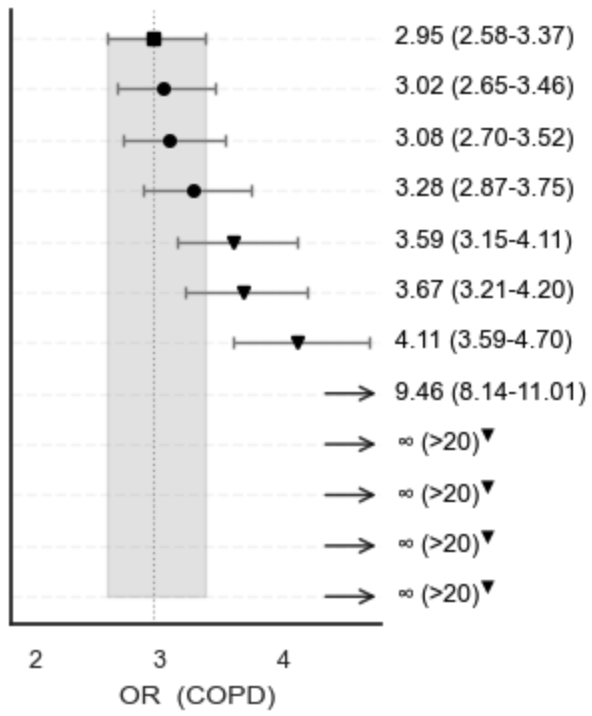 |
| CVD | 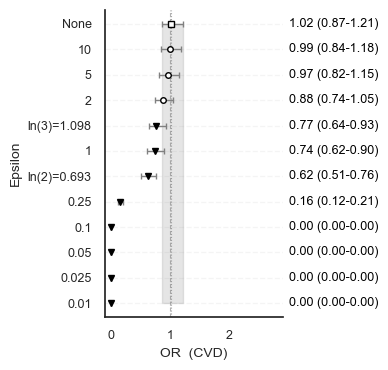 | 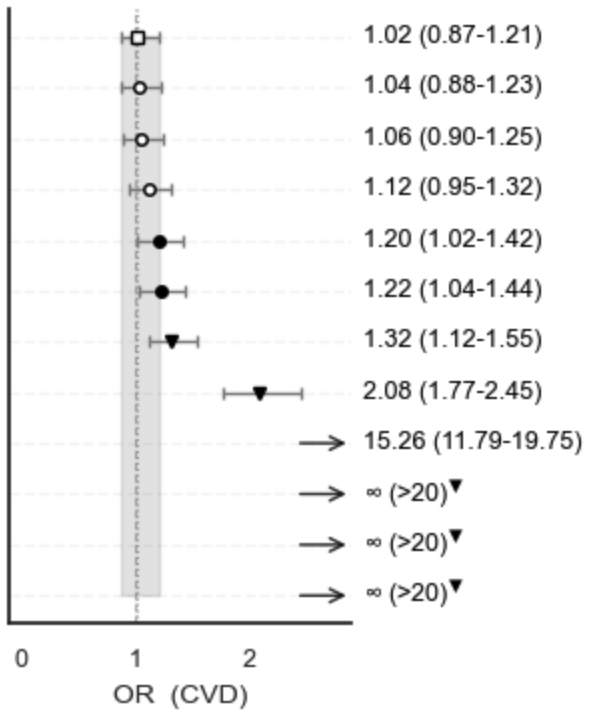 | 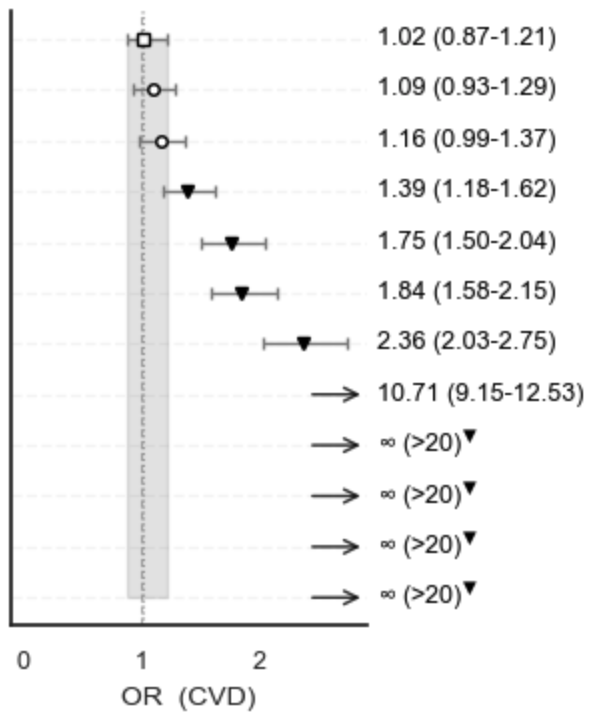 |
| 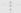■ Reference odds ratio (OR), p<0.05 □ Reference OR, not significant 95% Confidence interval of reference OR  ● Differentially private (DP) OR, p<0.05 ￮ DP OR, not significant ▼ DP OR, p<0.05, significantly different from reference OR  **Table continued on next page** | | | |

Table SB 5. (continued.) Reference vs differentially private adjusted odds ratios with different random states (cases=2714, controls=19451).

| Covariate | A) Random sate 27 | B) Random state 37 | C) Random state 77 |
| --- | --- | --- | --- |
| Diabetes | 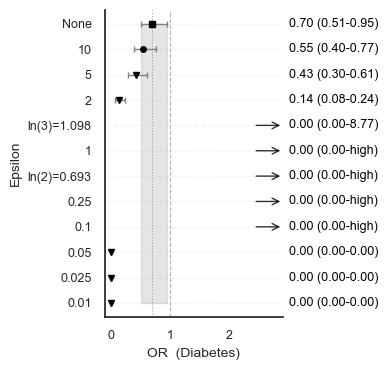 | 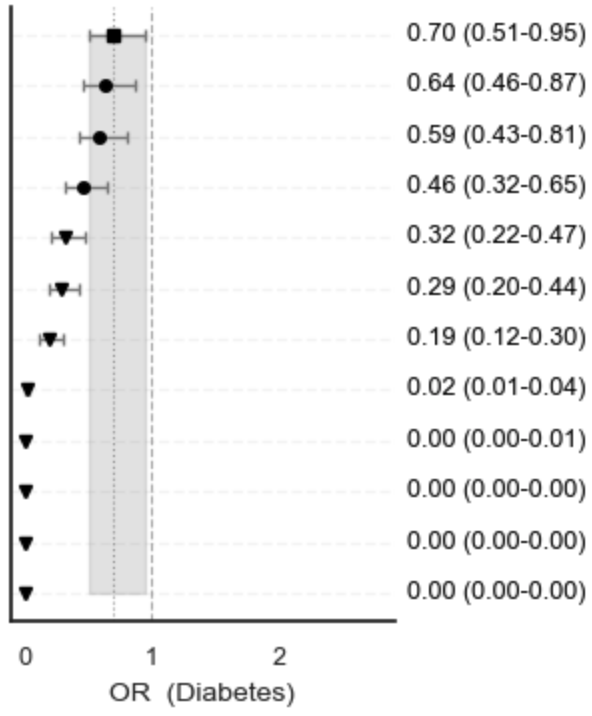 | 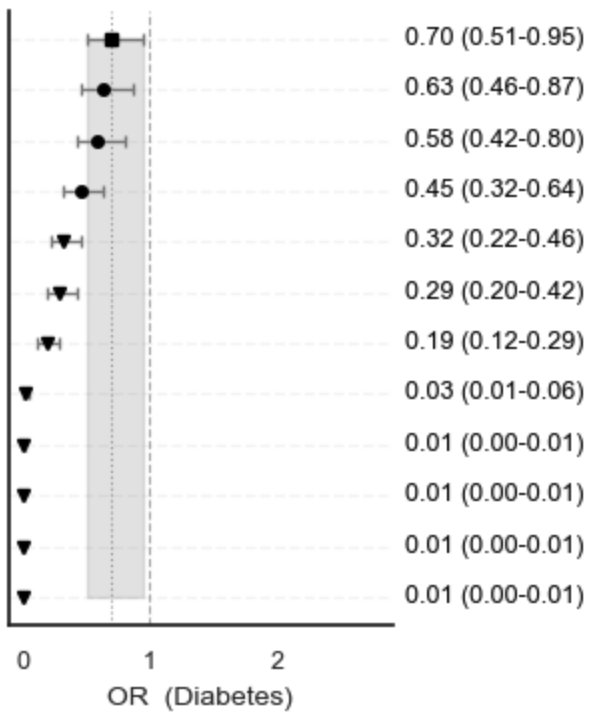 |
| Hypertension | 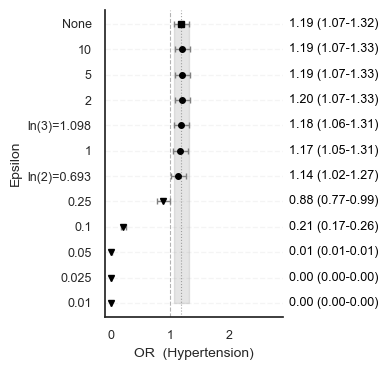 | 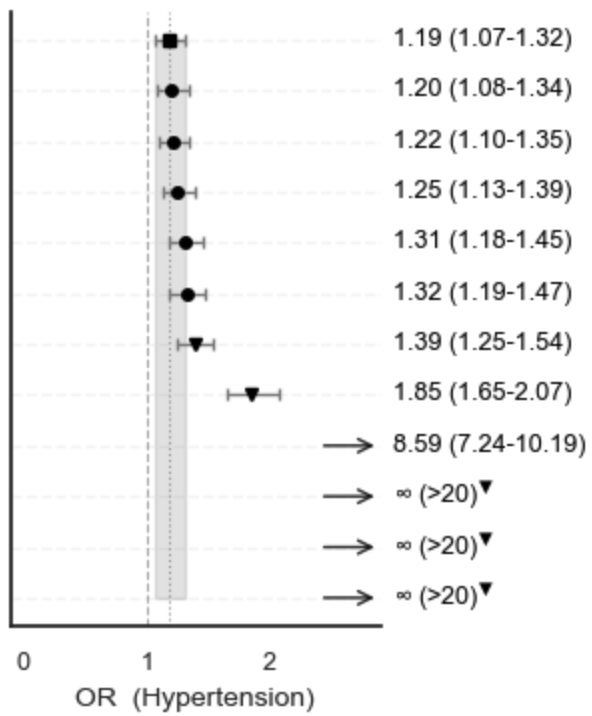 | 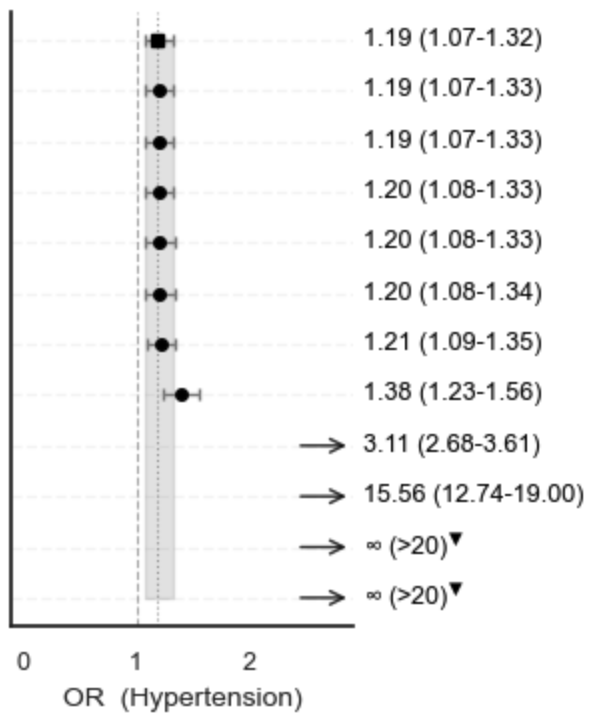 |
| Cardinal symptoms | 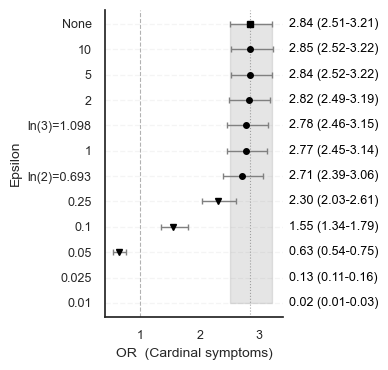 | 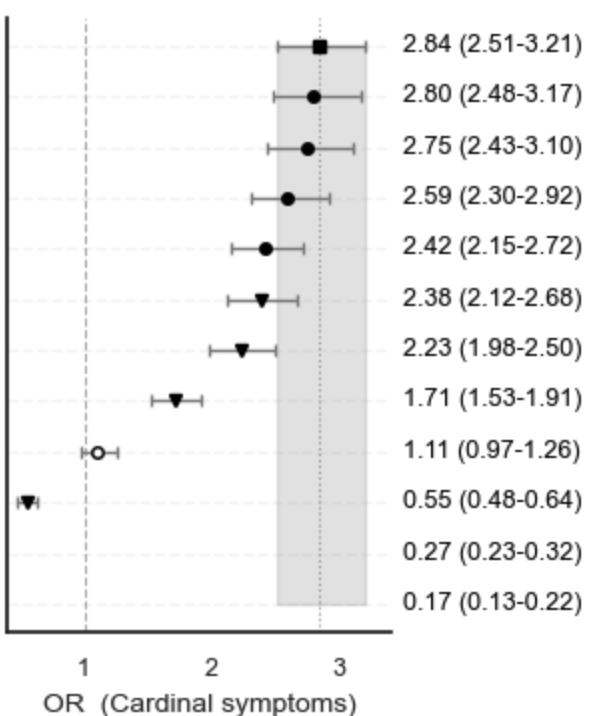 | 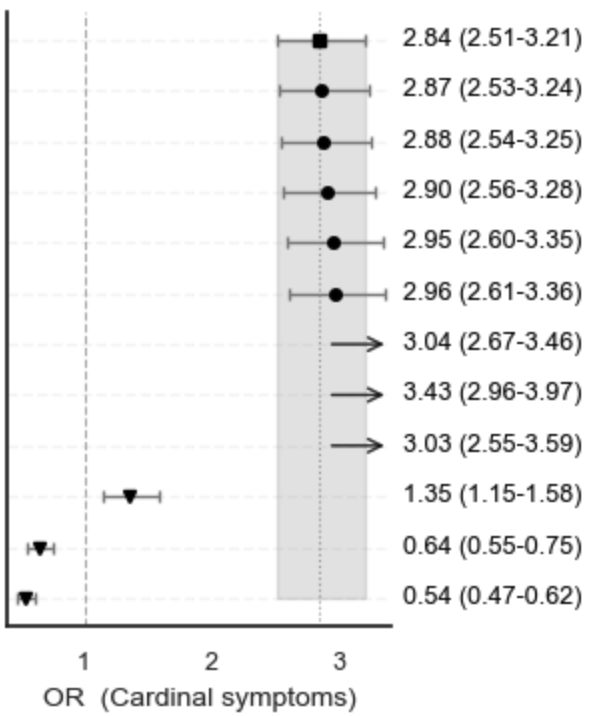 |
| Pre-baseline pe year prescription of oral coricosteroid (OCS) | 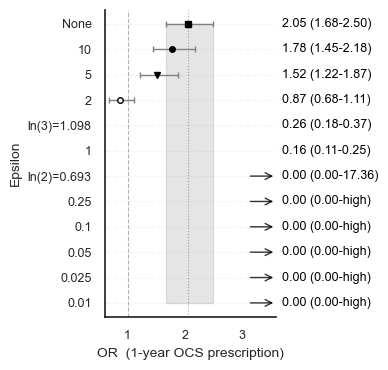 | 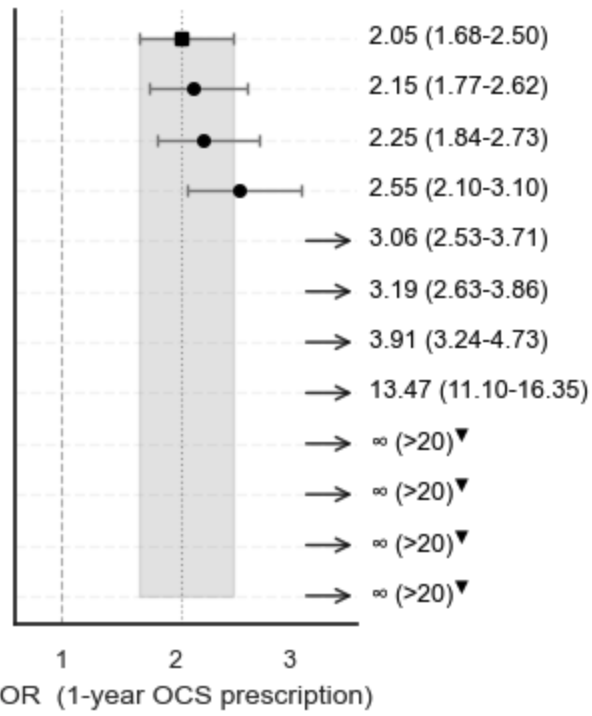 | 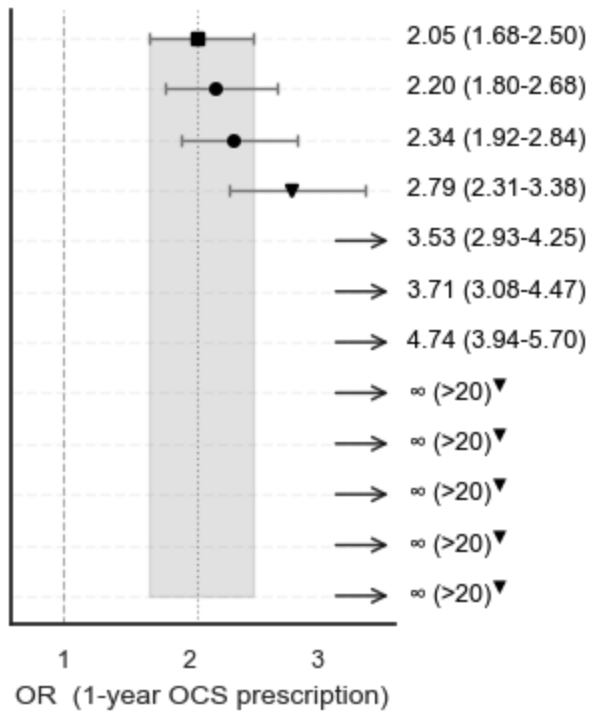 |
| 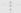■ Reference odds ratio (OR), p<0.05 □ Reference OR, not significant 95% Confidence interval of reference OR  ● Differentially private (DP) OR, p<0.05 ￮ DP OR, not significant ▼ DP OR, p<0.05, significantly different from reference OR  **Table continued on next page** | | | |

Table SB 5. (continued.) Reference vs differentially private adjusted odds ratios with different random states (cases=2714, controls=19451).

| Covariate | A) Random sate 27 | B) Random state 88 | C) Random state 77 |
| --- | --- | --- | --- |
| Pre-baseline one year clinical exacerbation events | 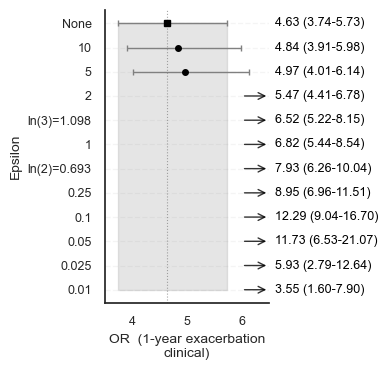 | 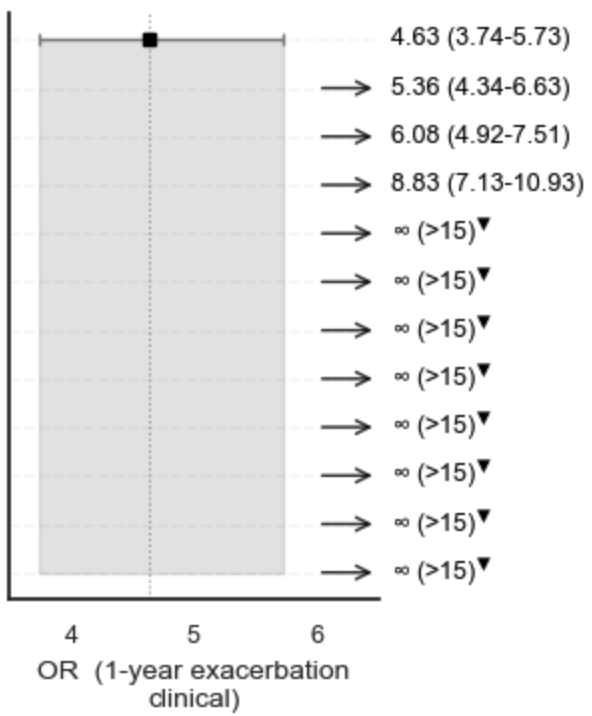 | 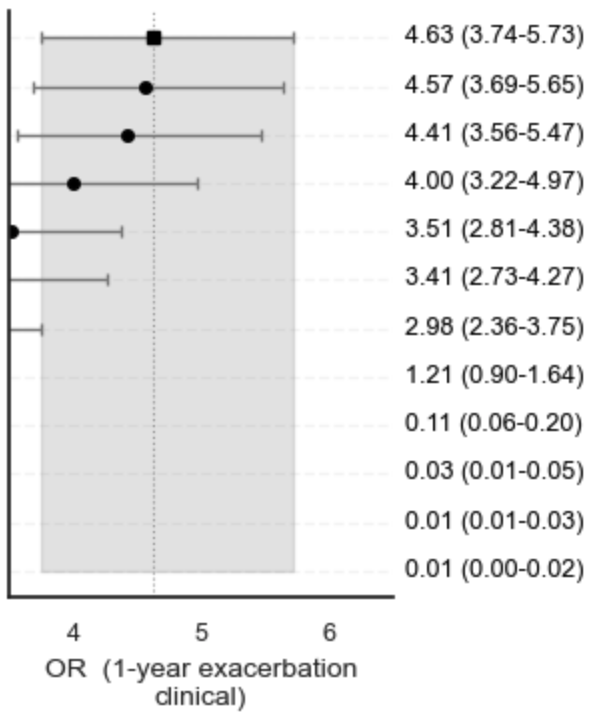 |
| Pre-baseline one year clinical exacerbation events  (scaled) | 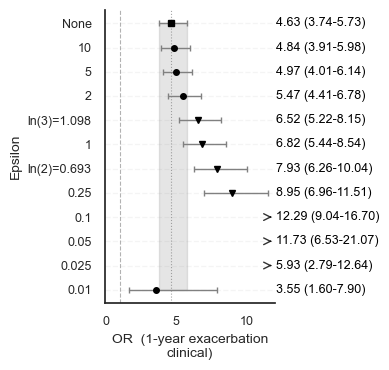 | 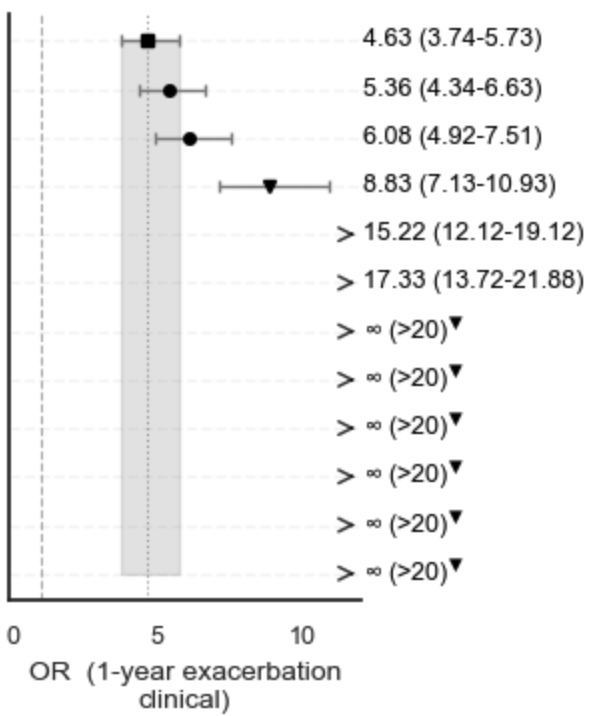 | 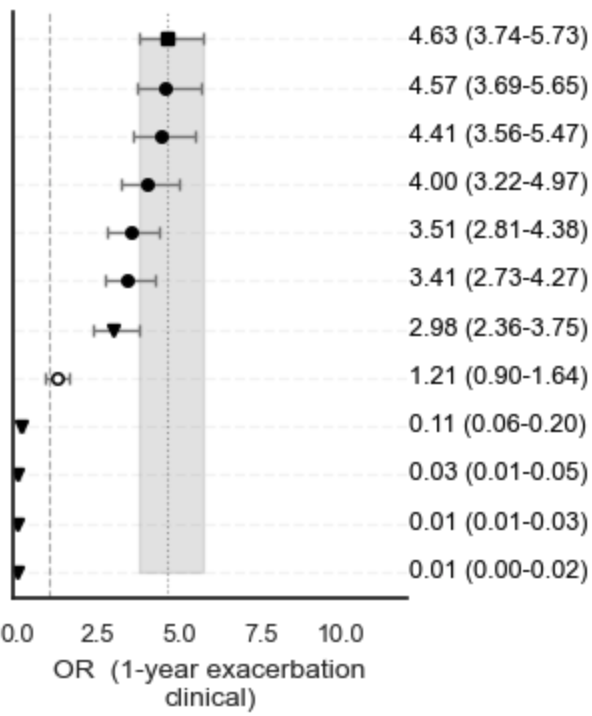 |
| 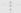■ Reference odds ratio (OR), p<0.05 □ Reference OR, not significant 95% Confidence interval of reference OR  ● Differentially private (DP) OR, p<0.05 ￮ DP OR, not significant ▼ DP OR, p<0.05, significantly different from reference OR | | | |

Table SB 6. Reference vs differentially private 1:1 matched adjusted odds ratios with different random states (cases=2714, controls=2713).

| Covariate | A) Random sate 27 | B) Random state 88 | C) Random state 77 |
| --- | --- | --- | --- |
| Age≥60 | 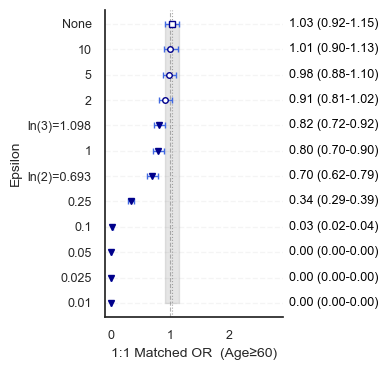 | 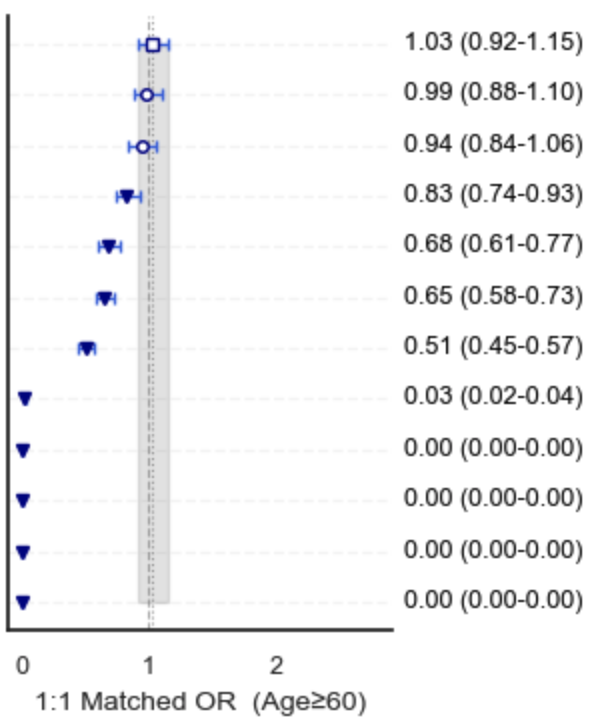 | 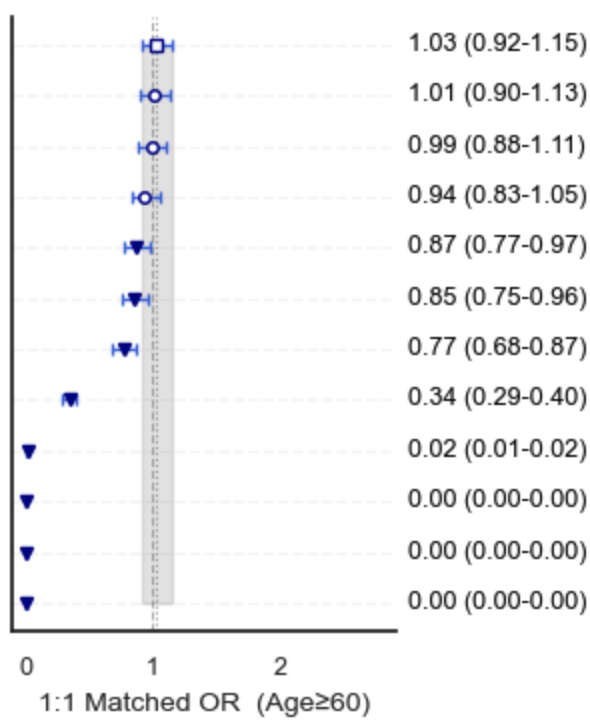 |
| Female Sex | 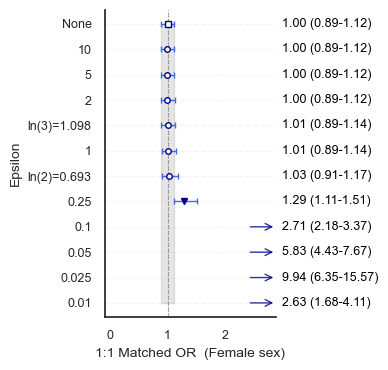 | 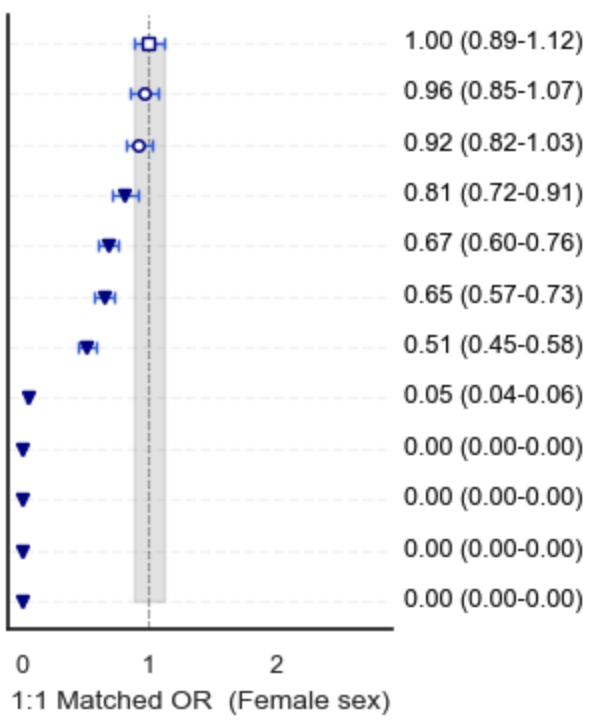 | 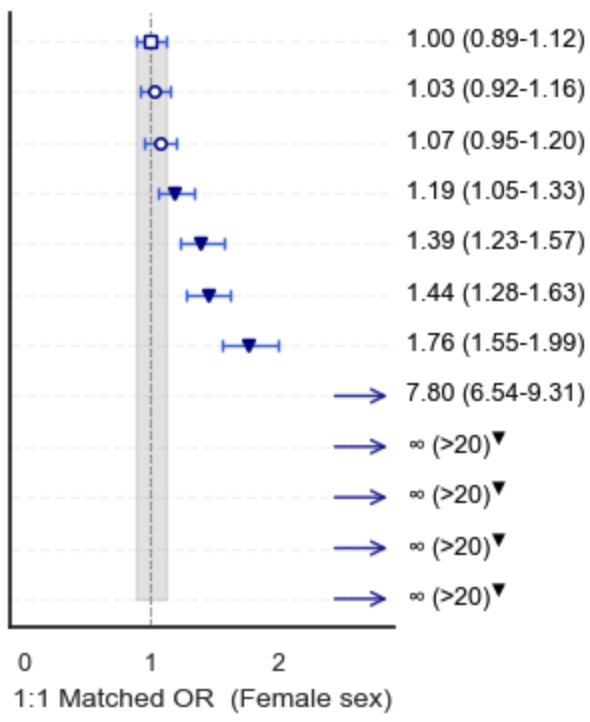 |
| Non-white ethnicity | 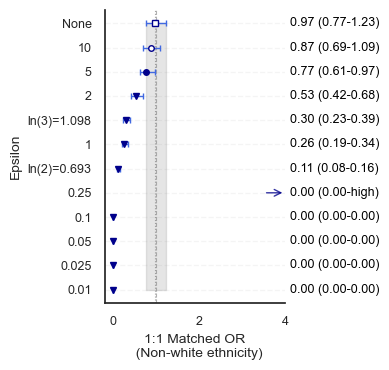 | 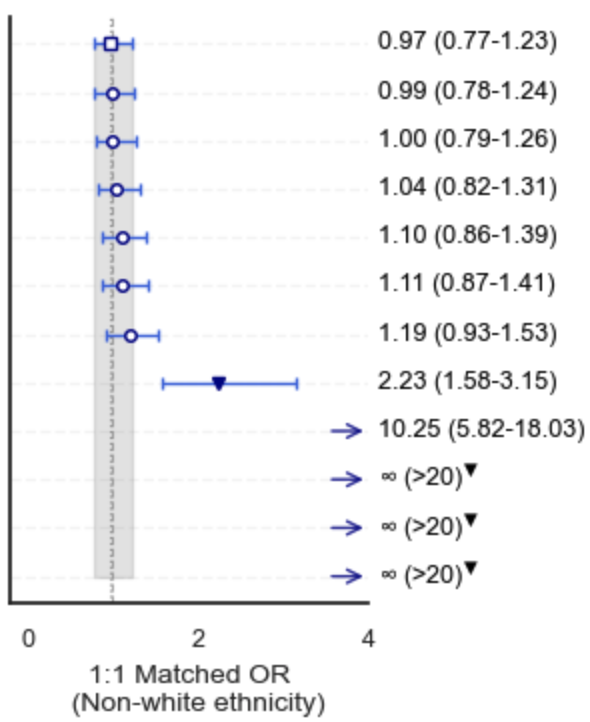 | 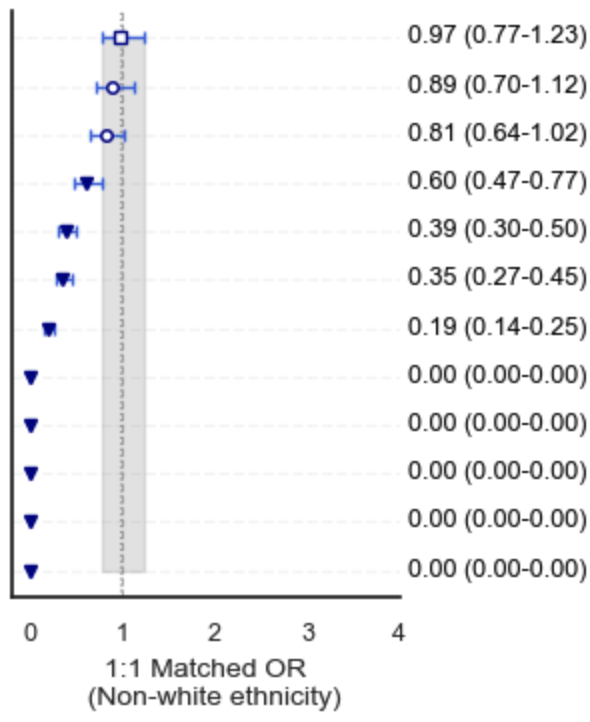 |
| Anxiety | 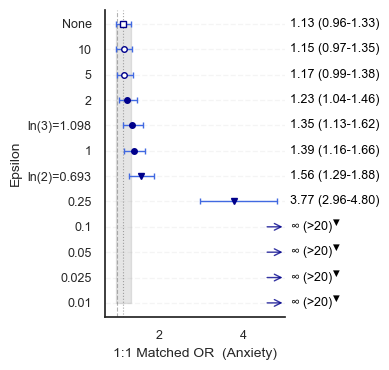 | 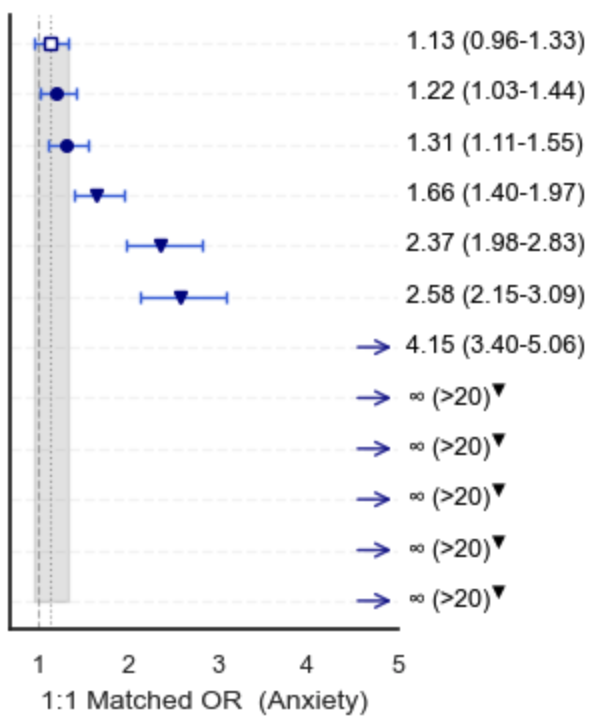 | 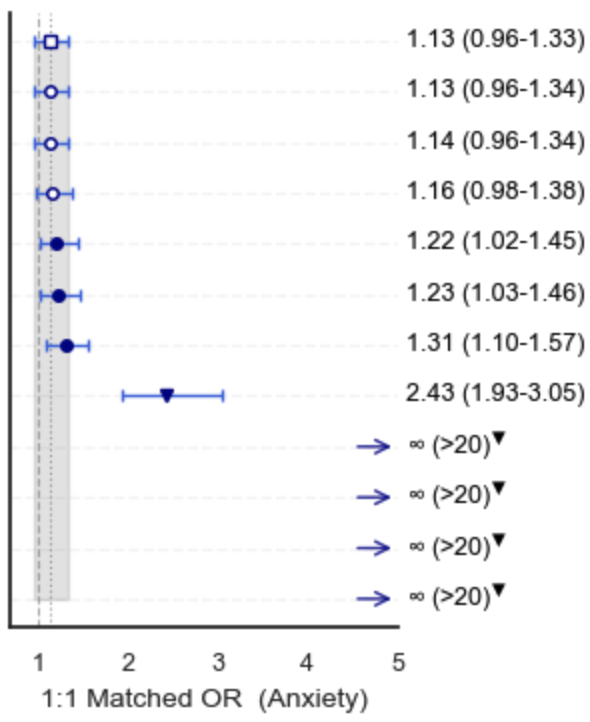 |
| 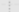■ Reference odds ratio (OR), p<0.05 □ Reference OR, not significant 95% Confidence interval of reference OR  ● Differentially private (DP) OR, p<0.05 ￮ DP OR, not significant ▼ DP OR, p<0.05, significantly different from reference OR  **Table continued on next page** | | | |

Table SB 6. (continued.) Reference vs differentially private 1:1 matched odds ratios with different random states (cases=2714, controls=2713).

| Covariate | A) Random sate 27 | B) Random state 88 | C) Random state 77 |
| --- | --- | --- | --- |
| BMI≥30 | 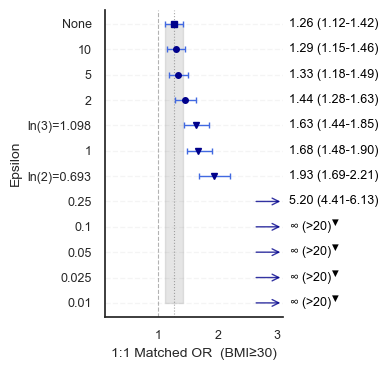 | 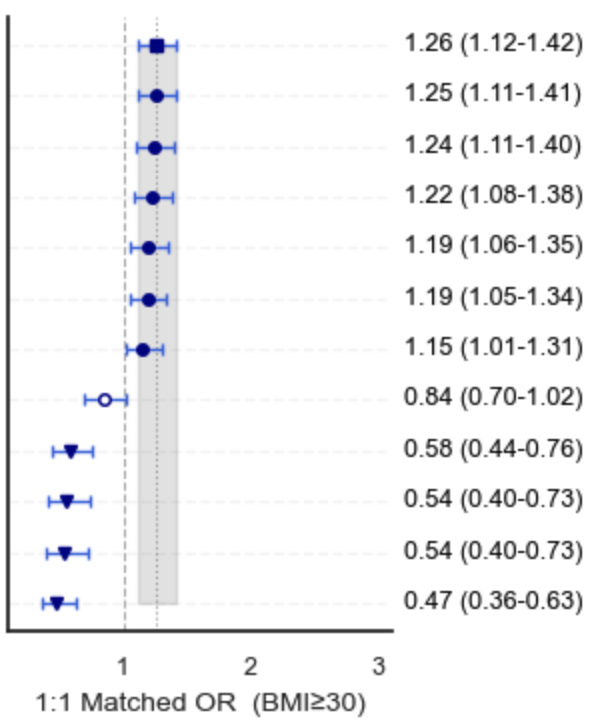 | 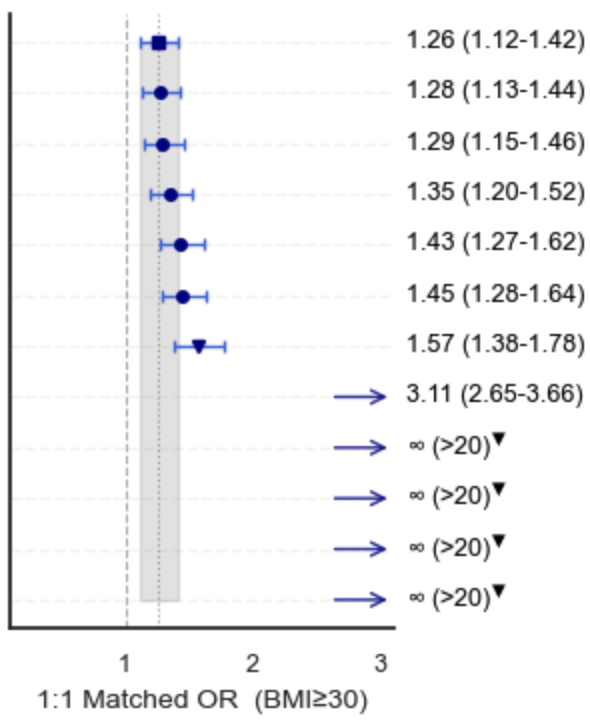 |
| CKD | 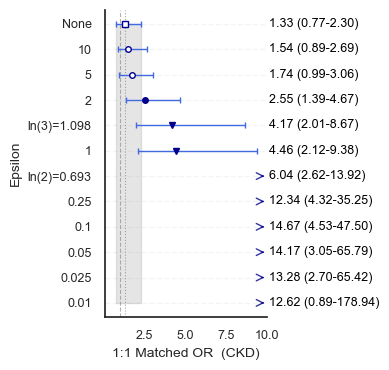 | 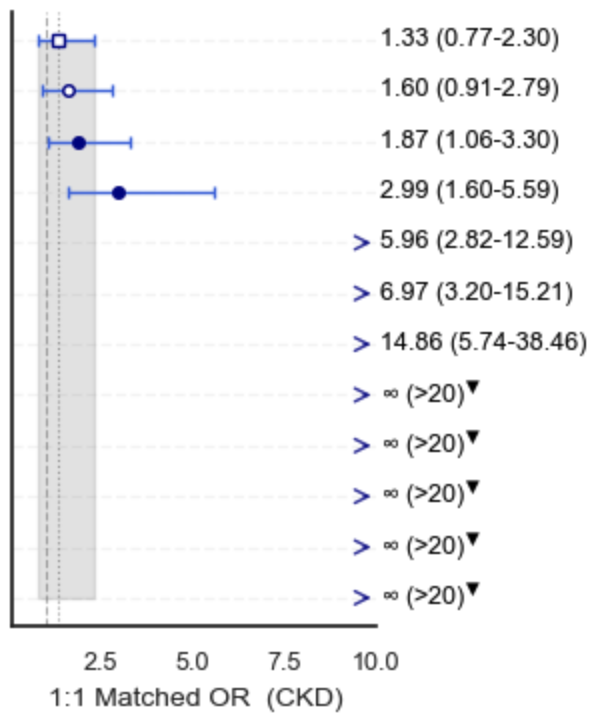 | 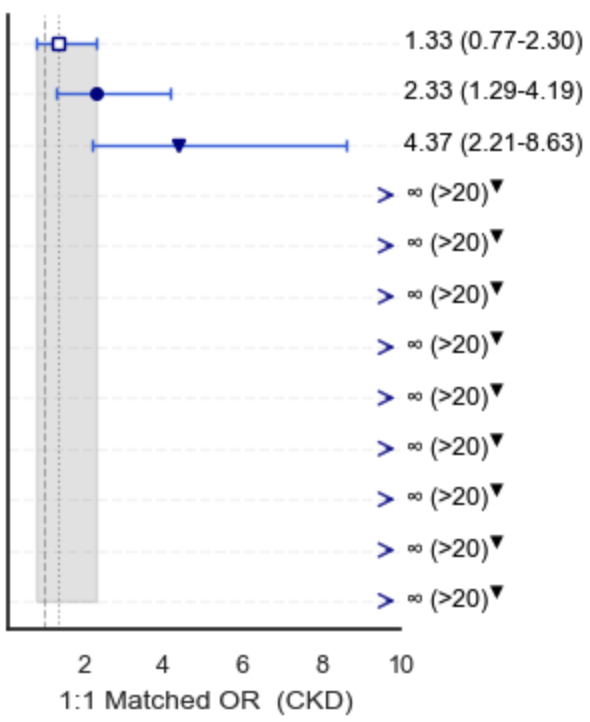 |
| COPD | 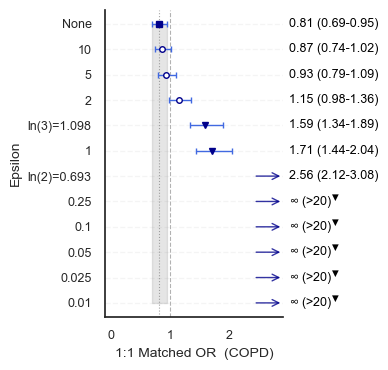 | 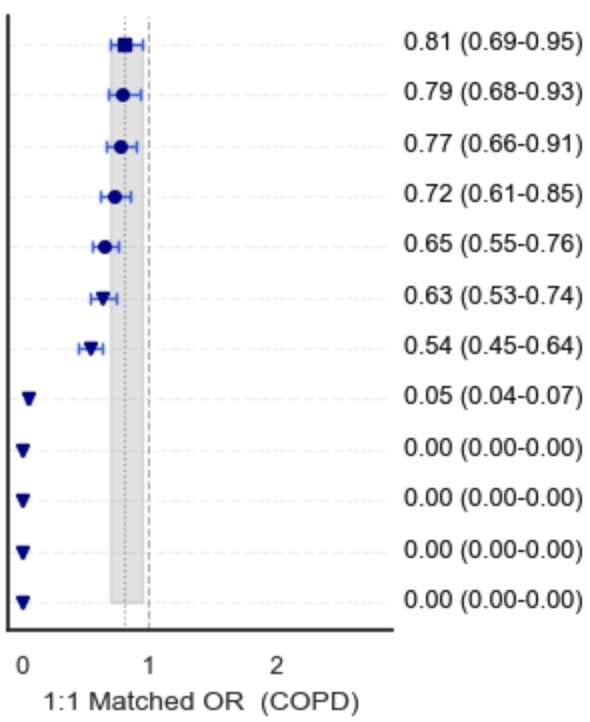 | 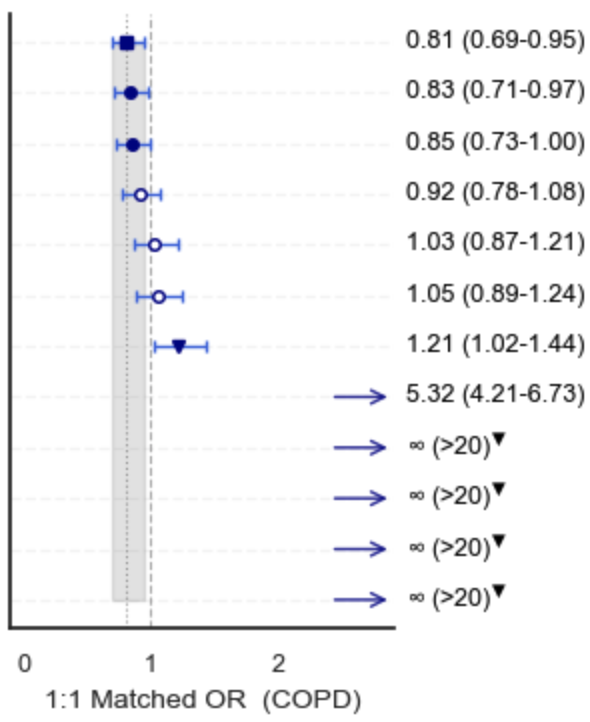 |
| CVD |  |  |  |
| ■ Reference odds ratio (OR), p<0.05 □ Reference OR, not significant 95% Confidence interval of reference OR  ● Differentially private (DP) OR, p<0.05 ￮ DP OR, not significant ▼ DP OR, p<0.05, significantly different from reference OR  **Table continued on next page** | | | |

Table SB 6. (continued.) Reference vs differentially private 1:1 matched odds ratios with different random states (cases=2714, controls=2713).

| Covariate | A) Random sate 27 | B) Random state 37 | C) Random state 77 |
| --- | --- | --- | --- |
| Diabetes |  |  |  |
| Hypertension |  |  |  |
| Cardinal symptoms |  |  |  |
| Pre-baseline pe year prescription of oral coricosteroid (OCS) |  |  |  |
| ■ Reference odds ratio (OR), p<0.05 □ Reference OR, not significant 95% Confidence interval of reference OR  ● Differentially private (DP) OR, p<0.05 ￮ DP OR, not significant ▼ DP OR, p<0.05, significantly different from reference OR  **Table continued on next page** | | | |

Table SB 6. (continued.) Reference vs differentially private 1:1 matched odds ratios with different random states (cases=2714, controls=2713).

| Covariate | A) Random sate 27 | B) Random state 88 | C) Random state 77 |
| --- | --- | --- | --- |
| Pre-baseline one year clinical exacerbation events |  |  |  |
| ■ Reference odds ratio (OR), p<0.05 □ Reference OR, not significant 95% Confidence interval of reference OR  ● Differentially private (DP) OR, p<0.05 ￮ DP OR, not significant ▼ DP OR, p<0.05, significantly different from reference OR | | | |

Table SB 7. Sensitivity analysis of model performance metrics under differential privacy with different random states (cases=2714, controls=19451).

|  | A) Random sate 27 | B) Random state 88 | C) Random state 77 |
| --- | --- | --- | --- |
| Brier score |  |  |  |
| ROC AUC |  |  |  |

Table SB 8. SHAP values of differentially private models in adjusted model (logistic regression) across epsilons (cases=2714, controls=19451).

| $\varepsilon=10$ | $\varepsilon=5$ | $\varepsilon=2$ |
| --- | --- | --- |
| $\varepsilon=\ln\left( 3 \right)=1.098$ | $\varepsilon=1$ | $\varepsilon=\ln\left( 2 \right)=0.693$ |
| **Table continues on next page** | | |

Table SB 8. (continued.) SHAP values of differentially private models in adjusted model (logistic regression) across epsilons (cases=2714, controls=19451).

| $\varepsilon=0.25$ | $\varepsilon=0.1$ | $\varepsilon=0.05$ |
| --- | --- | --- |
| $\varepsilon=0.025$ | $\varepsilon=0.01$ | Non-private |

Table SB 9. Summary of the sensitivity analyses.

| **Analysis** | **Description** | **Outcome** |
| --- | --- | --- |
| Unadjusted analysis | Crude odds ratios (ORs) were calculated using 2×2 contingency tables. IBM Diffprivlib’s two-dimensional histogram function was used to apply differential privacy (DP). | As shown in Table SB10, unadjusted ORs were generally higher than adjusted ORs, except for female sex and non-white ethnicity, indicating a stronger association with the outcome when adjusting for other factors. After applying differential privacy (DP), unadjusted ORs exhibited smoother changes with decreasing $\epsilon$, maintaining consistent directional trends with a fixed random seed. |
| Survivorship bias | A total of 42 patients with a death event during the study period were excluded to assess potential survivorship bias. | No notable changes were observed. |
| Random seeds | The adjusted and matched analyses were repeated using three additional random seeds. | As shown in Tables SB5-SB7, different random seeds influenced both the direction and magnitude of differentially private outputs. To mitigate p-hacking, random seeds should be predefined or results averaged across multiple seeds. |
| 1:1 matching with reduced covariates | A 1:1 matching was conducted, excluding asthma duration from the set of baseline covariates used for propensity score calculation. | The number of matched controls dropped from 2713 to 2712. No notable changes were observed in the outputs. |
| 1:2 matching | Matching was performed using a 1:2 case-to-control ratio, employing the same covariates for propensity score calculation as in the primary study. The results are shown in Table SB11. | Covariate balance was preserved, although the standardized mean difference (SMD) for asthma exacerbation in the previous year increased from 0.02 to 0.11. The OR for pre-baseline exacerbation increased from 0.94 (0.75-1.18) to 1.41 (1.14-1.73), likely due to K-Nearest Neighbor (KNN) matching and a 0.1 caliper. The patterns of changes in magnitude and direction observed in the primary analysis were similarly evident in this case. |
| 1:4 matching | Matching was performed using a 1:4 case-to-control ratio, employing the same covariates for propensity score calculation as in the primary study. The results are shown in Table SB12. | SMDs for pre-baseline exacerbation and COPD exceeded 0.2, indicating suboptimal balance between cases and controls. The OR for pre-baseline asthma exacerbation rose from 0.94 (0.75-1.18) to 2.59 (2.10-3.19), and the OR for COPD from 0.81 (0.69-0.95) to 1.54 (1.35-1.76). The patterns of changes in magnitude and direction observed in the primary analysis were similarly evident in this case. |
| Differentially private propensity scores and matching | DP was applied to propensity score calculation to evaluate its effects on covariate balance and the characteristics of matched controls, as shown in Tables SB13-SB18. | As shown in Table SB13, covariate balance deteriorated at ϵ = ln(2), resembling unbalanced matches at lower epsilon values. Comparisons between DP and non-DP controls revealed statistically significant differences in numerical covariates (e.g., age, asthma duration), even at higher epsilons (ϵ=10; Tables SB14–SB17). Binary covariates (Table SB18) remained stable at ϵ > ln(2) but showed increasing variability at lower epsilon values. |

Table SB 10. Reference and differentially private unadjusted odds ratios (OR) of risk factors across epsilons (cases=2714, controls=19451).

| A) Age≥60 | B) Female sex | C) Ethnicity (non-white) |
| --- | --- | --- |
| D) Anxiety | E) BMI≥30 | F) CKD |
| G) COPD | H) CVD | I) Diabetes |
| ■ Reference odds ratio (OR), p<0.05 □ Reference OR, not significant 95% Confidence interval of reference OR  ● Differentially private (DP) OR, p<0.05 ￮ DP OR, not significant ▼ DP OR, p<0.05, significantly different from reference OR  **Table continues on next page** | | |

*Table SB 10. (continued.) Reference and differentially private unadjusted odds ratios (OR) of risk factors across epsilons (cases=2714, controls=19451).*

| J) Hypertension | K) Cardinal symptoms | L) Pre-baseline OCS |
| --- | --- | --- |
| M) Pre-baseline exacerbation clinical | N) (scaled)Pre-baseline exacerbation clinical |  |
| ■ Reference odds ratio (OR), p<0.05 □ Reference OR, not significant 95% Confidence interval of reference OR  ● Differentially private (DP) OR, p<0.05 ￮ DP OR, not significant ▼ DP OR, p<0.05, significantly different from reference OR | | |

Table SB 11. Reference and differentially private 1:2 matched and adjusted ORs of risk factors across epsilons (cases=2714, controls=5334) .

| A) Age≥60 | B) Female sex | C) Ethnicity (n on-white) |
| --- | --- | --- |
| D) Anxiety | E) BMI≥30 | F) CKD |
| G) COPD | H) CVD | I) Diabetes |
| ■ Reference odds ratio (OR), p<0.05 □ Reference OR, not significant 95% Confidence interval of reference OR  ● Differentially private (DP) OR, p<0.05 ￮ DP OR, not significant ▼ DP OR, p<0.05, significantly different from reference OR  **Table continues on next page** | | |

*Table SB 11. (continued.) Reference and differentially private 1:2 matched and adjusted ORs of risk factors across epsilons (cases=2714, controls=5334) .*

| J) Hypertension | K) Cardinal symptoms | L) Pre-baseline OCS |
| --- | --- | --- |
| M) Pre-baseline exacerbation clinical |  |  |
| ■ Reference odds ratio (OR), p<0.05 □ Reference OR, not significant 95% Confidence interval of reference OR  ● Differentially private (DP) OR, p<0.05 ￮ DP OR, not significant ▼ DP OR, p<0.05, significantly different from reference OR | | |

Table SB 12. Reference and differentially private 1:4 matched and adjusted ORs of risk factors across epsilons (cases=2714, controls=9736) .

| A) Age≥60 | B) Female sex | C) Ethnicity (n on-white) |
| --- | --- | --- |
| D) Anxiety | E) BMI≥30 | F) CKD |
| G) COPD | H) CVD | I) Diabetes |
| ■ Reference odds ratio (OR), p<0.05 □ Reference OR, not significant 95% Confidence interval of reference OR  ● Differentially private (DP) OR, p<0.05 ￮ DP OR, not significant ▼ DP OR, p<0.05, significantly different from reference OR  **Table continues on next page** | | |

*Table SB 12. (continued.) Reference and differentially private 1:4 matched and adjusted ORs of risk factors across epsilons (cases=2714, controls=9736) .*

| J) Hypertension | K) Cardinal symptoms | L) Pre-baseline OCS |
| --- | --- | --- |
| M) Pre-baseline exacerbation clinical |  |  |
| ■ Reference odds ratio (OR), p<0.05 □ Reference OR, not significant 95% Confidence interval of reference OR  ● Differentially private (DP) OR, p<0.05 ￮ DP OR, not significant ▼ DP OR, p<0.05, significantly different from reference OR | | |

Table SB 13. Standardized mean difference (SMD) of non-private and differentially private propensity scores (age and Asthma duration are normalized).

| Non-private | $\varepsilon=10$ | $\varepsilon=5$ | $\varepsilon=2$ |
| --- | --- | --- | --- |
| $\varepsilon=\ln\left( 3 \right)=1.098$ | $\varepsilon=1$ | $\varepsilon=\ln\left( 2 \right)=0.693$ | $\varepsilon=0.25$ |
| $\varepsilon=0.1$ | $\varepsilon=0.05$ | $\varepsilon=0.025$ | $\varepsilon=0.$01 |
|  | | | |

Table SB 14. Distribution of the age in controls matched using non-DP vs DP propensity scores.

| $\varepsilon=10$ | $\varepsilon=5$ | $\varepsilon=2$ |
| --- | --- | --- |
| $\varepsilon=\ln\left( 3 \right)=1.098$ | $\varepsilon=1$ | $\varepsilon=\ln\left( 2 \right)=0.693$ |
| $\varepsilon=0.25$ | $\varepsilon=0.1$ | $\varepsilon=0.05$ |
| $\varepsilon=0.025$ | $\varepsilon=0.$01 |  |

Table SB 15. Distribution of asthma duration in controls matched using non-DP vs DP propensity scores.

| $\varepsilon=10$ | $\varepsilon=5$ | $\varepsilon=2$ |
| --- | --- | --- |
| $\varepsilon=\ln\left( 3 \right)=1.098$ | $\varepsilon=1$ | $\varepsilon=\ln\left( 2 \right)=0.693$ |
| $\varepsilon=0.25$ | $\varepsilon=0.1$ | $\varepsilon=0.05$ |
| $\varepsilon=0.025$ | $\varepsilon=0.$01 |  |

Table SB 16. Differences in the mean of age between controls matched using non-DP vs DP propensity scores.

| **Epsilon** | **T-statistics** | **T-test p-value** | **U-statistics** | **Mann-Whitney p-value** |
| --- | --- | --- | --- | --- |
| 10 | 389.95** | <0.0001 | 7360369.00** | <0.0001 |
| 5 | 390.05** | <0.0001 | 7363082.00** | <0.0001 |
| 2 | 390.11** | <0.0001 | 7363082.00** | <0.0001 |
| $\ln\left( 3 \right)=$1.098 | 390.17** | <0.0001 | 7363082.00** | <0.0001 |
| 1 | 390.12** | <0.0001 | 7360369.00** | <0.0001 |
| $\ln\left( 2 \right)=$0.693 | 390.17** | <0.0001 | 7360369.00** | <0.0001 |
| 0.25 | 390.52** | <0.0001 | 7363082.00** | <0.0001 |
| 0.1 | 390.60** | <0.0001 | 7363082.00** | <0.0001 |
| 0.05 | 390.57** | <0.0001 | 7363082.00** | <0.0001 |
| 0.025 | 390.49** | <0.0001 | 7363082.00** | <0.0001 |
| 0.01 | 8.306** | <0.0001 | 4142737.00** | <0.0001 |

*p<0.05, **p<0.01

Table SB 17. Differences in the mean of asthma duration between controls matched using non-DP vs DP propensity scores.

| **Epsilon** | **T-statistics** | **U-statistics** |
| --- | --- | --- |
| 10 | 59.66** | 7360369.00** |
| 5 | 59.67** | 7363082.00** |
| 2 | 59.64** | 7363082.00** |
| $\ln\left( 3 \right)=$1.098 | 59.60** | 7363082.00** |
| 1 | 59.60** | 7360369.00** |
| $\ln\left( 2 \right)=$0.693 | 59.58** | 7360369.00** |
| 0.25 | 59.57** | 7363082.00** |
| 0.1 | 59.57** | 7363082.00** |
| 0.05 | 59.63** | 7363082.00** |
| 0.025 | 59.66** | 7363082.00** |
| 0.01 | -0.418 | 3642211.00 |

*p<0.05, **p<0.01

Table SB 18. Differences in binary confounders in controls matched using non-DP vs DP propensity scores.

| **Epsilon** | **Chi-square statistics, p-value** | | | | | |
| --- | --- | --- | --- | --- | --- | --- |
|  | **Female sex** | **Non-white ethnicity** | **COPD** | **Current smoker** | **Pre baseline OCS prescription** | **Pre baseline clinical indication of exacerbation** |
| 10 | 0.494 | 0.003 | 0.013 | 0.394 | 0.052 | 0.003 |
| 5 | 0.214 | 0.012 | 0.111 | 0.029 | 0.030 | 0.343 |
| 2 | 0.002 | 0.252 | 0.067 | 0.372 | 0.000 | 0.123 |
| $\ln\left( 3 \right)=$1.098 | 0.025 | 0.375 | 0.311 | 1.377 | 0.004 | 1.265 |
| 1 | 0.423 | 0.115 | 0.204 | 0.330 | 0.050 | 1.546 |
| $\ln\left( 2 \right)=$0.693 | 1.390 | 0.000 | 0.874 | 4.185* | 0.029 | 3.718 |
| 0.25 | 10.384 | 0.311 | 5.720* | 1.027 | 0.681 | 34.479** |
| 0.1 | 18.922** | 0.000 | 106.807** | 0.029 | 7.093** | 87.201** |
| 0.05 | 16.166** | 0.216 | 158.266** | 2.221 | 30.561** | 96.528** |
| 0.025 | 17.748** | 0.031 | 183.324** | 6.741** | 28.919** | 64.716** |
| 0.01 | 17.517** | 4.184* | 178.707** | 7.967** | 44.882** | 80.255** |

*p<0.05, **p<0.01
